# A global view of *Staphylococcus aureus *whole genome expression upon internalization in human epithelial cells

**DOI:** 10.1186/1471-2164-8-171

**Published:** 2007-06-14

**Authors:** Christian Garzoni, Patrice Francois, Antoine Huyghe, Sabine Couzinet, Caroline Tapparel, Yvan Charbonnier, Adriana Renzoni, Sacha Lucchini, Daniel P Lew, Pierre Vaudaux, William L Kelley, Jacques Schrenzel

**Affiliations:** 1 Service of Infectious Diseases, University Hospital of Geneva, Department of Internal Medicine, 24 rue Micheli-du-Crest, CH-1211 Geneva 14, Switzerland; 2 Molecular Microbiology Group, Institute of Food Research, Norwich Research Park, Colney, Norwich, NR4 7UA, UK

## Abstract

**Background:**

*Staphylococcus aureus*, a leading cause of chronic or acute infections, is traditionally considered an extracellular pathogen despite repeated reports of *S. aureus *internalization by a variety of non-myeloid cells *in vitro*. This property potentially contributes to bacterial persistence, protection from antibiotics and evasion of immune defenses. Mechanisms contributing to internalization have been partly elucidated, but bacterial processes triggered intracellularly are largely unknown.

**Results:**

We have developed an *in vitro *model using human lung epithelial cells that shows intracellular bacterial persistence for up to 2 weeks. Using an original approach we successfully collected and amplified low amounts of bacterial RNA recovered from infected eukaryotic cells. Transcriptomic analysis using an oligoarray covering the whole *S. aureus *genome was performed at two post-internalization times and compared to gene expression of non-internalized bacteria. No signs of cellular death were observed after prolonged internalization of *Staphylococcus aureus *6850 in epithelial cells. Following internalization, extensive alterations of bacterial gene expression were observed. Whereas major metabolic pathways including cell division, nutrient transport and regulatory processes were drastically down-regulated, numerous genes involved in iron scavenging and virulence were up-regulated. This initial adaptation was followed by a transcriptional increase in several metabolic functions. However, expression of several toxin genes known to affect host cell integrity appeared strictly limited.

**Conclusion:**

These molecular insights correlated with phenotypic observations and demonstrated that *S. aureus *modulates gene expression at early times post infection to promote survival. *Staphylococcus aureus *appears adapted to intracellular survival in non-phagocytic cells.

## Background

*Staphylococcus aureus *is a versatile pathogenic bacterium capable of rapidly developing or acquiring multiple antibiotic resistances, and is now recognized as a worldwide health problem [1]. *S. aureus *is responsible for a wide spectrum of human and animal diseases, ranging from benign skin infections to severe diseases, such as arthritis, osteomyelitis, endocarditis or fatal sepsis [2]. Acute infections are related to the organisms' capacity to secrete a plethora of exotoxins [3,4] and catabolic enzymes [2,5], as documented previously in different experimental models of acute infections [6-8]. *S. aureus *is also responsible for chronic diseases such as osteomyelitis [9], rhinosinusitis [10], or otitis [11]. These infections are difficult to eradicate and often relapse even after prolonged and adapted antibiotic therapy [12,13], suggesting that *S. aureus *has developed specific strategies for intracellular persistence. In addition, anti-infective agents commonly used for the treatment of *S. aureus *infections could enhance selection of invasive intracellular strains [14].

In contrast to other persistent human pathogens, *S. aureus *is not traditionally considered as an intracellular pathogen [15]. Nevertheless, substantial evidence strongly supports that *S. aureus *can be internalized and survive in a variety of non-professional phagocytic cells *in vitro *[2,16-18] and *in vivo *[19,20]. The endocytic uptake of *S. aureus *by non-myeloid cells involves active cellular processes that depend upon F-actin polymerization and is similar in many respects to that observed in professional phagocytes [17]. Whereas entero-invasive pathogens utilize secretion systems to actively induce their own uptake by the host cell, internalization of *S. aureus *by non-professional phagocytes shows similar efficiency *in vitro *with live or killed bacteria [17,21]. The mechanism relies on an interaction between fibronectin binding protein and host-cell α5β1 integrins [17,22,23]. The role of other bacterial surface proteins like clumping-factor A or host cell Src kinase also appears important in the mediation of *S. aureus *uptake and intracellular persistence [18,24]. After internalization, the behavior of the bacterium varies according to cell-line or bacterial strain. For example, some authors reported active intracellular bacterial replication within vacuoles [25] or rapid bacterial escape from vacuole and induction of cellular apoptosis [26-28], while others described persistence for several days before induction of escape processes [29]. The production of α-toxin appears correlated with the induction of apoptosis [27,30,31]. Regulation of α-toxin expression is complex and involves multiple regulators that include *agr*, *sarA *homologues, or *svrA *[32-35].

Molecular details that govern *S. aureus *extended persistence are largely unknown. Metabolic alterations leading to small colony variant (SCV) microorganisms are one possibility that has been described [36-38]. Such *S. aureus *variants were recently shown to efficiently invade endothelial cells *in vitro *and display a markedly higher content in fibronectin-binding proteins than the parental strain [39]. SCVs display a major alteration in their ability to produce or export exotoxins [36] and reveal extensive changes in their global regulatory network [40]. Overall this persistent behavior, possibly related to alteration of regulatory networks, appears compatible with the property of *S. aureus *to generate relapsing infections even years after a first episode was apparently cured [36,41].

Several studies have examined details of cellular responses after *S. aureus *internalization in either phagocytic or non-phagocytic cells [42,43]. However, little is known about bacterial gene expression upon cellular internalization. Recent efforts in high throughput sequencing have contributed to the elucidation of numerous bacterial genomes. To date, eight fully annotated *S. aureus *genomes are publicly available [44-49] allowing the design of DNA microarrays to probe the bacterial transcriptome [50-54], or to catalogue and type variation among clinical isolates [53,55,56].

In this study, we describe an *in vitro *model where *S. aureus *is able to persist for up to two weeks in the absence of either cellular death or emergence of small colony variants. *S. aureus *gene expression changes between adherent bacteria and those arising 2 h and 6 h after cellular internalization were compared using a custom-designed and validated *S. aureus *oligoarray [53]. Profound shut-down of gene expression was observed shortly after internalization for bacterial metabolic functions and transport. This period was followed by resumption of transcriptional activity for metabolic and energy production functions correlating with moderate bacterial multiplication. These findings suggest that *S. aureus *extensively reprograms its transcriptome to adjust to the intracellular environment. Detailed study of these changes may help understanding the molecular mechanisms governing establishment and maintenance of persistent *S. aureus *infections and potentially helpful for the design of new antibacterial drugs.

## Results

### Infection model and bacterial RNA isolation

**Figure 1:**
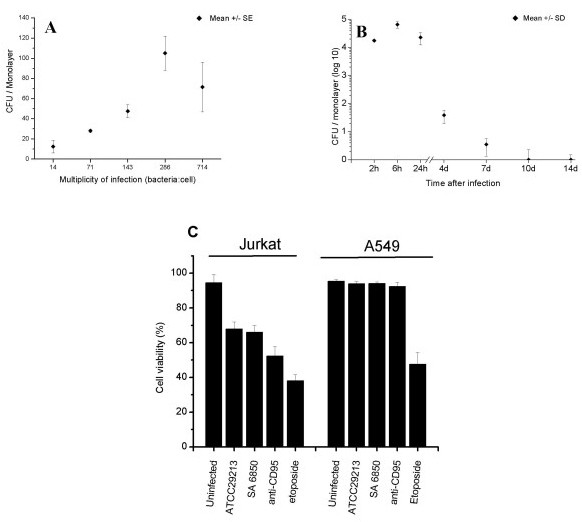
**Internalization of *S. aureus *6850 by A549 cells**. **A**. Dose-response of internalization assay performed by adding increasing number of bacteria to constant number of cells. The internalization assay was performed as described in the experimental section. After 2 h, cells were detached with trypsin lyzed with dilute Triton X-100, and then internalized bacteria were assessed by count of colony forming units (cfu). **B**. Intracellular survival assay of *S. aureus *over a two-week interval. Note that at times after 6 hours, viable bacterial counts decreased, though viable bacteria were recovered from monolayers even 2 weeks after internalization. All values are mean ± standard deviation (SD) of at least three independent experiments. **C**. Viability tests were performed after 24 hours of internalization using bacterial m.o.i. of 100:1, or the presence of anti-CD95 (1 μg/ml) or etoposide (100 μM final concentration) as pro-apoptotic controls.

We developed a model system that displayed protective survival of *S. aureus *6850 within human lung epithelial cells A549. Control experiments were performed to examine infection parameters and determine the saturation of internalization. Figure 1A shows bacterial internalization as a function of the multiplicity of infection (m.o.i). The profile describes a linear dose-response until saturation for m.o.i higher than 286 *S. aureus *per cell. We next examined the intracellular survival of strain 6850 as a function of time using the lysostaphin/gentamicin protection assay, as shown Figure 1B. We observed substantial intracellular survival during the first 24 hour post-internalization. Notably, we observed a 3-fold increase in viable bacteria recovered at 6 hours post internalization compared with 2 hours indicating most probably that bacterial replication, or completion of cell division, had occurred. Viable bacterial counts diminished slightly in the 6–24 h interval. A 3 log decrease in recovered bacteria was observed in the interval from 1–4 days post-infection indicating substantial infection clearance. Nevertheless, viable bacteria could be recovered in this model system up to 2 weeks post infection. Strain 6850 produces strong α-toxin amounts as revealed on blood agar plates as well as in strains recovered from the intracellular survival assay (data not shown). We observed no emergence of SCV at any time point in the assay. We conclude that the model reflects both a window of bacterial survival and replication as well as persistent infection and we focused in early time-points (2, 6 hours) for subsequent detailed study. Microscopic observations with trypan blue revealed no significant cytotoxicity as well as the absence of any cellular morphologic alteration during the first 14 days of co-incubation (Figure 1B). Subsequent viability assays were performed at a sub-saturating m.o.i. of 100:1 using strains 6850 or ATCC29213, previously described as cytotoxic in other cell lines [27,30,31]. Control Jurkat T cells revealed altered cellular viability in the presence of any of the two bacterial strains or by contact with pro-apoptotic compounds (Figure 1C). In contrast, a total absence of cellular toxicity was observed in A549 cells in the presence of either 6850 or ATCC strains. Similarly to previously published observation [57], anti-CD95 failed also to affect A549 viability whereas etoposide showed a significant impact on A549 viability (Figure 1C).

Purification of bacterial RNA from intracellular bacteria proved difficult due to significant excess of eukaryotic material. After protocol optimizations, the extraction and amplification procedures showed appreciable reproducibility (additional file 1) with Pearson correlation value of 0.9–0.93, an important parameter in microarray experiments [58,59]. Nevertheless, bacterial RNA preparations contained detectable contaminating RNA traces from host-cell. Absence of detectable cross-hybridization on the microarray with cellular RNA demonstrates the high specificity of the microarray for bacterial messengers resulting from the design strategy (data not shown).

### Analysis of gene expression changes in extracellular and adherent bacteria

**Figure 2:**
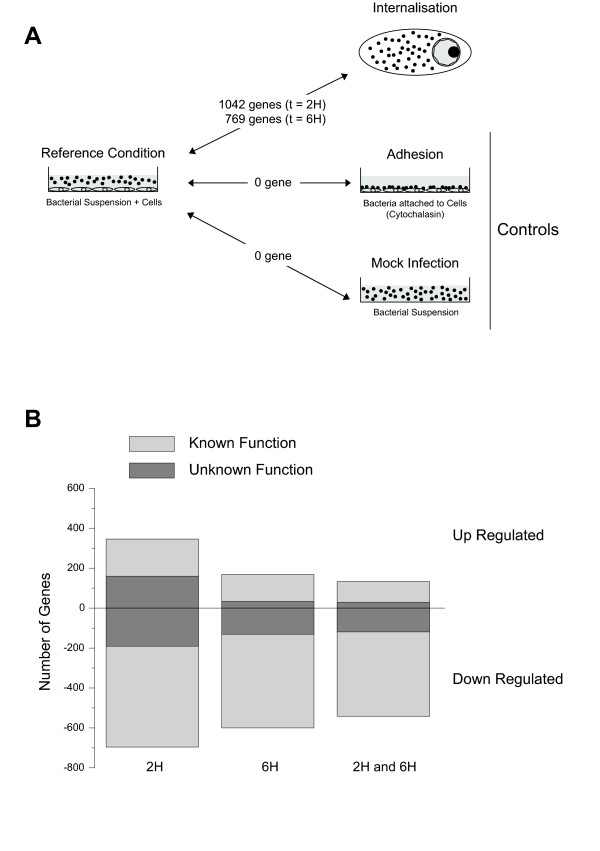
**Schematic representation of conditions subjected to microarray analysis and number of differentially expressed genes**. **A**. Similar expression profiles were observed for the comparisons between the reference condition and the control conditions consisting in i) mock infection (bacteria in cell culture medium) or ii) adherent bacteria (in the presence of cytochalasin D 1 μg/ml). On the opposite, an important number of genes were found differentially regulated between reference condition and internalized bacteria after 2 or 6 h of intracellular survival. See material and methods section for details. **B**. Numbers of genes found differentially regulated are indicated for each condition. The proportion of non-characterized genes annotated as genes with "unknown function" appears dark grey.

We first performed several microarray experiments to evaluate the impact of cell culture medium, presence of cells, presence of cytochalasin D, and bacterial attachment to cells, on the bacterial transcriptome expression. The experimental design and pairwise comparisons performed are depicted in Figure 2A. The following conditions were tested: i) bacteria propagated in invasive medium alone, ii) non-adherent bacteria cultivated in the presence of cells, iii) non-adherent bacteria recovered from infections in the presence of cytochalasin D, and iv) cell-adherent bacteria in the presence of cytochalasin D. No significant changes in gene regulation were recorded from all possible pair-wise comparisons. We conclude from this analysis that exposure of bacteria to our experimental parameters prior to cellular internalization does not result in detectable alteration of bacterial transcriptome. We then compared expression values obtained at time 0 in the culture medium against those obtained after 2 or 6 hours of internalization.

### Analysis of intracellular bacterial gene expression changes

Previous studies performed in our laboratory indicated that bacterial uptake by both A549 and MRC5 lung epithelial cells is a rapid process (< 30 minutes) [17]. From this result, as well as those presented in Figure 1B, we reasoned that 2 and 6 hour time points would reveal new information about bacterial transcriptomic program required for early essential adaptation to the intracellular environment. We therefore evaluated the transcriptome of *S. aureus *after 2 and 6 h post-internalization.

Comparison of gene expression between control (time 0) and 2 h or 6 h post-infection conditions revealed extensive modifications in bacterial gene regulation. The complete raw data set as imported from the array scanner as well as the 2 h, 6 h, and functional group cluster is available online [60].

Statistically significant changes in gene expression were found for 1042 and 766 genes after 2 h or 6 h of infection, respectively (including 338 and 165 hypothetical proteins). After 2 h of infection, 346 genes were up-regulated whereas 696 were down-regulated, while 169 genes were found up-regulated and 600 genes down-regulated 6 h post-infection (Figure 2B). The numbers of genes showing differential expression levels only for one of the two time-points were 89 and 362 for 2 or 6 h, respectively. Since all tested control conditions did not result in significant changes in gene expression, we conclude that these massive alterations in transcription profiles are triggered upon bacterial internalization.

### Clustering and functional group assignment

**Figure 3:**
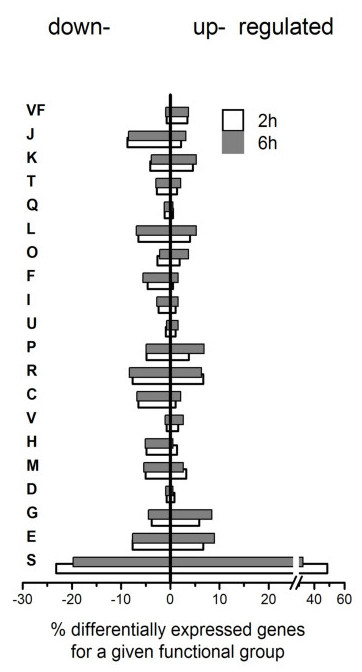
**Changes in gene expression 2 h and 6 h after internalization shown by functional categories**. Percent changes at 2 h (white) and 6 h (grey) was calculated by dividing the number of significantly changed genes by total genes at the given time point. Genes were assigned functional groups using annotated public database and metabolic pathways databases (COG). Categories are: E: Amino acid transport and metabolism, G: Carbohydrate transport and metabolism, D: Cell cycle control, cell division, chromosome partitioning, M: Cell wall/membrane/envelope biogenesis, H: Coenzyme transport and metabolism, V: Defense mechanisms, C: Energy production and conversion, R: General function prediction only, P: Inorganic ion transport and metabolism, U: Intracellular trafficking, secretion, and vesicular transport I: Lipid transport and metabolism, F: Nucleotide transport and metabolism, O: Posttranslational modification, protein turnover, chaperones, L: Replication, recombination and repair, Q: Secondary metabolites biosynthesis, transport and catabolism, T: Signal transduction mechanisms, K: Transcription, J: Translation, ribosomal structure and biogenesis, and VF for virulence factor.

Differentially expressed genes were clustered by functional group categories according to the COG classification [61] (additional file 2). An additional category consisting in documented virulence factors was added (Table 1 and Figure 3), according to established criteria [62]. Overall, the general trend revealed a substantial down-regulation of genes involved in metabolic functions (additional file 3). For example, among the 55 ORFs encoding for ribosomal proteins, > 40 were found down-regulated at 2 h, or 6 h. This reduction concerned also the totality of the *atp *operon (*atpA *to *atpH*) responsible for ATP synthesis and genes involved in nucleotide and amino acid metabolism. Note however that the majority of these genes were up-regulated between 2 and 6 hours. Similar responses were also observed for numerous genes involved in sugar metabolism and energy production. A more balanced picture was observed among signal transduction and transcription regulators (additional file 3). Changes in gene expression of hypothetical reading frames were observed to be equally distributed between down- and up-regulated genes. Among genes that were up-regulated, we noted a significant number of targets involved in iron metabolism and other inorganic ions (additional file 3) contributing to oxidative stress protection. Among the most significantly up-regulated genes, we observed defense genes, such as *sodA*, *ahp *and also transcription regulators of virulence factors (Table 1), RNAIII, *sarS *(also named *sarH1*) and *svrA *(SA0323 or SAV0334 according to the annotation of *S*.*aureus *N315 and Mu50, respectively) which are known to modulate the expression of the *agr *locus [35,63]. Another category showing important changes in gene expression was transporters (additional file 3). More than 40 genes were found significantly differentially regulated including transporters of iron, sugars, nucleotides and amino-acids. Importantly, expression of toxic virulence factors and α-hemolysin were found either down-regulated, or unchanged, compared to the control conditions (Table 1). Finally, the number of genes involved in biosynthesis or degradation of bacterial envelopes showed contrasted levels of expression after internalization as compared to extracellular control bacteria (see Materials and Methods). Whereas cell-wall metabolism enzymes (the autolysin *lytM*, and several amidases *lytA*, *sgtA and *SA1759) showed drastic up-regulation, transpeptidase, acid techoic synthesis genes, the *mur *operon and several *cap *genes showed massive down-regulation after internalization (additional file 3).

**Table 1:** Genes differentially expressed involved in virulence

	Common	Organism	2 h fold change	6 h Fold change	Protein name
**SA2423**	*clfB*	N315	0.17	0.19	Clumping factor
**SA2291**	*fnbA*	N315	3.23		fibronectin binding proteins
**SA2290**	*fnbB*	N315		3.83	fibronectin binding proteins
**MW1940**	*hlb*	MW2	3.08		β-hemolysin
**SAS065**	*hld*	N315	3.05		δ-hemolysin
**SA2209**	*hlgB*	N315		3.12	γ-hemolysin
**SA2461**	*icaB*	N315	3.75		Intercellular adhesion proteins
**SA1638**	*lukE*	N315	3.06		leukotoxin LukE
**SA1812**	*SA1812*	N315	0.14	0.22	synergohymenotropic toxin precursor *S. intermedius*
**SA0521**	*sdrE*	N315	0.20	0.29	Ser-Asp rich proteins
**SA1817**	*sec3*	N315	3.41		enterotoxin type C3
**MW0759**	*sec4*	MW2	3.60	4.76	enterotoxin C precursor protein
**SA1642**	*seg*	N315	4.90	3.70	extracellular enterotoxin type G precursor
**SA1816**	*sel*	N315	3.46	3.09	extracellular enterotoxin L
**SA1648**	*seo*	N315	3.94	6.33	enterotoxin SeO
**SA1009**	*set1*	N315	3.73		exotoxin 1
**SA0389**	*set13*	N315	3.52	3.45	exotoxin 13
**SA0390**	*set14*	N315	3.77	3.73	exotoxin 14
**MW0394**	*set26*	MW2		4.95	exotoxin homolog [Genomic island nu Sa alpha2]
**SA1011**	*set3*	N315	5.00	4.69	exotoxin 3
**SA0107**	*spa*	N315	0.15	0.10	Protein A
**SA0901**	*sspA*	N315	0.14	0.11	V8 protease
**SA0899**	*sspC*	N315	3.22		Staphopain
**SA1819**	*tst*	N315	3.80	4.10	Toxic shock syndrome toxin-1
**SA1645**	*yent1*	N315		3.18	enterotoxin Yent1

**Figure 4:**
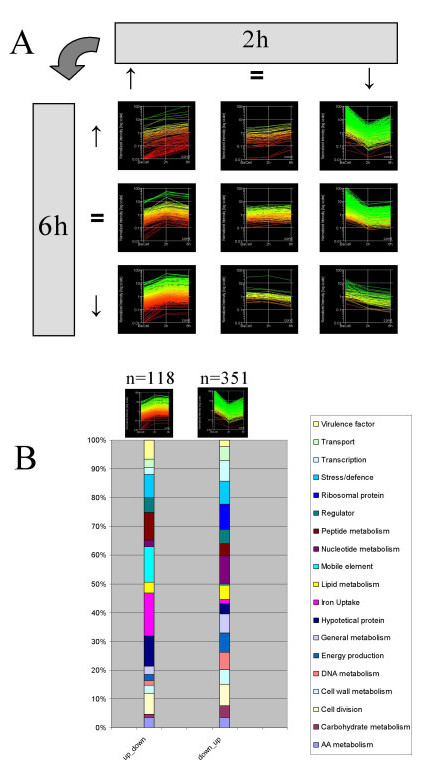
**Global pattern of expression**. Patterns of gene expression changes are shown for each of nine possible categories. The most abundant categories (B) were analyzed in depth after grouping by gene function. Numbers of genes in these two categories are also shown.

Based on the time-points studied, two major regulation profiles were found composed of genes showing: i) large down-regulation after 2 h, then a slight increase at 6 h, and ii) up-regulation at 2 h then only marginal change at 6 h. These two categories are presented in Figure 4A in the upper-right and lower-left corners, respectively. Grouping by functional category revealed that the first expression profile is mainly composed of mobile elements, transporters, regulators and iron metabolism (Fig. 4B). The other categories were mainly composed of genes involved in nutrient transport, general and nucleotide metabolism, and ribosomal proteins, but also in DNA replication and carbohydrate metabolism (Fig. 4B).

Analysis of gene expression changes in the interval 2 h-6 h post-infection. To examine gene expression changes occurring during its intracellular passage, we extracted a list of genes that were differentially expressed (determined by ANOVA) between times 2 h and 6 h after internalization. A striking up-regulation of gene expression was observed at 6 h post-infection in most functional categories, consistent with bacteria readapting their energy production, intermediary metabolism, protein synthesis, and nucleotide biosynthesis. This finding is consistent with metabolic restart necessary for the observed bacterial doublings in the 2–6 h time interval (Figure 1B).

### qPCR validation of array data for selected genes

**Figure 5:**
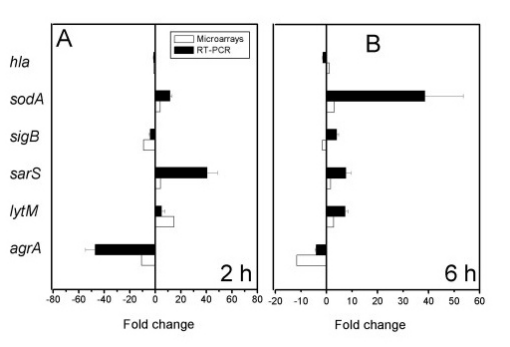
**Quantitative PCR validation of microarray data**. Dynamic of gene expression changes is shown as fold change by real-time PCR (black bars), or by microarray determination (white bars), at 2 h (A) or 6 h (B) post-infection. Data are mean ± SD of triplicate measurements from 2 independent experiments.

As an independent measure of differential gene expression, we examined the relative levels of 6 genes selected from different functional categories by quantitative real-time PCR showing up- or down-regulation or unchanged level of expression. As previously established in other studies, the absolute magnitudes of normalized signals reflects the broader dynamic range of the qPCR method compared to microarray measurements [53,64]. The results are shown in Figure 5 and are correlated in terms of up- or down-regulation with microarray determinations. Notably, the data confirms the observed down-regulation of *agr *expression correlated with the absence of *hla *transcription. Congruent changes were observed for *lytM*, *sarS *and *sodA *that showed up-regulation of their expression as previously recorded using microarrays (Figure 5).

## Discussion

Recent efforts in the field of high-throughput sequencing contributed to the public release of numerous bacterial genomes of medical importance. This information allowed the development of microarrays able to monitor gene expression changes at the genome scale. We report here the utilization of a microarray covering the whole genome of *Staphylococcus aureus *to evaluate the behavior of the bacterium upon cellular internalization, a phenomenon potentially contributing to bacterial protection against host defenses, and important in the context of chronic infections. Our overall findings are in agreement with previous studies utilizing experimental cellular models of bacterial internalization, using either intracellular or facultative intracellular pathogens. If the ability of *S. aureus *to persist in different *in vitro *models is widely accepted, the capacity of *S. aureus *to escape from host-defenses and persist intracellularly *in vivo *is still debated [15]. In this respect, the call to document "the presence of staphylococci within non-professional phagocytes at sites of infection in clinicopathological tissues" [15] has been recently demonstrated by Clément and colleagues, who identified *S. aureus *residing in human nasal epithelial cells *in vivo*, a probable cause of recurrent rhinosinusitis infection [19]. This *in vivo *survival capacity was further documented in the same population and shown to constitute a significant risk factor for recurrent episodes of rhinosinusitis refractory to antimicrobial and surgical therapy [65]. Similar behavior was also observed in an experimental model of mastitis [20].

In our model, the presence of cells or the attachment of bacteria to A549 cells (in the presence of cytochalasin) showed limited impact on bacterial transcriptome. This observation is in accordance with previous reports from our group showing that no active bacterial process is require for internalization [17]. With respect to bacterial pathogenesis, this observation suggests that *S. aureus *internalization is probably not rare as it requires only a minimal number of bacterial factors [22]. In contrast to other microorganisms known to invade eukaryotic cells and rapidly divert cellular compounds and energy to actively multiply [66], our study indicates that upon cellular internalization, *S. aureus *transiently shuts down most of its metabolic functions and adopts an apparent "dormant state" without extensively initiating classic starvation or stress process. These profound changes in the bacterial transcriptome affect approximately 40% of all putative ORFs identified in the genomic sequence of the bacterium and appear related to an extensive adaptation to the new environment constituted by the intracellular compartment. The majority of genes involved in energy production (citrate and ATP cycles, pyruvate metabolism, and oxidative phosphorylation) were drastically reduced after 2 h internalization. Consequently, > 75% of *S. aureus *ribosomal proteins showed a significant down-regulation, which constitutes a common trait with streptococcal exposition to endothelial cell [67], another Gram positive pathogen able to invade non-phagocytic cells before inducing apoptosis [68]. For these 2 pathogens, metabolic and cellular division machineries appear strongly down-regulated rapidly after internalization [67]. This situation is markedly different from that observed in invasive intracellular pathogens such as *Salmonella *[69], *Shigella *[66,70], or *Pasteurella *[71], which show up-regulation of most cellular processes upon internalization and are able to multiply actively before disseminating to other cells [69,70]. In our model, a significant increase in colony forming unit counts was observed between 2 and 6 h of intracellular persistence. This cellular multiplication parallels up-regulation of *lytM*, as well as that of numerous amidases involved in cell-wall processing [72]. Concurrently, a slight up-regulation of cell-wall and techoic acid synthesis genes was observed between these time points, suggesting evidence of intracellular replication or completion of cell division, as observed in the context of *S. aureus *phagocytosis by neutrophils [43]. This metabolic evolution was paralleled by a massive up-regulation of numerous transporters. In our study, more than 40 transporters, involved in inorganic ions, nucleotides or peptides, or urea were found significantly up-regulated. Transporters involved in metabolic pathways contribute to the direct rerouting of cellular resources by the internalized bacterium. Illustrative examples are the up-regulation of *uhpT *(observed at 2 and 6 h internalization), allowing glucose-6-phosphate acquisition, as observed during internalization of other bacterial species [66,70] or during *in vitro *[73] or intracellular exposition of *S. aureus *to acidic environments [43]. *S. aureus *genome contains 7 putative iron transporters [74] that are important mediators of bacterial virulence. Iron transporters are typically up-regulated in various internalized invasive pathogens [67,74,75]. In our study, *isdF*, SA0567 and particularly *bitC*, were drastically up-regulated in *S. aureus*, thus providing probably sufficient amounts of iron from the intracellular medium, whereas all genes composing the *fhu *operon, encoding ferric hydroxamate assimilation proteins were down-regulated, in accordance with their function of iron transporters under specific environmental conditions [76].

Intracellular survival can potentially expose the bacterium to cellular defense mechanisms such as oxygen radicals. In our study, the number of up-regulated genes involved in stress protection or bacterial defense revealed surprisingly low. Except for *sodA *and *cysM*, whose products protect bacterial cell against oxidative stress [77], a majority of genes involved in oxidative stress response appeared down-regulated. This finding is markedly different than the behavior of *Shigella *[66], but consistent with previous reports about *S. pneumoniae *[67]. Note however that experiments performed in A549 cells failed to detect production of reactive oxygen species upon bacterial uptake (not shown). Internalized *S. aureus *appeared to solicit protective functions but in a different context than that requiring preservation of the cellular machinery integrity as described by Voyish in the context of resistance to neutrophil phagocytosis [43]. In non-phagocytic cells, the expression of capsule operon, Clp proteases family and chaperones, as well as their known regulators *ctsR *and *sigB *[78], potentially conferring survival against toxic cellular metabolites was drastically down-regulated.

The expression of numerous bacterial virulence factors is modulated through complex networks of regulatory molecules including two-component systems and global regulators such as *agr *or *sar*. Multiple regulatory pathways contributed not only to the rapid adaptation of the bacteria to environmental changes but also to the regulation of toxins or cell-surface components expression during bacterial growth-phases [79,80]. In our system, upon contact with cell monolayers, strain 6850 was in early exponential phase of growth where *agr *activity is limited. Following internalization and metabolic restart, general metabolic functions appeared up-regulated in the absence of *agr *induction. In addition, internalized bacteria showed an increase in expression of *sarS *and *svrA *simultaneously to a marked down-regulation of *saeRS *genes, which are important compounds known to modulate *agr *expression [35,63]. In parallel to a moderately increased expression of RNAIII, a compound known to induce *agr *expression *in vitro*, this regulation pattern results in the reduced expression of *hla *that potentially mediates apoptosis induction in some systems [25,27,30]. This uncoupling between *agr *and RNAIII expression has been previously observed *in vivo *in an experimental model of endocarditis [81]. The restricted level of *hla *expression suggests a different regulation behavior under these experimental conditions or the action of another unknown regulator. The latter hypothesis is further supported by the results of Goerke [32], who showed that mutation in *agr *and *sar *had no consequence for *hla *expression *in vivo*. This unknown target is probably not *sigB*, a regulator found drastically down-regulated upon internalization as well as the totality of related *rsb *partners in our model. *SigB *has been recently suggested as a potential partner involved in the persistence of SCVs recovered from cystic fibrosis [82]. In our study, we failed to detect such SCVs as *S. aureus *recovered after 2 weeks of internalization yielded significant hemolysis when plated on blood agar. However, this observation does not rule out the emergence of SCVs in our model as this phenotype appears able to revert spontaneously [83]. These observations are directly related to the absence of cytotoxicity on A549 cells even after two weeks of infection, an observation common between our study and previous work using SCVs [84].

In contrast to the behavior of internalized SCVs, Bantel and colleagues reported 6850-evoked induction of apoptosis using Jurkat T cells [30], which is consistent with our control assays confirming that strain 6850 was able of provoking cytotoxic effects. In addition, Qazi and colleagues reported the induction of *agr *and bacterial escape from endosomal vesicles rapidly after internalization using *S. aureus *RN6390 and MAC-T [85]. On the contrary, Kahl reported that i) rifampicin-treated bacteria failed to induce apoptosis and that ii) Cowan I -a strain that failed to multiply in epithelial cells- does not show appreciable cytotoxic effects [25]. These observations are important concerning *S. aureus *pathogenicity and suggest that certain combinations of host cells and bacteria can result in different biological outcomes. Altogether, results obtained with different models strongly suggest that the internalization of *S. aureus *is a key factor in chronic infections, as intracellular survival occurs in cells showing a long lifespan such as fibroblasts or epithelial cells. *S. aureus *intracellular survival appears related to its capability to adopt a discrete behavior instead of actively duplicating. *S. aureus *then benefits from natural or programmed cell death to re-emerge and trigger another episode of infection, leading to chronicity.

## Conclusion

Internalization of *S. aureus *in non-phagocytic cells explaining its resistance properties against cellular or humoral host defenses is an attractive hypothesis relying on its particular ability to generate chronic diseases. In our model of cellular persistence, *S. aureus *strain 6850 is able to reside intracellularly for prolonged period of time. Microarray experiments provide a global picture of genes possibly involved in *S. aureus *pathogenesis, and in particular, the capacity of the bacterium to diminish most of its metabolic functions, despite soliciting numerous transporters allowing maintenance of vital functions but limiting its multiplication. Limited bacterial growth and expression of regulators of virulence factors leads to a finely controlled expression of cell-active toxins insuring intracellular bacterial survival. Global bacterial responses upon internalization appears complex but in accordance with the capacity of the bacterium to reside intracellularly. Finally, the study has uncovered dozens of potential targets for anti-infective therapeutics.

## Methods

### Bacterial strains and cell culture

A549 cells (ATCC CCL-185, human lung adenocarcinoma) were maintained under 5% CO_2 _in minimal essential medium (MEM, Gibco) supplemented with 10% heat-inactivated fetal calf serum (Gibco) and 100 U penicillin/ml. Two days prior to infection, 6 × 10^6 ^cells were seeded in 10 cm wells yielding weakly confluent monolayers (2 × 10^7 ^cells/well) at the time of infection. *S. aureus *6850 is derived from multifocal osteomyelitis, is fully pigmented, and maintains a stable virulence phenotype [86]. Overnight cultures in Mueller-Hinton broth (MHB, BD-Difco) were diluted with fresh media and grown to early log phase for 3 hours. Cells were washed twice in PBS, filtered (5 μm, Sartorius) to reduce aggregation and re-suspended in MEM at 1 × 10^9 ^cells/ml. Jurkat T cells (kind gift of D. Trono, Univ. of Geneva) were cultivated in RPMI (Gibco) supplemented with 10% heat-inactivated fetal calf serum (Gibco) and 100 U penicillin/ml.

### Cell infection and control conditions

Three hours before infection, A549 cells were washed once with MEM, then placed in 5.5 ml invasion medium (IM) consisting of MEM enriched with 10% human albumin (ZLB Biopharma). Infections were initiated with a multiplicity of infection (m.o.i. 100:1 bacteria/cells) using bacteria in early logarithmic growth phase (OD_600 _= 0.2). Cultures were incubated 30 min at 37°C in 5% CO_2 _with 2 × 10^9 ^washed bacteria to allow adhesion and internalization. Non-adherent bacteria were collected (control bacteria) and fixed with ice cold acetone/EtOH (1:1). Infected monolayer cells were washed twice with MEM to remove residual non-adherent bacteria then incubated 20 min at 37°C in MEM supplemented with 4 μg/ml lysostaphin. Cells were then washed twice with MEM and placed in 20 ml MEM containing 100 μg/ml gentamicin (defines time = 0). At 2 and 6 hours post-infection, cells were washed twice with PBS, fixed in acetone/EtOH (1:1) and processed for RNA extraction.

### Cell viability test

Cells (1 × 10^6^) were seeded in triplicate into culture plates 24 h prior to treatment. Bacterial infections were performed as described for the standard lysostaphin/gentamycin protection assay. Control experiments using anti-CD95 ligation (1 μg/ml) or etoposide (100 μM final concentration) were included for cytotoxicity measurements with trypan blue. At 24 hours post-internalization, cells were washed and the viability of monolayers determined using Trypan blue dye exclusion. Viability was evaluated and expressed as a percentage of the control condition plate. In some cases, we examined combined culture supernatants and monolayers to detect the presence of non-viable detached cells. No difference was noted from viability measurements of monolayers alone.

### Mock infection and cytochalasin D experiments

Fixation and RNA extractions were identical for every condition as described above. Cells were pretreated 30 minutes prior infection with 1 μg/ml cytochalasin D (Sigma) and also 60 minutes post-infection. Culture supernatants were collected and non-adherent bacterial cells were harvested, washed twice with PBS and fixed. Cell surface-attached bacteria were detached from A549 monolayer by rapid trypsin incubation, harvested, washed, and fixed. For mock infection, bacterial suspensions were prepared in IM in the absence of A549 cells.

### RNA extraction and purification

For all experiments, material (host cells or free bacteria) were lysed in the presence of ice cold acetone:ethanol (1:1) for 4 minutes. Cell monolayers were maintained on ice, then scraped and centrifuged briefly at 4°C. Cellular debris and bacterial pellets were suspended in ice cold RLT buffer (Qiagen) and centrifuged, then washed four times in ice cold TE (10 mM Tris/HCl, 1 mM EDTA pH 8.0). Fixed and washed bacteria were lysed in TE containing 200 μg/ml lysostaphin for 15 minutes at 37°C as previously described [87,88]. RNA was purified using RNeasy columns (Qiagen) following the manufacturer's instructions. For each infection experiment, RNA from 6 wells was pooled. Purified total RNA extracts were treated with MicrobENRICH (Ambion) following the manufacturer's instructions. RNA quality and yields were monitored before and after enrichment using a 2100 BioAnalyzer (Agilent) on PicoLab chip kit (Agilent).

### cDNA synthesis, amplification, and fluorescent labeling

Total RNA (300 ng per condition) was converted into cDNA by Superscript II (Invitrogen) and amplified following a previously described method [89] yielding limited bias and robust amplification [90]. Briefly, 20 ng of ds cDNA was amplified using the Genomiphi kit (Amersham Biosciences) according to the manufacturer's recommendations, then labeled with Cy3-dCTP (Perkin Elmer) using BioPrime DNA Labeling System (Invitrogen). Cy5-dCTP (Perkin Elmer) labeled genomic DNA (0.5 mg) pooled from *S. aureus *strains N315, Mu50, COL, and MW2 was used as reference for the normalization process, according to previous work [53,91].

### *S. aureus *microarray and hybridization conditions

The *S. aureus *60-mer oligonucleotide microarray contains 8455 oligonucleotides probes covering > 99% of genes of 4 sequenced *S. aureus *strains: N315, Mu50, MW2 and COL [53]. Labeled cDNA (signal channel, Cy3) and genomic DNA (control channel, Cy5) were mixed in 250 μL Agilent hybridization buffer and hybridized at 60°C for 17 hours. Slides were washed, dried under nitrogen, and scanned at 100% photomultiplier tube power for both wavelengths (Agilent). Microarrays for all experimental time-points were performed in triplicate.

### Data analysis

Local background subtracted signals were calculated from three independent experiments using Feature Extraction software (Agilent version A6.1.1). To ensure spot quality, features and their respective background which were not uniform in pixel fluorescence intensity distribution in both channels were flagged (non-uniformity outlier flagging algorithm). Spots showing a genomic DNA reference signal not significantly different from corresponding background were also flagged (two-tailed Student's *t*-test feature versus background with *p *< 0.05). Raw data were imported and analyzed using GeneSpring 7.2 (Agilent). Data were normalized using both per spot (signal channel divided by the corresponding control channel and generation of log_10 _ratio) and per chip (to the 50^th ^percentile). Flagged spots of low quality were excluded from further analysis. Replicates from the same test condition were grouped and the mean calculated. Significance of normalized data was determined using Welch's approximate *t*-test adjusting individual *P *values with the Benjamini-Hochberg correction for false discovery rate multiple test. Genes showing significant change in expression between two conditions and fold change ≥ 3 were defined as differentially expressed. Fold changes for the complete dataset is available online as additional file 2.

The analysis of differential gene expression patterns in nine possible combinations when comparing 0, 2, and 6 h was performed using the Welch conditional parametric test and false discovery rate correction (variances assumed unequal, *p *< 0.05). Genes showing fold changes > 1.5 are shown.

### Quantitative PCR

To confirm microarray results, the expression levels of *hla*, *sigB*, *agrA*, *sodA, sarS and lytM *genes were determined by real-time PCR analysis. Genomiphi-amplified material (1 ng) from independent experiments was assembled with specific primers (Invitrogen) and fluorescent probes (Table 2) designed using the PrimerExpress software (Applied Biosystems) in specific enzymatic kit mixtures (Eurogentec). PCR reactions were performed in a final volume of 20 μl in a SDS7700 (Applied Biosystems). Relative expression levels were determined by comparing cycle threshold (Ct) of each gene to the Ct value of the *hu *gene for the same cDNA preparation [92].

**Table 2:** List and characteristics of oligonucleotides used in the qPCR control experiments

**Primer or Probe name**	**Sequence (5'→3')**	**Length**	^A^**5'Dye**	**ORF number in N315**
***HU***				**SA1305**
F*_HU_*34	CAAAGAGAAAACATGGTTACCATTATTAA	29		
R*_HU*_135	CTCAAGCACCTCATAAGGATTATCAG	26		
P*_HU*_83^A^	AAAAGCCTATGGAAATTGCCCTCGCA	26	FAM	
				
***agrA***				**SA1844**
F*_agrA_*34	CAAAGAGAAAACATGGTTACCATTATTAA	29		
R*_agrA*_135	CTCAAGCACCTCATAAGGATTATCAG	26		
P*_agrA*_83^A^	AAAAGCCTATGGAAATTGCCCTCGCA	26	FAM	
				
***sarS***				**SA0108**
F*_sarS_553*	CACCATAAATACCCTCAAACTGTTAGAG	28		
R*_sarS_638*	TCATCTTCAGTTGAGCGTTCTTTT	24		
P*_sarS_596*^B^	AAAAGCAAGGCTATCTAA	18	FAM	
				
***lytM***				**SA0265**
F*_lytM_32*	CGATGGGCTTCGCTACATTT	20		
R*_lytM_105*	ATGTGCTTGTTGGGTGTTTGTC	22		
P*_lytM_56*^A^	TGGCGCATCAAGCAGATGCAGC	22	FAM	
				
***sodA***				**SA1382**
F*_sodA_168*	TGTTGCTAATTTAGACAGTGTACCAGCTA	29		
R*_sodA_275*	TCCCAGAATAATGAATGGTTTAAATG	26		
P*_sodA_198*^A^	CATCCAAACTGCTGTACGTAATAATGGCGG	30	FAM	
				
***sirA***				**SA0111**
F*_sirA_151*	GGGAAACCAAAGCGTGTTGT	20		
R*_sirA_246*	TGTCCATGATTCTACAGCACCTACA	25		
P*_sirA_198*^A^	ATATCAAGGTGCCACTGACGTCGCTGT	27	FAM	

^A ^Taqman probes with a 3'-TAMRA quencher (Eurogentec, Seraing, Belgium).

^B ^Minor groove binder probe with non-fluorescent quencher bound to the 3'end (Applied Biosystems). All experiments used concentrations of 200 nM and 100 nM for primers and probes, respectively. Primers and probe for *hla *quantification have been published previously [87,88].

## Authors' contributions

CG contributed to the design of the study, performed the totality of microarray experiments including RNA extraction, amplification and data analysis, PF and JS conceived the study, contributed to experimental design and analysis and coordinated final writing, CT contributed to the real-time PCR validation experiments, AH and YC contributed to the design of the microarray and developed the required bioinformatics, SL was involved in microarray interpretation, SC was involved in the design and interpretation of results obtained in the infection model, AR was involved in microarray interpretation and contributed to MS elaboration, DL and PV contributed to manuscript elaboration, WK was involved in the design, experiments and analysis related to the infection model including apoptosis experiments and in the elaboration of the manuscript.

## Supplementary Material

Additional file 1 **Reproducibility of RNA amplification**. cDNA was generated in 2 separate reactions from the same RNA pool, amplified independently and hybridized in 2 different arrays. Labeled cDNA from the starting material was used as control. Dye normalized intensities of amplified material are plotted in both axis. Click here for file

Additional file 2 **Fold changes at 2 h and 6 h of all genes compared to control bacteria**. Values represent mean of fold change from 3 or 4 independent biological replicates. * Some genes are classified in two different COG categories and appear on separate lines in the table. Click here for file

Additional file 3 **Genes differentially expressed grouped by function**. Values represent mean of fold change from 3 or 4 independent biological replicates. * Some genes are classified in two different COG categories and appear on separate lines in the table. Click here for file
