# Supplementary figures and images for: A global view of Staphylococcus aureus whole genome expression upon internalization in human epithelial cells
Source: BMC Genomics. 2007 Jun 14;8:171. doi: 10.1186/1471-2164-8-171 (PMC1924023; doi:10.1186/1471-2164-8-171)

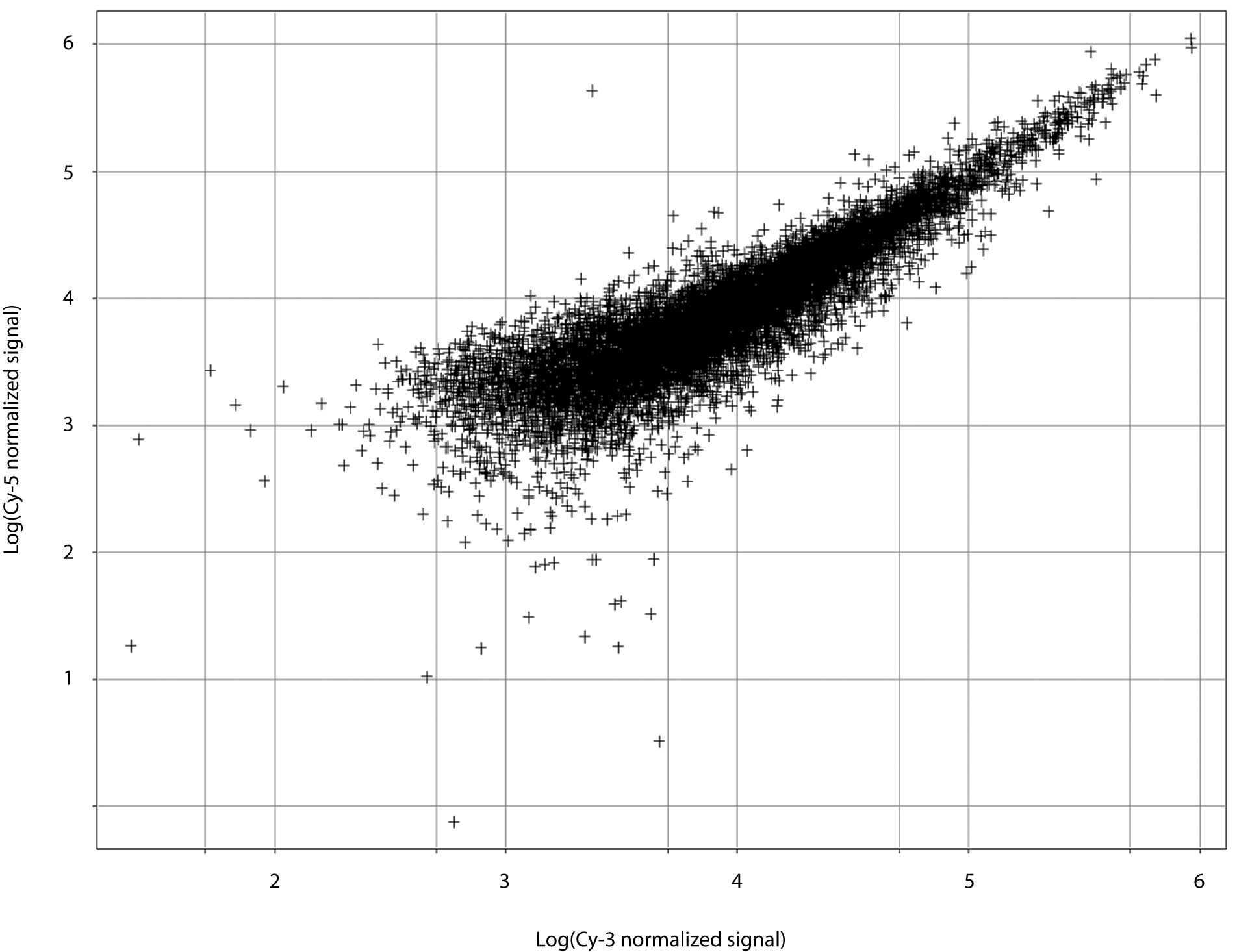

Supplement: Additional file 1 — Reproducibility of RNA amplification. cDNA was generated in 2 separate reactions from the same RNA pool, amplified independently and hybridized in 2 different arrays. Labeled cDNA from the starting material was used as control. Dye normalized intensities of amplified material are plotted in both axis. [file 1471-2164-8-171-S1.tiff]
