# Supplementary material for: A global view of Staphylococcus aureus whole genome expression upon internalization in human epithelial cells
Source: BMC Genomics. 2007 Jun 14;8:171. doi: 10.1186/1471-2164-8-171 (PMC1924023; doi:10.1186/1471-2164-8-171)
Supplement: Additional file 2 — Fold changes at 2 h and 6 h of all genes compared to control bacteria. Values represent mean of fold change from 3 or 4 independent biological replicates. * Some genes are classified in two different COG categories and appear on separate lines in the table. [file 1471-2164-8-171-S2.pdf]

| | Common | Organism | 2h fold change | 6h fold change | Protein name | GO* | class | Annot |
|---|---|---|---|---|---|---|---|---|
| SA0033 | aadD | N315 | 3.69 | | kanamycin nucleotidyltransferase | | | - |
| SA1519 | aapA | N315 | 0.17 | 0.17 | Dserine/Dalanine/glycine transporter | COG1113E | E | Amino acid transport and metabolism |
| SA1522 | accA | N315 | 0.09 | 0.11 | acetylCoA carboxylase carboxyl transferase subunit alpha | COG0825I | I | Lipid transport and metabolism |
| SA1357 | accC | N315 | 0.04 | 0.09 | acetylCoA carboxylase accC | COG0439I | I | Lipid transport and metabolism |
| SA0562 | adh1 | N315 | 0.14 | 0.14 | alcohol dehydrogenase I | COG1064R | R | General function prediction only |
| SA2027 | adk | N315 | 0.01 | 0.15 | adenylate kinase | COG0563F | F | Nucleotide transport and metabolism |
| SA1844 | agrA | N315 | 0.10 | 0.12 | AgrA | COG3279KT | t | Signal transduction mechanisms |
| SA1843 | agrC | N315 | 0.01 | 0.02 | AgrC | COG2972T | T | Signal transduction mechanisms |
| SA0366 | ahpC | N315 | 0.11 | 0.11 | alkyl hydroperoxide reductase subunit C | COG0450O | O | Posttranslational modification, protein turnover, chaperones |
| SA0365 | ahpF | N315 | 0.11 | 0.30 | alkyl hydroperoxide reductase subunit F | COG3634O | O | Posttranslational modification, protein turnover, chaperones |
| SA1531 | ald | N315 | 3.07 | | alanine dehydrogenase | COG0686E | E | Amino acid transport and metabolism |
| SA0162 | aldA | N315 | 3.70 | | aldehyde dehydrogenase homologue | COG1012C | C | Energy production and conversion |
| SA1874 | alr | N315 | 0.21 | 0.23 | alanine racemase | COG0787M | M | Cell wall/membrane/envelope biogenesis |
| SA1190 | alsT | N315 | 0.18 | | amino acid carrier protein | COG1115E | E | Amino acid transport and metabolism |
| SA1695 | ampS | N315 | 0.17 | 0.18 | aminopeptidase ampS | COG2309E | E | Amino acid transport and metabolism |
| SA0822 | argG | N315 | 0.30 | | argininosuccinate synthase | COG0137E | E | Amino acid transport and metabolism |
| SA0564 | argS | N315 | 0.19 | 0.17 | arginyltRNA synthetase | COG0018J | J | Translation, ribosomal structure and biogenesis |
| SA1246 | arlS | N315 | 0.33 | | putative protein histidine kinase ArlS | COG0642T | T | Signal transduction mechanisms |
| SA1297 | aroA | N315 | 0.08 | 0.17 | 3phosphoshikimate 1carboxyvinyltransferase | COG0128E | E | Amino acid transport and metabolism |
| SA1298 | aroB | N315 | 0.27 | | 3dehydroquinate synthase | COG0337E | E | Amino acid transport and metabolism |
| SA1424 | aroE | N315 | 0.03 | 0.04 | shikimate dehydrogenease | COG0169E | E | Amino acid transport and metabolism |
| SA1226 | asd | N315 | 0.13 | 0.23 | aspartate semialdehyde dehydrogenase | COG0136E | E | Amino acid transport and metabolism |
| SA1984 | asp23 | N315 | 0.04 | 0.06 | alkaline shock protein 23, ASP23 | | | - |
| SA1456 | aspS | N315 | 0.10 | 0.06 | aspartyltRNA synthetase | COG0173J | J | Translation, ribosomal structure and biogenesis |
| SA0905 | atl | N315 | 0.04 | 0.20 | autolysin | COG4193G,COG5632M | G | Carbohydrate transport and metabolism |
| SA1907 | atpA | N315 | 0.08 | 0.25 | ATP synthase alpha chain | COG0056C | C | Energy production and conversion |
| SA1911 | atpB | N315 | 0.01 | 0.03 | ATP synthase A chain | | | - |
| SA1904 | atpC | N315 | 0.19 | 0.24 | FoF1ATP synthase epsilon subunit | | | - |
| SA1905 | atpD | N315 | 0.02 | 0.09 | ATP synthase beta chain | COG0055C | C | Energy production and conversion |
| SA1910 | atpE | N315 | 0.01 | 0.10 | ATP synthase C chain | COG0636C | C | Energy production and conversion |
| SA1909 | atpF | N315 | 0.01 | 0.12 | ATP synthase B chain | COG0711C | C | Energy production and conversion |
| SA1906 | atpG | N315 | 0.05 | 0.15 | ATP synthase gamma chain | COG0224C | C | Energy production and conversion |
| SA1908 | atpH | N315 | 0.08 | 0.20 | ATP synthase delta chain | COG0712C | C | Energy production and conversion |
| SA2430 | aur | N315 | 3.91 | | zinc metalloproteinase aureolysin | COG3227E | E | Amino acid transport and metabolism |
| SA2405 | betA | N315 | 0.24 | 0.30 | choline dehydrogenase | COG2303E | E | Amino acid transport and metabolism |
| SA1396 | bex | N315 | 0.16 | 0.18 | GTPbinding protain Era homolog | COG1159R | R | General function prediction only |
| SA1347 | bfmBAB | N315 | 0.23 | 0.28 | branchedchain alphaketo acid dehydrogenase E1 | COG0022C | C | Energy production and conversion |
| SA2213 | bioB | N315 | 0.08 | 0.14 | biotin synthase | COG0502H | H | Coenzyme transport and metabolism |
| SA1346 | bmfBB | N315 | 0.31 | | branchedchain alphaketo acid dehydrogenase E2 | COG0508C | C | Energy production and conversion |
| MW1766 | bsaA1 | MW2 | 3.51 | | gallidermin precursor [Genomic island nu Sa beta2] | | | - |
| MW1764 | bsaB | MW2 | 3.89 | 3.83 | lantibiotic epidermin biosynthesis protein EpiB [Genomic island nu Sa beta2] | | | - |
| MW0136 | cap8M | MW2 | 0.07 | 0.23 | capsular polysaccharide synthesis enzyme Cap8M | COG2148M | M | Cell wall/membrane/envelope biogenesis |
| SA0149 | capF | N315 | 0.23 | 0.25 | capsular polysaccharide synthesis enzyme Cap5F | COG1898M,COG0451MG | g | Carbohydrate transport and metabolism |
| SA0150 | capG | N315 | 0.15 | 0.24 | capsular polysaccharide synthesis enzyme Cap5G | COG0381M | M | Cell wall/membrane/envelope biogenesis |
| SA0152 | capI | N315 | 3.16 | | capsular polysaccharide synthesis enzyme Cap5I | COG0438M | M | Cell wall/membrane/envelope biogenesis |
| SA0157 | capN | N315 | 0.18 | 0.32 | capsular polysaccharide synthesis enzyme Cap5N | COG0451MG | g | Carbohydrate transport and metabolism |
| SA1557 | ccpA | N315 | 0.29 | 0.29 | catabolite control protein A | COG1609K | K | Transcription |
| SA1397 | cdd | N315 | 0.15 | 0.19 | cytidine deaminase | COG0295F | F | Nucleotide transport and metabolism |
| SA0831 | cdr | N315 | 0.31 | 0.33 | coenzyme A disulfide reductase | COG0446R | R | General function prediction only |
| SA1065 | cfxE | N315 | 0.28 | 0.31 | ribulose5phosphate 3epimerase homolog | COG0036G | G | Carbohydrate transport and metabolism |
| SA1184 | citB | N315 | 0.01 | 0.01 | aconitate hydratase | COG1048C | C | Energy production and conversion |
| SA1517 | citC | N315 | 0.16 | 0.19 | isocitrate dehyrogenase | COG0538C | C | Energy production and conversion |
| SA1518 | citZ | N315 | 0.18 | 0.17 | citrate synthase II | COG0372C | C | Energy production and conversion |
| SA2423 | clfB | N315 | 0.17 | 0.19 | Clumping factor B | | | - |
| SA0483 | clpC | N315 | 0.23 | | endopeptidase | COG0542O | O | Posttranslational modification, protein turnover, chaperones |
| SA1498 | clpX | N315 | 0.29 | 0.33 | protease ClpX | COG1219O | O | Posttranslational modification, protein turnover, chaperones |
| SA1097 | clpY | N315 | 0.14 | 0.28 | heat shock protein HslU | COG1220O | O | Posttranslational modification, protein turnover, chaperones |
| SA2349 | crtM | N315 | 0.22 | 0.27 | squalene synthase | COG1562I | I | Lipid transport and metabolism |
| SA2494 | cspB | N315 | 5.24 | | cold shock protein cspB | | | - |
| SA0965 | ctaB | N315 | 0.16 | 0.16 | cytochrome caa3 oxidase (assembly factor) homolog | COG0109O | O | Posttranslational modification, protein turnover, chaperones |
| SA1929 | ctrA | N315 | 0.10 | 0.11 | CTP synthase | COG0504F | F | Nucleotide transport and metabolism |
| SA0480 | ctsR | N315 | 0.16 | | transcription repressor of class III stress genes homologue | | | - |
| SA0487 | cysE | N315 | 0.32 | | Serine acetyltransferase homologue | COG1045E | E | Amino acid transport and metabolism |
| SA0471 | cysK | N315 | 0.19 | 0.12 | cysteine synthase (oacetylserine sulfhydrylase) homologue | COG0031E | E | Amino acid transport and metabolism |
| SA0418 | cysM | N315 | 3.39 | 3.69 | cysteine synthase homologue | COG0031E | E | Amino acid transport and metabolism |
| SA0488 | cysS | N315 | 0.09 | 0.13 | cysteinyltRNA synthetase | COG0215J | J | Translation, ribosomal structure and biogenesis |
| SA1948 | czrB | N315 | | 0.33 | cationefflux system membrane protein homolog | COG1230P | P | Inorganic ion transport and metabolism |
| SA1227 | dapA | N315 | 0.08 | 0.14 | dihydrodipicolinate synthase | COG0329EM | e | Amino acid transport and metabolism |
| MW1284 | dapB | MW2 | 0.31 | 0.29 | dihydrodipicolinate reductase | COG0289E | E | Amino acid transport and metabolism |
| SA1887 | ddlA | N315 | 0.33 | | DalanineDalanine ligase | COG1181M | M | Cell wall/membrane/envelope biogenesis |
| SA1259 | dfrA | N315 | 0.03 | 0.20 | dihydrofolate reductase | COG0262H | H | Coenzyme transport and metabolism |
| SA1164 | dhoM | N315 | 0.28 | | homoserine dehydrogenase | COG0460E | E | Amino acid transport and metabolism |
| SA1288 | dinG | N315 | 0.29 | 0.25 | probable ATPdependent DNA helicase dinG | COG0847L,COG1199KL | L | Replication, recombination and repair |
| SA1027 | div1b | N315 | 0.05 | 0.11 | cell division protein, FtsQ homolog | COG1589M | M | Cell wall/membrane/envelope biogenesis |
| SA0793 | dltA | N315 | 0.07 | 0.17 | DalanineDalanyl carrier protein ligase | COG1020Q | Q | Secondary metabolites biosynthesis, transport and catabolism |
| SA0794 | dltB | N315 | 0.04 | 0.10 | DltB membrane protein | COG1696M | M | Cell wall/membrane/envelope biogenesis |
| SA0795 | dltC | N315 | 0.06 | 0.10 | Dalanyl carrier protein | COG0236IQ | i | Lipid transport and metabolism |
| SA0796 | dltD | N315 | 0.16 | 0.18 | poly Dalanine transfer protein | COG3966M | M | Cell wall/membrane/envelope biogenesis |
| SA1508 | dnaB | N315 | 0.04 | 0.05 | chromosome replication initiation/membrane attachment protei | COG3611L | L | Replication, recombination and repair |
| SA0015 | dnaC | N315 | 0.23 | 0.26 | replicative DNA helicase | COG0305L | L | Replication, recombination and repair |
| SA1525 | dnaE | N315 | 0.14 | 0.15 | DNA polymerase III, alpha chain | COG0587L | L | Replication, recombination and repair |
| SA1391 | dnaG | N315 | 0.15 | 0.25 | DNA primase | COG0358L | L | Replication, recombination and repair |
| SA1507 | dnaI | N315 | 0.07 | 0.07 | primosomal protein | COG1484L | L | Replication, recombination and repair |
| SA1408 | dnaJ | N315 | 0.31 | | DnaJ protein | COG0484O | O | Posttranslational modification, protein turnover, chaperones |
| SA1409 | dnaK | N315 | 0.22 | | DnaK protein | COG0443O | O | Posttranslational modification, protein turnover, chaperones |
| SA0002 | dnaN | N315 | 0.12 | 0.14 | DNA polymerase III, beta chain | COG0592L | L | Replication, recombination and repair |
| SA1875 | dpj | N315 | 0.14 | 0.22 | holoACP synthase | COG0736I | I | Lipid transport and metabolism |
| SA1941 | dps | N315 | 3.40 | 3.66 | general stress protein 20U | COG0783P | P | Inorganic ion transport and metabolism |
| MW0113 | drm | MW2 | | 0.18 | phosphopentomutase | COG1015G | G | Carbohydrate transport and metabolism |
| SA0134 | drm | N315 | 0.32 | | phosphopentomutase | COG1015G | G | Carbohydrate transport and metabolism |
| SA2480 | drp35 | N315 | 3.25 | 3.47 | Drp35 | COG3386G | G | Carbohydrate transport and metabolism |
| SA1267 | ebhA | N315 | 3.23 | 3.41 | streptococcal adhesin emb | COG0419L | L | Replication, recombination and repair |
| SA0731 | eno | N315 | 0.04 | 0.13 | enolase | COG0148G | G | Carbohydrate transport and metabolism |
| SA0843 | fab | N315 | 0.30 | 0.31 | 3oxoacyl synthase | COG0304IQ | i | Lipid transport and metabolism |
| SA1073 | fabD | N315 | 0.18 | 0.27 | malonyl CoAacyl carrier protein transacylase | COG0331I | I | Lipid transport and metabolism |
| SA1074 | fabG | N315 | 0.31 | 0.33 | 3oxoacyl reductase | COG1028IQR | r | General function prediction only |
| SA0842 | FabH | N315 | 0.10 | 0.08 | FabH | COG0332I | I | Lipid transport and metabolism |
| SA0869 | fabI | N315 | 0.13 | 0.18 | trans2enoylACP reductase | COG0623I | I | Lipid transport and metabolism |
| SA1927 | fbaA | N315 | 0.14 | 0.15 | fructosebisphosphate aldolase | COG0191G | G | Carbohydrate transport and metabolism |
| SA1206 | femA | N315 | 0.33 | 0.28 | factor essential for expression of methicillin resistance | COG2348V | V | Defense mechanisms |
| SA1207 | femB | N315 | 0.14 | 0.10 | FemB protein | COG2348V | V | Defense mechanisms |
| SA1080 | ffh | N315 | 0.17 | 0.15 | signal recognition particle homolog | COG0541U | U | Intracellular trafficking, secretion, and vesicular transport |
| SA1553 | fhs | N315 | 0.14 | 0.19 | formyltetrahydrofolate synthetase | COG2759F | F | Nucleotide transport and metabolism |
| SA0602 | fhuA | N315 | 0.28 | | ferrichrome transport ATPbinding protein | COG1120PH | h | Coenzyme transport and metabolism |
| SA0603 | fhuB | N315 | 0.16 | 0.24 | ferrichrome transport permease | COG0609P | P | Inorganic ion transport and metabolism |
| SA1193 | fmtC | N315 | 0.07 | 0.16 | oxacillin resistancerelated FmtC protein | COG0392S,COG2898S | S | Function unknown |
| SA2291 | fnb | N315 | 3.23 | | fibronectinbinding protein homolog | | | - |
| SA2290 | fnbB | N315 | | 3.83 | fibronectinbinding protein homolog | | | - |
| SA1487 | folC | N315 | 0.12 | 0.33 | folylpolyglutamate synthase | COG0285H | H | Coenzyme transport and metabolism |
| SA0915 | folD | N315 | 0.05 | 0.08 | FolD bifunctional protein | COG0190H | H | Coenzyme transport and metabolism |
| SA0472 | folP | N315 | 3.52 | | dihydropteroate synthase chain A synthetase | COG0294H | H | Coenzyme transport and metabolism |
| SA2124 | fosB | N315 | | 3.45 | fosfomycin resistance protein fofB | COG0346E | E | Amino acid transport and metabolism |
| SA1102 | frr | N315 | 0.08 | 0.23 | ribosome recycling factor | | | - |
| SA0655 | fruA | N315 | 3.47 | 3.65 | fructose specific permease | COG1762GT,COG1299G | g | Carbohydrate transport and metabolism |
| SA0469 | ftsH | N315 | 0.11 | 0.16 | celldivision protein | COG0465O | O | Posttranslational modification, protein turnover, chaperones |
| SA1029 | ftsZ | N315 | 0.05 | 0.07 | cell division protein | COG0206D | D | Cell cycle control, cell division, chromosome partitioning |
| SA0505 | fus | N315 | 0.07 | 0.25 | translational elongation factor G | COG0480J | J | Translation, ribosomal structure and biogenesis |
| SA0727 | gap | N315 | 0.10 | 0.27 | glyceraldehyde3phosphate dehydrogenase | COG0057G | G | Carbohydrate transport and metabolism |
| SA0726 | gapR | N315 | 0.31 | | glycolytic operon regulator | COG2390K | K | Transcription |
| SA0309 | geh | N315 | 0.04 | 0.07 | glycerol ester hydrolase | COG1075R | R | General function prediction only |
| SA1302 | gerCC | N315 | 0.32 | 0.22 | heptaprenyl diphosphate syntase component II | COG0142H | H | Coenzyme transport and metabolism |
| SA1094 | gid | N315 | 0.04 | 0.07 | glucoseinhibited division protein gid | COG1206J | J | Translation, ribosomal structure and biogenesis |
| SA2500 | gidA | N315 | 0.08 | 0.29 | glucose inhibited division protein A | COG0445D | D | Cell cycle control, cell division, chromosome partitioning |
| SA2499 | gidB | N315 | 0.12 | 0.32 | glucose inhibited division protein B | COG0357M | M | Cell wall/membrane/envelope biogenesis |
| SA0183 | glcA | N315 | 0.15 | 0.14 | PTS enzyme II (EC 2.7.1.69), glucosespecific, factor IIA homol | COG1263G | G | Carbohydrate transport and metabolism |
| SA1377 | glcK | N315 | 0.19 | 0.16 | glucokinase | COG1940KG | g | Carbohydrate transport and metabolism |
| SA1965 | glmM(femD) | N315 | 0.32 | 0.28 | phosphoglucosaminemutase | COG1109G | G | Carbohydrate transport and metabolism |
| SA1150 | glnA | N315 | 0.22 | | glutamineammonia ligase | | | - |
| SA1140 | glpF | N315 | 3.37 | 3.16 | glycerol uptake facilitator | | | - |
| SA1139 | glpP | N315 | 0.15 | 0.12 | glycerol uptake operon antiterminator regulatory protein | COG1954K | K | Transcription |
| SA0820 | glpQ | N315 | 3.16 | | glycerophosphoryl diester phosphodiesterase | COG0584C | C | Energy production and conversion |
| SA0325 | glpT | N315 | 4.74 | 4.10 | glycerol3phosphate transporter | COG2271G | G | Carbohydrate transport and metabolism |
| SA0486 | gltX | N315 | 0.18 | 0.18 | glutamyltRNA synthetase | COG0008J | J | Translation, ribosomal structure and biogenesis |
| SA1915 | glyA | N315 | 0.03 | 0.06 | serine hydroxymethyl transferase | COG0112E | E | Amino acid transport and metabolism |
| SA1342 | gnd | N315 | 0.09 | 0.09 | phosphogluconate dehydrogenase | COG0362G | G | Carbohydrate transport and metabolism |
| SA2294 | gntK | N315 | 3.51 | | gluconokinase | COG1070G | G | Carbohydrate transport and metabolism |
| SA2293 | gntP | N315 | 3.13 | | gluconate permease | COG2610GE | e | Amino acid transport and metabolism |
| SA1306 | gpsA | N315 | 0.05 | 0.08 | glycerol3phosphate dehydrogenase | COG0240C | C | Energy production and conversion |
| SA1836 | groEL | N315 | 0.20 | 0.24 | GroEL protein | COG0459O | O | Posttranslational modification, protein turnover, chaperones |
| SA0376 | guaA | N315 | 0.11 | 0.12 | GMP synthase | COG0518F,COG0519F | F | Nucleotide transport and metabolism |
| SA0375 | guaB | N315 | 0.06 | 0.09 | inositolmonophosphate dehydrogenase | COG0517R,COG0516F | R | General function prediction only |
| SA0819 | gudB | N315 | | 0.33 | NADspecific glutamate dehydrogenase | COG0334E | E | Amino acid transport and metabolism |
| SA0006 | gyrA | N315 | 0.13 | 0.11 | DNA gyrase subunit A | COG0188L | L | Replication, recombination and repair |
| SA0005 | gyrB | N315 | 0.13 | 0.12 | DNA gyrase subunit B | COG0187L | L | Replication, recombination and repair |
| SA1496 | hemA | N315 | 0.15 | 0.28 | glutamyltRNA reductase | COG0373H | H | Coenzyme transport and metabolism |
| SA1492 | hemB | N315 | 0.06 | 0.14 | deltaaminolevulinic acid dehydratase | | | - |
| SA1494 | hemC | N315 | 0.20 | | porphobilinogen deaminase | | | - |

| | | | | | | | | |
|---|---|---|---|---|---|---|---|---|
| **SA1493** | *hemD* | N315 | 0.24 | | uroporphyrinogen III synthase | **COG1587H** | **H** | Coenzyme transport and metabolism |
| **SA1652** | *hemE* | N315 | 0.14 | | uroporphyrinogen decarboxylase | **COG0407H** | **H** | Coenzyme transport and metabolism |
| **SA1651** | *hemH* | N315 | 0.32 | | ferrochelatase homolog | | | - |
| **SA1491** | *hemL* | N315 | 0.03 | 0.03 | glutamate1semialdehyde 2,1aminomutase | **COG0001H** | **H** | Coenzyme transport and metabolism |
| **SA1495** | *hemX* | N315 | 0.05 | 0.26 | hemA concentration negative effector hemX | **COG0755O** | **O** | Posttranslational modification, protein turnover, chaperones |
| **SA1650** | *hemY* | N315 | 0.15 | 0.23 | protoporphyrinogen oxidase | **COG1232H** | **H** | Coenzyme transport and metabolism |
| **MW1940** | *hlb* | MW2 | 3.08 | | truncated betahemolysin | | | - |
| **SAS065** | *hld* | N315 | 3.05 | | deltahemolysin | | | - |
| **SA2209** | *hlgB* | N315 | | 3.12 | gammahemolysin component B | | | - |
| **SA0055** | HP | N315 | 4.18 | 4.74 | Hypothetical protein | | | - |
| **SA0213** | HP | N315 | 9.31 | 3.91 | Hypothetical protein | | | - |
| **SA0301** | HP | N315 | 7.24 | 3.14 | Hypothetical protein | | | - |
| **SA0360** | HP | N315 | 3.34 | 3.55 | Hypothetical protein | | | - |
| **SA0554** | HP | N315 | 5.02 | 4.31 | Hypothetical protein | | | - |
| **SA0630** | HP | N315 | 4.04 | 3.91 | Hypothetical protein | | | - |
| **SA0632** | HP | N315 | 4.29 | 3.70 | Hypothetical protein | | | - |
| **SA0906** | HP | N315 | 3.96 | 3.25 | Hypothetical protein | | | - |
| **SA1744** | HP | N315 | 3.64 | 4.61 | Hypothetical protein | | | - |
| **SA2478** | HP | N315 | 3.82 | 3.55 | Hypothetical protein | | | - |
| **SA2479** | HP | N315 | 18.60 | 9.90 | Hypothetical protein | | | - |
| **SA0024** | HP | N315 | 3.83 | | Hypothetical protein | | | - |
| **SA0025** | HP | N315 | 12.89 | | Hypothetical protein | | | - |
| **SA0030** | HP | N315 | 4.05 | | Hypothetical protein | | | - |
| **SA0031** | HP | N315 | 4.42 | | Hypothetical protein | | | - |
| **SA0072** | HP | N315 | 4.95 | | Hypothetical protein | | | - |
| **SA0074** | HP | N315 | 50.25 | | Hypothetical protein | | | - |
| **SA0076** | HP | N315 | 8.15 | | Hypothetical protein | | | - |
| **SA0080** | HP | N315 | 3.21 | | Hypothetical protein | | | - |
| **SA0096** | HP | N315 | 3.19 | | Hypothetical protein | | | - |
| **SA0101** | HP | N315 | 5.97 | | Hypothetical protein | | | - |
| **SA0103** | HP | N315 | 3.10 | | Hypothetical protein | | | - |
| **SA0105** | HP | N315 | 3.15 | | Hypothetical protein | | | - |
| **SA0188** | HP | N315 | 4.11 | | Hypothetical protein | | | - |
| **SA0203** | HP | N315 | 4.45 | | Hypothetical protein | | | - |
| **SA0269** | HP | N315 | 3.09 | | Hypothetical protein | | | - |
| **SA0292** | HP | N315 | 6.71 | | Hypothetical protein | | | - |
| **SA0355** | HP | N315 | 4.02 | | Hypothetical protein | | | - |
| **SA0358** | HP | N315 | 3.70 | | Hypothetical protein | | | - |
| **SA0377** | HP | N315 | 3.12 | | Hypothetical protein | | | - |
| **SA0378** | HP | N315 | 5.20 | | Hypothetical protein | | | - |
| **SA0394** | HP | N315 | 3.43 | | Hypothetical protein | | | - |
| **SA0399** | HP | N315 | 3.92 | | Hypothetical protein | | | - |
| **SA0400** | HP | N315 | 4.19 | | Hypothetical protein | | | - |
| **SA0402** | HP | N315 | 3.21 | | Hypothetical protein | | | - |
| **SA0403** | HP | N315 | 4.25 | | Hypothetical protein | | | - |
| **SA0532** | HP | N315 | 4.17 | | Hypothetical protein | | | - |
| **SA0575** | HP | N315 | 3.70 | | Hypothetical protein | | | - |
| **SA0651** | HP | N315 | 4.93 | | Hypothetical protein | | | - |
| **SA0751** | HP | N315 | 4.49 | | Hypothetical protein | | | - |
| **SA0752** | HP | N315 | 26.47 | | Hypothetical protein | | | - |
| **SA0798** | HP | N315 | 5.77 | | Hypothetical protein | | | - |
| **SA0844** | HP | N315 | 5.73 | | Hypothetical protein | | | - |
| **SA0883** | HP | N315 | 4.05 | | Hypothetical protein | | | - |
| **SA0889** | HP | N315 | 3.09 | | Hypothetical protein | | | - |
| **SA0930** | HP | N315 | 4.71 | | Hypothetical protein | | | - |
| **SA1002** | HP | N315 | 4.38 | | Hypothetical protein | | | - |
| **SA1179** | HP | N315 | 3.33 | | Hypothetical protein | | | - |
| **SA1208** | HP | N315 | 3.67 | | Hypothetical protein | | | - |
| **SA1284** | HP | N315 | 3.42 | | Hypothetical protein | | | - |
| **SA1320** | HP | N315 | 4.87 | | Hypothetical protein | | | - |
| **SA1437** | HP | N315 | 4.71 | | Hypothetical protein | | | - |
| **SA1477** | HP | N315 | 3.33 | | Hypothetical protein | | | - |
| **SA1567** | HP | N315 | 3.72 | | Hypothetical protein | | | - |
| **SA1600** | HP | N315 | 4.24 | | Hypothetical protein | | | - |
| **SA1610** | HP | N315 | 4.67 | | Hypothetical protein | | | - |
| **SA1616** | HP | N315 | 4.66 | | Hypothetical protein | | | - |
| **SA1619** | HP | N315 | 3.22 | | Hypothetical protein | | | - |
| **SA1621** | HP | N315 | 3.58 | | Hypothetical protein | | | - |
| **SA1670** | HP | N315 | 5.42 | | Hypothetical protein | | | - |
| **SA1706** | HP | N315 | 15.00 | | Hypothetical protein | | | - |
| **SA1767** | HP | N315 | 3.37 | | Hypothetical protein | | | - |
| **SA1778** | HP | N315 | 3.33 | | Hypothetical protein | | | - |
| **SA1782** | HP | N315 | 3.00 | | Hypothetical protein | | | - |
| **SA1786** | HP | N315 | 4.05 | | Hypothetical protein | | | - |
| **SA1788** | HP | N315 | 4.56 | | Hypothetical protein | | | - |
| **SA1797** | HP | N315 | 4.08 | | Hypothetical protein | | | - |
| **SA1802** | HP | N315 | 4.36 | | Hypothetical protein | | | - |
| **SA1809** | HP | N315 | 3.33 | | Hypothetical protein | | | - |
| **SA1821** | HP | N315 | 3.33 | | Hypothetical protein | | | - |
| **SA1825** | HP | N315 | 4.03 | | Hypothetical protein | | | - |
| **SA1826** | HP | N315 | 3.29 | | Hypothetical protein | | | - |
| **SA1832** | HP | N315 | 22.41 | | Hypothetical protein | | | - |
| **SA1928** | HP | N315 | 3.81 | | Hypothetical protein | | | - |
| **SA1971** | HP | N315 | 3.09 | | Hypothetical protein | | | - |
| **SA2015** | HP | N315 | 3.57 | | Hypothetical protein | | | - |
| **SA2107** | HP | N315 | 3.21 | | Hypothetical protein | | | - |
| **SA2249** | HP | N315 | 6.49 | | Hypothetical protein | | | - |
| **SA2263** | HP | N315 | 3.04 | | Hypothetical protein | | | - |
| **SA2267** | HP | N315 | 4.59 | | Hypothetical protein | | | - |
| **SA2292** | HP | N315 | 4.22 | | Hypothetical protein | | | - |
| **SA2321** | HP | N315 | 3.91 | | Hypothetical protein | | | - |
| **SA2338** | HP | N315 | 4.02 | | Hypothetical protein | | | - |
| **SA2398** | HP | N315 | 3.39 | | Hypothetical protein | | | - |
| **SA2432** | HP | N315 | 3.04 | | Hypothetical protein | | | - |
| **SA2444** | HP | N315 | 3.83 | | Hypothetical protein | | | - |
| **SA2485** | HP | N315 | 8.39 | | Hypothetical protein | | | - |
| **SA2488** | HP | N315 | 3.74 | | Hypothetical protein | | | - |
| **SAS002** | HP | N315 | 4.37 | | Hypothetical protein | | | - |
| **SAS003** | HP | N315 | 3.51 | | Hypothetical protein | | | - |
| **SAS009** | HP | N315 | 3.13 | | Hypothetical protein | | | - |
| **SAS013** | HP | N315 | 3.31 | | Hypothetical protein | | | - |
| **SAS015** | HP | N315 | 3.45 | | Hypothetical protein | | | - |
| **SAS017** | HP | N315 | 3.31 | | Hypothetical protein | | | - |
| **SAS019** | HP | N315 | 4.61 | | Hypothetical protein | | | - |
| **SAS024** | HP | N315 | 5.00 | | Hypothetical protein | | | - |
| **SAS030** | HP | N315 | 3.47 | | Hypothetical protein | | | - |
| **SAS031** | HP | N315 | 3.25 | | Hypothetical protein | | | - |
| **SAS034** | HP | N315 | 3.65 | | Hypothetical protein | | | - |
| **SAS036** | HP | N315 | 3.93 | | Hypothetical protein | | | - |
| **SAS040** | HP | N315 | 3.78 | | Hypothetical protein | | | - |
| **SAS048** | HP | N315 | 3.06 | | Hypothetical protein | | | - |
| **SAS060** | HP | N315 | 4.74 | | Hypothetical protein | | | - |
| **SAS073** | HP | N315 | 5.04 | | Hypothetical protein | | | - |
| **SAS090** | HP | N315 | 3.25 | | Hypothetical protein | | | - |
| **SA0019** | HP | N315 | 0.15 | 0.07 | Hypothetical protein | | | - |
| **SA0287** | HP | N315 | 0.27 | 0.27 | Hypothetical protein | | | - |
| **SA0289** | HP | N315 | 0.26 | 0.25 | Hypothetical protein | | | - |
| **SA0290** | HP | N315 | 0.10 | 0.26 | Hypothetical protein | | | - |
| **SA0314** | HP | N315 | 0.13 | 0.12 | Hypothetical protein | | | - |
| **SA0350** | HP | N315 | 0.18 | 0.24 | Hypothetical protein | | | - |
| **SA0407** | HP | N315 | 0.26 | 0.27 | Hypothetical protein | | | - |
| **SA0624** | HP | N315 | 0.16 | 0.16 | Hypothetical protein | | | - |
| **SA0718** | HP | N315 | 0.02 | 0.02 | Hypothetical protein | | | - |
| **SA0721** | HP | N315 | 0.10 | 0.09 | Hypothetical protein | | | - |
| **SA0739** | HP | N315 | 0.32 | 0.27 | Hypothetical protein | | | - |
| **SA0775** | HP | N315 | 0.07 | 0.23 | Hypothetical protein | | | - |
| **SA0778** | HP | N315 | 0.14 | 0.22 | Hypothetical protein | | | - |
| **SA0783** | HP | N315 | 0.21 | 0.13 | Hypothetical protein | | | - |
| **SA0941** | HP | N315 | 0.23 | 0.12 | Hypothetical protein | | | - |
| **SA0954** | HP | N315 | 0.07 | 0.21 | Hypothetical protein | | | - |
| **SA1068** | HP | N315 | 0.15 | 0.16 | Hypothetical protein | | | - |
| **SA1124** | HP | N315 | 0.04 | 0.04 | Hypothetical protein | | | - |
| **SA1135** | HP | N315 | 0.04 | 0.07 | Hypothetical protein | | | - |
| **SA1256** | HP | N315 | 0.18 | 0.17 | Hypothetical protein | | | - |
| **SA1293** | HP | N315 | 0.16 | 0.18 | Hypothetical protein | | | - |
| **SA1295** | HP | N315 | 0.21 | 0.23 | Hypothetical protein | | | - |
| **SA1296** | HP | N315 | 0.14 | 0.10 | Hypothetical protein | | | - |
| **SA1325** | HP | N315 | 0.23 | 0.30 | Hypothetical protein | | | - |
| **SA1356** | HP | N315 | 0.03 | 0.07 | Hypothetical protein | | | - |
| **SA1376** | HP | N315 | 0.20 | 0.19 | Hypothetical protein | | | - |
| **SA1378** | HP | N315 | 0.03 | 0.04 | Hypothetical protein | | | - |
| **SA1392** | HP | N315 | 0.24 | 0.31 | Hypothetical protein | | | - |
| **SA1423** | HP | N315 | 0.01 | 0.03 | Hypothetical protein | | | - |
| **SA1445** | HP | N315 | 0.07 | 0.04 | Hypothetical protein | | | - |
| **SA1509** | HP | N315 | 0.21 | 0.21 | Hypothetical protein | | | - |
| **SA1867** | HP | N315 | 0.16 | 0.18 | Hypothetical protein | | | - |
| **SA1916** | HP | N315 | 0.07 | 0.10 | Hypothetical protein | | | - |
| **SA2130** | HP | N315 | 0.10 | 0.08 | Hypothetical protein | | | - |
| **SA2355** | HP | N315 | 0.17 | 0.15 | Hypothetical protein | | | - |

| | | | | | | | | |
|---|---|---|---|---|---|---|---|---|
| **SA0277** | HP | N315 | 0.01 | | Hypothetical protein | | | - |
| **SA0278** | HP | N315 | 0.06 | | Hypothetical protein | | | - |
| **SA0279** | HP | N315 | 0.15 | | Hypothetical protein | | | - |
| **SA0280** | HP | N315 | 0.19 | | Hypothetical protein | | | - |
| **SA0283** | HP | N315 | 0.09 | | Hypothetical protein | | | - |
| **SA0284** | HP | N315 | 0.16 | | Hypothetical protein | | | - |
| **SA0363** | HP | N315 | 0.29 | | Hypothetical protein | | | - |
| **SA0364** | HP | N315 | 0.28 | | Hypothetical protein | | | - |
| **SA0371** | HP | N315 | 0.32 | | Hypothetical protein | | | - |
| **SA0401** | HP | N315 | 0.11 | | Hypothetical protein | | | - |
| **SA0491** | HP | N315 | 0.30 | | Hypothetical protein | | | - |
| **SA0539** | HP | N315 | 0.20 | | Hypothetical protein | | | - |
| **SA0662** | HP | N315 | 0.10 | | Hypothetical protein | | | - |
| **SA0663** | HP | N315 | 0.19 | | Hypothetical protein | | | - |
| **SA0664** | HP | N315 | 0.27 | | Hypothetical protein | | | - |
| **SA0671** | HP | N315 | 0.13 | | Hypothetical protein | | | - |
| **SA0684** | HP | N315 | 0.26 | | Hypothetical protein | | | - |
| **SA0931** | HP | N315 | 0.29 | | Hypothetical protein | | | - |
| **SA0933** | HP | N315 | 0.33 | | Hypothetical protein | | | - |
| **SA1049** | HP | N315 | 0.19 | | Hypothetical protein | | | - |
| **SA1210** | HP | N315 | 0.25 | | Hypothetical protein | | | - |
| **SA1242** | HP | N315 | 0.31 | | Hypothetical protein | | | - |
| **SA1420** | HP | N315 | 0.29 | | Hypothetical protein | | | - |
| **SA1514** | HP | N315 | 0.10 | | Hypothetical protein | | | - |
| **SA1573** | HP | N315 | 0.14 | | Hypothetical protein | | | - |
| **SA1594** | HP | N315 | 0.32 | | Hypothetical protein | | | - |
| **SA1740** | HP | N315 | 0.14 | | Hypothetical protein | | | - |
| **SA1746** | HP | N315 | 0.10 | | Hypothetical protein | | | - |
| **SA1768** | HP | N315 | 0.20 | | Hypothetical protein | | | - |
| **SA1771** | HP | N315 | 0.20 | | Hypothetical protein | | | - |
| **SA1912** | HP | N315 | 0.02 | | Hypothetical protein | | | - |
| **SA1985** | HP | N315 | 0.05 | | Hypothetical protein | | | - |
| **SA1986** | HP | N315 | 0.09 | | Hypothetical protein | | | - |
| **SA2058** | HP | N315 | 0.07 | | Hypothetical protein | | | - |
| **SA2076** | HP | N315 | 0.12 | | Hypothetical protein | | | - |
| **SA2264** | HP | N315 | 0.06 | | Hypothetical protein | | | - |
| **SA2273** | HP | N315 | 0.18 | | Hypothetical protein | | | - |
| **SA2440** | HP | N315 | 0.11 | | Hypothetical protein | | | - |
| **SA2445** | HP | N315 | 0.30 | | Hypothetical protein | | | - |
| **SAS016** | HP | N315 | 0.05 | | Hypothetical protein | | | - |
| **SAS026** | HP | N315 | 0.23 | | Hypothetical protein | | | - |
| **SA1154** | HP | N315 | 5.39 | 5.85 | Hypothetical protein | **COG2008E** | **E** | Amino acid transport and metabolism |
| **SA0345** | HP | N315 | 3.91 | | Hypothetical protein | **COG0646E,COG0685E** | **E** | Amino acid transport and metabolism |
| **SA2229** | HP | N315 | 3.60 | | Hypothetical protein | **COG0531E** | **E** | Amino acid transport and metabolism |
| **SA0212** | HP | N315 | 4.05 | 3.05 | Hypothetical protein | **COG1082G** | **G** | Carbohydrate transport and metabolism |
| **SA0319** | HP | N315 | 3.72 | | Hypothetical protein | **COG3414G** | **G** | Carbohydrate transport and metabolism |
| **SA1737** | HP | N315 | 3.33 | | Hypothetical protein | **COG2706G** | **G** | Carbohydrate transport and metabolism |
| **SA0865** | HP | N315 | 0.14 | 0.13 | Hypothetical protein | **COG0061G** | **G** | Carbohydrate transport and metabolism |
| **SA1593** | HP | N315 | 0.07 | 0.20 | Hypothetical protein | **COG4193G** | **G** | Carbohydrate transport and metabolism |
| **SA1601** | HP | N315 | 3.95 | 3.82 | Hypothetical protein | **COG0239D** | **D** | Cell cycle control, cell division, chromosome partitioning |
| **SA2196** | HP | N315 | 4.35 | | Hypothetical protein | **COG2161D** | **D** | Cell cycle control, cell division, chromosome partitioning |
| **SA0467** | HP | N315 | 0.14 | 0.23 | Hypothetical protein | **COG0037D** | **D** | Cell cycle control, cell division, chromosome partitioning |
| **SA0191** | HP | N315 | 4.81 | 3.34 | Hypothetical protein | **COG0845M** | **M** | Cell wall/membrane/envelope biogenesis |
| **SA1764** | HP | N315 | 3.71 | | Hypothetical protein | **COG3064M** | **M** | Cell wall/membrane/envelope biogenesis |
| **SA1822** | HP | N315 | 4.57 | | Hypothetical protein | **COG5577M** | **M** | Cell wall/membrane/envelope biogenesis |
| **SA0349** | HP | N315 | 0.29 | 0.22 | Hypothetical protein | **COG0668M** | **M** | Cell wall/membrane/envelope biogenesis |
| **SA0529** | HP | N315 | 0.32 | | Hypothetical protein | **COG0794M** | **M** | Cell wall/membrane/envelope biogenesis |
| **SA1105** | HP | N315 | 0.15 | | Hypothetical protein | **COG0750M** | **M** | Cell wall/membrane/envelope biogenesis |
| **SA2157** | HP | N315 | 0.18 | | Hypothetical protein | **COG1887M** | **M** | Cell wall/membrane/envelope biogenesis |
| **SA2350** | HP | N315 | 0.32 | | Hypothetical protein | **COG0463M** | **M** | Cell wall/membrane/envelope biogenesis |
| **SA1363** | HP | N315 | 3.12 | | Hypothetical protein | **COG0095H** | **H** | Coenzyme transport and metabolism |
| **SA0174** | HP | N315 | 0.04 | 0.08 | Hypothetical protein | **COG2091H** | **H** | Coenzyme transport and metabolism |
| **SA0546** | HP | N315 | 0.22 | 0.20 | Hypothetical protein | **COG0095H** | **H** | Coenzyme transport and metabolism |
| **SA0666** | HP | N315 | 0.06 | 0.09 | Hypothetical protein | **COG0720H** | **H** | Coenzyme transport and metabolism |
| **SA1066** | HP | N315 | 0.29 | 0.23 | Hypothetical protein | **COG1564H** | **H** | Coenzyme transport and metabolism |
| **SA1421** | HP | N315 | 0.14 | 0.20 | Hypothetical protein | **COG1713H** | **H** | Coenzyme transport and metabolism |
| **SA1422** | HP | N315 | 0.04 | 0.06 | Hypothetical protein | **COG1057H** | **H** | Coenzyme transport and metabolism |
| **SA0323** | HP | N315 | 3.40 | 3.22 | Hypothetical protein | **COG0534V** | **V** | Defense mechanisms |
| **SA2150** | HP | N315 | 3.01 | | Hypothetical protein | **COG0577V** | **V** | Defense mechanisms |
| **SA1683** | HP | N315 | 0.27 | 0.29 | Hypothetical protein | **COG1132V** | **V** | Defense mechanisms |
| **SA0581** | HP | N315 | 0.13 | 4.59 | Hypothetical protein | **COG0651CP** | **c** | Energy production and conversion |
| **SA1236** | HP | N315 | 0.29 | 0.22 | Hypothetical protein | **COG1254C** | **C** | Energy production and conversion |
| **SA0578** | HP | N315 | 0.27 | | Hypothetical protein | **COG2111P,COG1009CF** | **c** | Energy production and conversion |
| **SA2156** | HP | N315 | 0.03 | | Hypothetical protein | **COG1620C** | **C** | Energy production and conversion |
| **SA0081** | HP | N315 | 5.28 | 4.67 | Hypothetical protein | **COG1937S** | **S** | Function unknown |
| **SA0170** | HP | N315 | 3.71 | 3.64 | Hypothetical protein | **COG5609S** | **S** | Function unknown |
| **SA0574** | HP | N315 | 3.30 | 3.04 | Hypothetical protein | **COG3238S** | **S** | Function unknown |
| **SA0801** | HP | N315 | 5.92 | 4.57 | Hypothetical protein | **COG0316S** | **S** | Function unknown |
| **SA1130** | HP | N315 | 3.57 | 4.27 | Hypothetical protein | **COG1692S** | **S** | Function unknown |
| **SA1265** | HP | N315 | 4.11 | 3.29 | Hypothetical protein | **COG1738S** | **S** | Function unknown |
| **SA2238** | HP | N315 | 3.36 | 3.18 | Hypothetical protein | **COG4430S** | **S** | Function unknown |
| **SA1344** | HP | N315 | 5.43 | 0.11 | Hypothetical protein | **COG4129S** | **S** | Function unknown |
| **SA0054** | HP | N315 | 3.35 | | Hypothetical protein | **COG4333S** | **S** | Function unknown |
| **SA0102** | HP | N315 | 3.30 | | Hypothetical protein | **COG4716S** | **S** | Function unknown |
| **SA0324** | HP | N315 | 3.84 | | Hypothetical protein | **COG4815S** | **S** | Function unknown |
| **SA0428** | HP | N315 | 3.28 | | Hypothetical protein | **COG5438S** | **S** | Function unknown |
| **SA0892** | HP | N315 | 3.85 | | Hypothetical protein | **COG2314S** | **S** | Function unknown |
| **SA1014** | HP | N315 | 3.30 | | Hypothetical protein | **COG1288S** | **S** | Function unknown |
| **SA1020** | HP | N315 | 3.17 | | Hypothetical protein | **COG4365S** | **S** | Function unknown |
| **SA1050** | HP | N315 | 4.01 | | Hypothetical protein | **COG3865S** | **S** | Function unknown |
| **SA1057** | HP | N315 | 4.48 | | Hypothetical protein | **COG2314S** | **S** | Function unknown |
| **SA1937** | HP | N315 | 3.86 | | Hypothetical protein | **COG4270S** | **S** | Function unknown |
| **SA2133** | HP | N315 | 3.66 | | Hypothetical protein | **COG3152S** | **S** | Function unknown |
| **SA2219** | HP | N315 | 3.39 | | Hypothetical protein | **COG2246S** | **S** | Function unknown |
| **SA2299** | HP | N315 | 3.17 | | Hypothetical protein | **COG1289S** | **S** | Function unknown |
| **SA2378** | HP | N315 | 3.17 | | Hypothetical protein | **COG2764S** | **S** | Function unknown |
| **SA0012** | HP | N315 | 0.28 | 0.33 | Hypothetical protein | **COG4241S** | **S** | Function unknown |
| **SA0020** | HP | N315 | 0.13 | 0.11 | Hypothetical protein | **COG4853S** | **S** | Function unknown |
| **SA0489** | HP | N315 | 0.10 | 0.17 | Hypothetical protein | **COG1939S** | **S** | Function unknown |
| **SA0636** | HP | N315 | 0.21 | 0.14 | Hypothetical protein | **COG1671S** | **S** | Function unknown |
| **SA0722** | HP | N315 | 0.27 | 0.30 | Hypothetical protein | **COG1481S** | **S** | Function unknown |
| **SA0789** | HP | N315 | 0.25 | 0.18 | Hypothetical protein | **COG2445S** | **S** | Function unknown |
| **SA1021** | HP | N315 | 0.28 | 0.30 | Hypothetical protein | **COG2001S** | **S** | Function unknown |
| **SA1079** | HP | N315 | 0.08 | 0.08 | Hypothetical protein | **COG2739S** | **S** | Function unknown |
| **SA1125** | HP | N315 | 0.04 | 0.05 | Hypothetical protein | **COG1426S** | **S** | Function unknown |
| **SA1173** | HP | N315 | 0.09 | 0.04 | Hypothetical protein | **COG2340S** | **S** | Function unknown |
| **SA1258** | HP | N315 | 0.21 | 0.27 | Hypothetical protein | **COG1307S** | **S** | Function unknown |
| **SA1314** | HP | N315 | 0.20 | 0.22 | Hypothetical protein | **COG4955S** | **S** | Function unknown |
| **SA1326** | HP | N315 | 0.13 | 0.20 | Hypothetical protein | **COG1354S** | **S** | Function unknown |
| **SA1402** | HP | N315 | 0.02 | 0.03 | Hypothetical protein | **COG4864S** | **S** | Function unknown |
| **SA1661** | HP | N315 | 0.06 | 0.05 | Hypothetical protein | **COG4717S** | **S** | Function unknown |
| **SA1702** | HP | N315 | 0.25 | 0.20 | Hypothetical protein | **COG4758S** | **S** | Function unknown |
| **SA1723** | HP | N315 | 0.27 | 0.29 | Hypothetical protein | **COG4496S** | **S** | Function unknown |
| **SA1727** | HP | N315 | 0.11 | 0.15 | Hypothetical protein | **COG4843S** | **S** | Function unknown |
| **SA1741** | HP | N315 | 0.07 | 0.15 | Hypothetical protein | **COG4709S** | **S** | Function unknown |
| **SA1876** | HP | N315 | 0.17 | 0.19 | Hypothetical protein | **COG3402S** | **S** | Function unknown |
| **SA1877** | HP | N315 | 0.21 | 0.29 | Hypothetical protein | **COG3428S** | **S** | Function unknown |
| **SAS001** | HP | N315 | 0.22 | 0.19 | Hypothetical protein | **COG4392S** | **S** | Function unknown |
| **SA1388** | HP | N315 | 0.26 | | Hypothetical protein | **COG0327S,COG3323S** | **S** | Function unknown |
| **SA0275** | HP | N315 | 0.21 | | Hypothetical protein | **COG4499S** | **S** | Function unknown |
| **SA0481** | HP | N315 | 0.18 | | Hypothetical protein | **COG3880S** | **S** | Function unknown |
| **SA0680** | HP | N315 | 0.28 | | Hypothetical protein | **COG4502S** | **S** | Function unknown |
| **SA0966** | HP | N315 | 0.32 | | Hypothetical protein | **COG2322S** | **S** | Function unknown |
| **SA1030** | HP | N315 | 0.23 | | Hypothetical protein | **COG1496S** | **S** | Function unknown |
| **SA1406** | HP | N315 | 0.30 | | Hypothetical protein | **COG1385S** | **S** | Function unknown |
| **SA1966** | HP | N315 | 0.25 | | Hypothetical protein | **COG4856S** | **S** | Function unknown |
| **SA1209** | HP | N315 | 4.55 | 3.37 | Hypothetical protein | **COG0561R** | **R** | General function prediction only |
| **SA1840** | HP | N315 | 4.47 | 3.52 | Hypothetical protein | **COG3560R** | **R** | General function prediction only |
| **SA0425** | HP | N315 | 3.03 | | Hypothetical protein | **COG0494LR** | **r** | General function prediction only |
| **SA2383** | HP | N315 | 5.07 | | Hypothetical protein | **COG0500QR** | **r** | General function prediction only |
| **SA0840** | HP | N315 | 4.17 | | Hypothetical protein | **COG1881R** | **R** | General function prediction only |
| **SA1335** | HP | N315 | 3.13 | | Hypothetical protein | **COG1234R** | **R** | General function prediction only |
| **SA1649** | HP | N315 | 3.53 | | Hypothetical protein | **COG3545R** | **R** | General function prediction only |
| **SA1692** | HP | N315 | 3.78 | | Hypothetical protein | **COG0693R** | **R** | General function prediction only |
| **SA1739** | HP | N315 | 3.10 | | Hypothetical protein | **COG4927R** | **R** | General function prediction only |
| **SA2225** | HP | N315 | 3.31 | | Hypothetical protein | **COG1409R** | **R** | General function prediction only |
| **SA2313** | HP | N315 | 3.93 | | Hypothetical protein | **COG0561R** | **R** | General function prediction only |
| **SA0485** | HP | N315 | 0.22 | 0.26 | Hypothetical protein | **COG4956R** | **R** | General function prediction only |
| **SA0560** | HP | N315 | 0.15 | 0.14 | Hypothetical protein | **COG1078R** | **R** | General function prediction only |
| **SA0667** | HP | N315 | 0.09 | 0.08 | Hypothetical protein | **COG0603R** | **R** | General function prediction only |
| **SA0720** | HP | N315 | 0.06 | 0.08 | Hypothetical protein | **COG1660R** | **R** | General function prediction only |
| **SA0932** | HP | N315 | 0.28 | 0.24 | Hypothetical protein | **COG1092R** | **R** | General function prediction only |
| **SA0940** | HP | N315 | 0.11 | 0.03 | Hypothetical protein | **COG0595R** | **R** | General function prediction only |
| **SA0975** | HP | N315 | 0.17 | 0.14 | Hypothetical protein | **COG1399R** | **R** | General function prediction only |
| **SA0999** | HP | N315 | 0.15 | 0.16 | Hypothetical protein | **COG0622R** | **R** | General function prediction only |
| **SA1031** | HP | N315 | 0.23 | 0.26 | Hypothetical protein | **COG0325R** | **R** | General function prediction only |
| **SA1061** | HP | N315 | 0.12 | 0.16 | Hypothetical protein | **COG0820R** | **R** | General function prediction only |
| **SA1064** | HP | N315 | 0.21 | 0.20 | Hypothetical protein | **COG1162R** | **R** | General function prediction only |
| **SA1224** | HP | N315 | 0.11 | 0.11 | Hypothetical protein | **COG0488R** | **R** | General function prediction only |

| | | | | | | | | |
|---|---|---|---|---|---|---|---|---|
| **SA1379** | HP | N315 | 0.11 | 0.13 | Hypothetical protein | **COG0705R** | **R** | General function prediction only |
| **SA1393** | HP | N315 | 0.24 | 0.32 | Hypothetical protein | **COG0517R** | **R** | General function prediction only |
| **SA1399** | HP | N315 | 0.08 | 0.13 | Hypothetical protein | **COG0319R** | **R** | General function prediction only |
| **SA1425** | HP | N315 | 0.02 | 0.05 | Hypothetical protein | **COG1161R** | **R** | General function prediction only |
| **SA1426** | HP | N315 | 0.10 | 0.21 | Hypothetical protein | **COG2179R** | **R** | General function prediction only |
| **SA1526** | HP | N315 | 0.14 | 0.11 | Hypothetical protein | **COG0618R** | **R** | General function prediction only |
| **SA1536** | HP | N315 | 0.08 | 0.08 | Hypothetical protein | **COG0730R** | **R** | General function prediction only |
| **SA1576** | HP | N315 | 0.22 | 0.24 | Hypothetical protein | **COG2081R** | **R** | General function prediction only |
| **SA1892** | HP | N315 | 0.20 | 0.20 | Hypothetical protein | **COG1418R** | **R** | General function prediction only |
| **SA2050** | HP | N315 | 0.32 | 0.27 | Hypothetical protein | **COG2252R** | **R** | General function prediction only |
| **SA0621** | HP | N315 | 0.21 | | Hypothetical protein | **COG0670R** | **R** | General function prediction only |
| **SA1185** | HP | N315 | 0.32 | | Hypothetical protein | **COG0824R** | **R** | General function prediction only |
| **SA1274** | HP | N315 | 0.24 | | Hypothetical protein | **COG0699R** | **R** | General function prediction only |
| **SA1389** | HP | N315 | 0.25 | | Hypothetical protein | **COG2384R** | **R** | General function prediction only |
| **SA0581** | HP | N315 | 0.13 | 4.59 | Hypothetical protein | **COG0651CP** | **p** | Inorganic ion transport and metabolism |
| **SA0806** | HP | N315 | 3.69 | | Hypothetical protein | **COG2072P** | **P** | Inorganic ion transport and metabolism |
| **SA0868** | HP | N315 | 0.06 | 0.13 | Hypothetical protein | **COG0569P,COG0475F** | **P** | Inorganic ion transport and metabolism |
| **SA0585** | HP | N315 | 0.14 | 0.11 | Hypothetical protein | **COG0025P** | **P** | Inorganic ion transport and metabolism |
| **SA0588** | HP | N315 | 0.11 | 0.06 | Hypothetical protein | **COG1108P** | **P** | Inorganic ion transport and metabolism |
| **SA0872** | HP | N315 | 0.30 | 0.23 | Hypothetical protein | **COG2382P** | **P** | Inorganic ion transport and metabolism |
| **SA1240** | HP | N315 | 0.33 | 0.28 | Hypothetical protein | **COG4548P** | **P** | Inorganic ion transport and metabolism |
| **SA1578** | HP | N315 | 0.21 | 0.13 | Hypothetical protein | **COG0607P** | **P** | Inorganic ion transport and metabolism |
| **SA2019** | HP | N315 | 0.11 | 0.18 | Hypothetical protein | **COG0619P** | **P** | Inorganic ion transport and metabolism |
| **SA0578** | HP | N315 | 0.27 | | Hypothetical protein | **COG2111P,COG1009CF** | **P** | Inorganic ion transport and metabolism |
| **SA0939** | HP | N315 | 0.05 | | Hypothetical protein | **COG0569P** | **P** | Inorganic ion transport and metabolism |
| **SA0335** | HP | N315 | 3.89 | | Hypothetical protein | **COG1826U** | **U** | Intracellular trafficking, secretion, and vesicular transport |
| **SA0406** | HP | N315 | 0.24 | | Hypothetical protein | **COG5153UI** | **u** | Intracellular trafficking, secretion, and vesicular transport |
| **SA0227** | HP | N315 | 6.56 | 4.24 | Hypothetical protein | **COG4670I** | **I** | Lipid transport and metabolism |
| **SA1694** | HP | N315 | 0.17 | 0.18 | Hypothetical protein | **COG1607I** | **I** | Lipid transport and metabolism |
| **SA0406** | HP | N315 | 0.24 | | Hypothetical protein | **COG5153UI** | **i** | Lipid transport and metabolism |
| **SA0784** | HP | N315 | 0.11 | 0.09 | Hypothetical protein | **COG0737F** | **F** | Nucleotide transport and metabolism |
| **SA0919** | HP | N315 | 0.03 | 0.02 | Hypothetical protein | **COG1828F** | **F** | Nucleotide transport and metabolism |
| **SA1849** | HP | N315 | 3.33 | | Hypothetical protein | **COG0425O** | **O** | Posttranslational modification, protein turnover, chaperones |
| **SA2370** | HP | N315 | 11.71 | | Hypothetical protein | **COG0492O** | **O** | Posttranslational modification, protein turnover, chaperones |
| **SA1403** | HP | N315 | 0.02 | 0.02 | Hypothetical protein | **COG1030O** | **O** | Posttranslational modification, protein turnover, chaperones |
| **SA0425** | HP | N315 | 3.03 | | Hypothetical protein | **COG0494LR** | **l** | Replication, recombination and repair |
| **SA1415** | HP | N315 | 3.01 | | Hypothetical protein | **COG1466L** | **L** | Replication, recombination and repair |
| **SA1093** | HP | N315 | 0.09 | 0.18 | Hypothetical protein | **COG0550L,COG0551L** | **L** | Replication, recombination and repair |
| **SA0449** | HP | N315 | 0.18 | 0.11 | Hypothetical protein | **COG0084L** | **L** | Replication, recombination and repair |
| **SA0450** | HP | N315 | 0.09 | 0.09 | Hypothetical protein | **COG1658L** | **L** | Replication, recombination and repair |
| **SA1277** | HP | N315 | 0.22 | 0.13 | Hypothetical protein | **COG0116L** | **L** | Replication, recombination and repair |
| **SA1454** | HP | N315 | 0.14 | 0.14 | Hypothetical protein | **COG2256L** | **L** | Replication, recombination and repair |
| **SA1534** | HP | N315 | 0.16 | 0.11 | Hypothetical protein | **COG0827L** | **L** | Replication, recombination and repair |
| **SA1662** | HP | N315 | 0.21 | 0.25 | Hypothetical protein | **COG0420L** | **L** | Replication, recombination and repair |
| **SA0972** | HP | N315 | 0.31 | | Hypothetical protein | **COG0742L** | **L** | Replication, recombination and repair |
| **SA2383** | HP | N315 | 5.07 | | Hypothetical protein | **COG0500QR** | **q** | Secondary metabolites biosynthesis, transport and catabolism |
| **SA1071** | HP | N315 | 0.20 | 0.16 | Hypothetical protein | **COG2050Q** | **Q** | Secondary metabolites biosynthesis, transport and catabolism |
| **SA0959** | HP | N315 | 0.13 | 0.26 | Hypothetical protein | **COG1217T** | **T** | Signal transduction mechanisms |
| **SA1062** | HP | N315 | 0.20 | 0.19 | Hypothetical protein | **COG0631T** | **T** | Signal transduction mechanisms |
| **SA1873** | HP | N315 | 0.21 | | Hypothetical protein | **COG2337T** | **T** | Signal transduction mechanisms |
| **SA2181** | HP | N315 | 0.24 | | Hypothetical protein | **COG2203T** | **T** | Signal transduction mechanisms |
| **SA2105** | HP | N315 | 3.44 | 3.68 | Hypothetical protein | **COG2378K** | **K** | Transcription |
| **SA0908** | HP | N315 | 3.24 | 0.27 | Hypothetical protein | **COG1316K** | **K** | Transcription |
| **SA0120** | HP | N315 | 3.07 | | Hypothetical protein | **COG1475K** | **K** | Transcription |
| **SA0492** | HP | N315 | 4.60 | | Hypothetical protein | **COG1595K** | **K** | Transcription |
| **SA1110** | HP | N315 | 0.01 | 0.01 | Hypothetical protein | **COG2740K** | **K** | Transcription |
| **SA1868** | HP | N315 | 0.28 | 0.21 | Hypothetical protein | **COG2183K** | **K** | Transcription |
| **SA1925** | HP | N315 | 0.23 | 0.22 | Hypothetical protein | **COG1733K** | **K** | Transcription |
| **SA0873** | HP | N315 | 3.58 | 3.95 | Hypothetical protein | **COG1514J** | **J** | Translation, ribosomal structure and biogenesis |
| **SA1684** | HP | N315 | 3.15 | 3.52 | Hypothetical protein | **COG3557J** | **J** | Translation, ribosomal structure and biogenesis |
| **SA0838** | HP | N315 | 0.08 | 6.58 | Hypothetical protein | **COG0009J** | **J** | Translation, ribosomal structure and biogenesis |
| **SA0707** | HP | N315 | 3.23 | | Hypothetical protein | **COG1544J** | **J** | Translation, ribosomal structure and biogenesis |
| **SA0464** | HP | N315 | 0.18 | 0.06 | Hypothetical protein | **COG1188J** | **J** | Translation, ribosomal structure and biogenesis |
| **SA1040** | HP | N315 | 0.04 | 0.04 | Hypothetical protein | **COG0564J** | **J** | Translation, ribosomal structure and biogenesis |
| **SA1134** | HP | N315 | 0.15 | 0.05 | Hypothetical protein | **COG0621J** | **J** | Translation, ribosomal structure and biogenesis |
| **SA1668** | HP | N315 | 0.09 | 0.21 | Hypothetical protein | **COG0564J** | **J** | Translation, ribosomal structure and biogenesis |
| **SA1405** | HP | N315 | 0.18 | | Hypothetical protein | **COG0621J** | **J** | Translation, ribosomal structure and biogenesis |
| **SA0715** | *hprK* | N315 | 0.16 | 0.16 | HPr kinase/phosphatase | **COG1493T** | **T** | Signal transduction mechanisms |
| **SA0391** | *hsdM* | N315 | 0.20 | 0.30 | probable type I sitespecific deoxyribonuclease LldI chain hsdM | **COG0286V** | **V** | Defense mechanisms |
| **SA0879** | *htrA* | N315 | 0.24 | 0.24 | serine protease HtrA | **COG0265O** | **O** | Posttranslational modification, protein turnover, chaperones |
| **SA2459** | *icaA* | N315 | 3.92 | 3.64 | intercellular adhesion protein A | **COG1215M** | **M** | Cell wall/membrane/envelope biogenesis |
| **SA2461** | *icaB* | N315 | 3.75 | | intercellular adhesion protein B | | | - |
| **SA2462** | *icaC* | N315 | 3.91 | 5.18 | intercellular adhesion protein C | **COG3936G** | **G** | Carbohydrate transport and metabolism |
| **MW1076** | *ileS* | MW2 | 0.26 | 0.18 | IletRNA synthetase | **COG0060J** | **J** | Translation, ribosomal structure and biogenesis |
| **SA1859** | *ilvB* | N315 | 0.08 | 0.08 | acetolactate synthase large subunit | **COG0028EH** | **e** | Amino acid transport and metabolism |
| **SA1858** | *ilvD* | N315 | 4.39 | | dihydroxyacid dehydratase | | | - |
| **SA2026** | *infA* | N315 | 0.01 | 0.16 | translation initiation factor IF1 | **COG0361J** | **J** | Translation, ribosomal structure and biogenesis |
| **SA1112** | *infB* | N315 | 0.02 | 0.14 | translation initiation factor IF2 | **COG0532J** | **J** | Translation, ribosomal structure and biogenesis |
| **SA1504** | *infC* | N315 | 0.33 | | translation initiation factor IF3 | **COG0290J** | **J** | Translation, ribosomal structure and biogenesis |
| **SAV0847** | *int* | Mu50 | | 3.48 | integrase | | | - |
| **SA2431** | *isaB* | N315 | 5.05 | 3.52 | immunodominant antigen B | | | - |
| **SA0981** | *isdF* | N315 | 3.34 | | ferrichrome ABC transporter | **COG0609P** | **P** | Inorganic ion transport and metabolism |
| **SA1170** | *katA* | N315 | 0.25 | 0.32 | Catalase | **COG0753P** | **P** | Inorganic ion transport and metabolism |
| **SA0067** | *kdpD* | N315 | 5.81 | 3.91 | kdp operon sensor protein | **COG2205T** | **T** | Signal transduction mechanisms |
| **SA1882** | *kdpD* | N315 | 0.05 | 0.21 | sensor protein KdpD | **COG2205T** | **T** | Signal transduction mechanisms |
| **SA1997** | *lacA* | N315 | 3.37 | | galactose6phosphate isomerase LacA subunit | **COG0698G** | **G** | Carbohydrate transport and metabolism |
| **SA1996** | *lacB* | N315 | 62.50 | 65.36 | galactose6phosphate isomerase LacB subunit | **COG0698G** | **G** | Carbohydrate transport and metabolism |
| **SA1995** | *lacC* | N315 | 3.22 | 3.14 | tagatose6phosphate kinase | **COG1105G** | **G** | Carbohydrate transport and metabolism |
| **SA1994** | *lacD* | N315 | 3.30 | 3.82 | tagatose 1,6diphosphate aldolase | **COG3684G** | **G** | Carbohydrate transport and metabolism |
| **SA0232** | *lctE* | N315 | 0.29 | 0.20 | Llactate dehydrogenase | **COG0039C** | **C** | Energy production and conversion |
| **SA1413** | *lepA* | N315 | 0.26 | 0.27 | GTPbinding protein | **COG0481M** | **M** | Cell wall/membrane/envelope biogenesis |
| **SA1579** | *leuS* | N315 | 0.05 | 0.15 | leucyltRNA synthetase | **COG0495J** | **J** | Translation, ribosomal structure and biogenesis |
| **SA0716** | *lgt* | N315 | 0.29 | 0.24 | prolipoprotein diacylglyceryl transferase | | | - |
| **SA1720** | *lig* | N315 | 0.17 | 0.31 | DNA ligase | **COG0272L** | **L** | Replication, recombination and repair |
| **SA1638** | *lukE* | N315 | 3.06 | | leukotoxin LukE | | | - |
| **SA1232** | *lysA* | N315 | 0.07 | 0.12 | diaminopimelate decarboxylase | **COG0019E** | **E** | Amino acid transport and metabolism |
| **SA1225** | *lysC* | N315 | 0.09 | 0.11 | aspartokinase II | **COG0527E** | **E** | Amino acid transport and metabolism |
| **MW1625** | *lysP* | MW2 | 0.32 | | lysinespecific permease | **COG0833E** | **E** | Amino acid transport and metabolism |
| **SA0475** | *lysS* | N315 | 0.05 | 0.13 | lysyltRNA synthetase | **COG1190J** | **J** | Translation, ribosomal structure and biogenesis |
| **SA1757** | *lytA* | N315 | 3.69 | 3.24 | truncated amidase | | | - |
| **SA0265** | *lytM* | N315 | 14.88 | | peptidoglycan hydrolase | **COG0739M** | **M** | Cell wall/membrane/envelope biogenesis |
| **SA1338** | *malA* | N315 | 3.21 | | alphaD1,4glucosidase | | | - |
| **SA1704** | *map* | N315 | 3.98 | 3.72 | methionyl aminopeptidase map | **COG0024J** | **J** | Translation, ribosomal structure and biogenesis |
| **SA1751** | *mapW* | N315 | 0.23 | | truncated mapw protein | | | - |
| **SA0038** | *mecA* | N315 | 3.32 | 3.50 | penicillin binding protein 2' | **COG0768M** | **M** | Cell wall/membrane/envelope biogenesis |
| **SA1614** | *menC* | N315 | | 0.23 | osuccinylbenzoic acid synthetase | **COG4948MR** | **m** | Cell wall/membrane/envelope biogenesis |
| **SA0896** | *menD* | N315 | 0.23 | 0.26 | menaquinone biosynthesis protein | **COG1165H** | **H** | Coenzyme transport and metabolism |
| **SA0461** | *mfd* | N315 | 0.29 | | transcriptionrepair coupling factor | **COG1197LK** | **l** | Replication, recombination and repair |
| **SA1913** | *mnaA* | N315 | 0.01 | 0.07 | UDPGlcNAc 2epimerase | **COG0381M** | **M** | Cell wall/membrane/envelope biogenesis |
| **SA0813** | *mnhA* | N315 | 0.32 | | Na+/H+ antiporter subunit | **COG2111P,COG1009CF** | **c** | Energy production and conversion |
| **SA0810** | *mnhD* | N315 | 0.05 | 0.07 | Na+/H+antiporter subunit | **COG0651CP** | **c** | Energy production and conversion |
| **SA0808** | *mnhF* | N315 | | 0.21 | Na+/H+ antiporter subunit | **COG2212P** | **P** | Inorganic ion transport and metabolism |
| **SA2063** | *moaA* | N315 | 0.32 | | molybdenum cofactor biosynthesis protein A | **COG2896H** | **H** | Coenzyme transport and metabolism |
| **SA2070** | *moaB* | N315 | 0.22 | 0.19 | molybdopterin precursor biosynthesis moaB | | | - |
| **MW2192** | *moaC* | MW2 | 0.21 | | molybdenum cofactor biosynthesis protein C | | | - |
| **SA2065** | *moaD* | N315 | 0.31 | 0.28 | probable molybdopterin synthase small subunit | **COG1977H** | **H** | Coenzyme transport and metabolism |
| **SA2066** | *moaE* | N315 | 0.09 | 0.11 | molybdopterin converting factor moa | | | - |
| **SA2064** | *mobA* | N315 | 0.15 | 0.24 | molybdopteringuanine dinucleotide biosynthesis mobA | **COG0746H** | **H** | Coenzyme transport and metabolism |
| **SA2067** | *mobB* | N315 | 0.06 | 0.14 | probable molybdopteringuanine dinucleotide biosynthesis mob | **COG1763H** | **H** | Coenzyme transport and metabolism |
| **SA2073** | *modB* | N315 | 0.31 | | probable molybdenum transport permease | **COG4149P** | **P** | Inorganic ion transport and metabolism |
| **SA2072** | *modC* | N315 | 0.20 | | molybdenum transport ATPbinding protein ModC | **COG4148P** | **P** | Inorganic ion transport and metabolism |
| **SA2068** | *moeA* | N315 | 0.16 | 0.28 | molybdopterin biosynthesis protein moeA | **COG0303H** | **H** | Coenzyme transport and metabolism |
| **SA2071** | *moeB* | N315 | 0.16 | 0.24 | molybdopterin biosynthesis protein moeB | **COG0476H** | **H** | Coenzyme transport and metabolism |
| **SA0583** | *mprF* | N315 | | 0.22 | Na+/H+ antiporter | **COG2212P** | **P** | Inorganic ion transport and metabolism |
| **SA2400** | *mqo2* | N315 | 0.11 | 0.06 | malate:quinone oxidoreductase | **COG0579R** | **R** | General function prediction only |
| **SA1025** | *mraY* | N315 | 0.22 | 0.26 | phosphoNmuramic acidpentapeptide translocase | **COG0472M** | **M** | Cell wall/membrane/envelope biogenesis |
| **SA1902** | *murA* | N315 | 0.04 | 0.23 | UDPNacetylglucosamine 1carboxyvinyl transferase 1 | **COG0766M** | **M** | Cell wall/membrane/envelope biogenesis |
| **SA1561** | *murC* | N315 | 0.10 | 0.19 | UDPNAacerylmuramatealanine ligase | **COG0773M** | **M** | Cell wall/membrane/envelope biogenesis |
| **SA1026** | *murD* | N315 | 0.04 | 0.08 | UDPNacetylmuramoylalanineDglutamate ligase | **COG0771M** | **M** | Cell wall/membrane/envelope biogenesis |
| **SA1886** | *murF* | N315 | 0.07 | 0.08 | UDPNacetylmuramoylalanylDglutamyl2, 6diaminopimelateDala | **COG0770M** | **M** | Cell wall/membrane/envelope biogenesis |
| **SA1251** | *murG* | N315 | 0.28 | 0.31 | undecaprenylPPMurNAcpentapeptideUDPGlcNAc GlcNAc tran | **COG0707M** | **M** | Cell wall/membrane/envelope biogenesis |
| **SA0997** | *murI* | N315 | 0.09 | 0.11 | glutamate racemase | **COG0796M** | **M** | Cell wall/membrane/envelope biogenesis |
| **SA1926** | *murZ* | N315 | 0.14 | 0.17 | UDPNacetylglucosamine 1carboxylvinyl transferase 2 | **COG0766M** | **M** | Cell wall/membrane/envelope biogenesis |
| **SA1137** | *mutS* | N315 | 0.04 | 0.17 | DNA mismatch repair protein | **COG0249L** | **L** | Replication, recombination and repair |
| **SA0991** | *mutS2* | N315 | 0.29 | | MutSlike protein | **COG1193L** | **L** | Replication, recombination and repair |
| **SA0549** | *mvaK2* | N315 | 0.22 | | phosphomevalonate kinase | **COG1577I** | **I** | Lipid transport and metabolism |
| **MW0020** | MW0020 | MW2 | | 0.12 | hypothetical protein | | | - |
| **MW0115** | MW0115 | MW2 | | 0.28 | phosphonates transport permease | **COG3639P** | **P** | Inorganic ion transport and metabolism |
| **MW0200** | MW0200 | MW2 | 45.05 | 25.06 | periplasmicironbinding protein BitC | **COG1840P** | **P** | Inorganic ion transport and metabolism |
| **MW0263** | MW0263 | MW2 | | 0.26 | conserved diarrheal toxin | **COG1674D** | **D** | Cell cycle control, cell division, chromosome partitioning |
| **MW0318** | MW0318 | MW2 | | 3.30 | ribosomalproteinserine Nacetyltransferase | **COG1670J** | **J** | Translation, ribosomal structure and biogenesis |
| **MW0327** | MW0327 | MW2 | | 0.32 | ABC transporter ATPbinding protein | **COG1131V** | **V** | Defense mechanisms |
| **MW0379** | MW0379 | MW2 | | 3.98 | hypothetical protein | **COG1432S** | **S** | Function unknown |
| **MW0381** | MW0381 | MW2 | | 4.63 | hypothetical protein | **COG0702MG** | **g** | Carbohydrate transport and metabolism |
| **MW0446** | MW0446 | MW2 | | 0.22 | hypothetical protein | **COG0084L** | **L** | Replication, recombination and repair |
| **MW0539** | MW0539 | MW2 | 0.23 | 0.26 | cationic amino acid transporter | **COG0531E** | **E** | Amino acid transport and metabolism |
| **MW0751** | MW0751 | MW2 | 8.93 | 10.65 | putative primase | **COG3378R,COG4983S** | **s** | Function unknown |
| **MW0761** | MW0761 | MW2 | | 0.24 | hypothetical protein | | | - |
| **MW0778** | MW0778 | MW2 | | 3.75 | phosphoglycerate mutase | **COG0406G** | **G** | Carbohydrate transport and metabolism |

| | | | | | | | | |
|---|---|---|---|---|---|---|---|---|
| **MW0812** | MW0812 | MW2 | | 0.26 | glycerate dehydrogenase | **COG1052CHR** | **h** | Coenzyme transport and metabolism |
| **MW0820** | MW0820 | MW2 | 0.20 | | NADH dehydrogenase | **COG1252C** | **C** | Energy production and conversion |
| **MW0967** | MW0967 | MW2 | | 3.04 | hypothetical protein | **COG0695O** | **O** | Posttranslational modification, protein turnover, chaperones |
| **MW1034** | MW1034 | MW2 | | 0.13 | hypothetical protein | **COG0127F** | **F** | Nucleotide transport and metabolism |
| **MW1071** | MW1071 | MW2 | | 0.20 | hypothetical protein | **COG0325R** | **R** | General function prediction only |
| **MW1173** | MW1173 | MW2 | | 0.25 | 2oxoacid ferredoxin oxidoreductase, beta subunit | **COG1013C** | **C** | Energy production and conversion |
| **MW1225** | MW1225 | MW2 | | 0.11 | hypothetical protein | **COG2340S** | **S** | Function unknown |
| **MW1351** | MW1351 | MW2 | | 0.12 | hypothetical protein | | | - |
| **MW1399** | MW1399 | MW2 | | 5.41 | protease | **COG0740OU** | **u** | Intracellular trafficking, secretion, and vesicular transport |
| **MW1518** | MW1518 | MW2 | 0.13 | 0.23 | DNA repair protein RecO | **COG1381L** | **L** | Replication, recombination and repair |
| **MW1657** | MW1657 | MW2 | | 0.10 | hypothetical protein | **COG0730R** | **R** | General function prediction only |
| **MW1680** | MW1680 | MW2 | 3.37 | 4.67 | chorismate mutase homolog | **COG2876E,COG1605E** | **E** | Amino acid transport and metabolism |
| **MW1696** | MW1696 | MW2 | | 0.11 | 16S pseudouridylate synthase | **COG1187J** | **J** | Translation, ribosomal structure and biogenesis |
| **MW1714** | MW1714 | MW2 | 0.30 | | arsenical pump membrane protein homolog | **COG1055P** | **P** | Inorganic ion transport and metabolism |
| **MW1818** | MW1818 | MW2 | | 0.31 | hypothetical protein | **COG1607I** | **I** | Lipid transport and metabolism |
| **MW1932** | MW1932 | MW2 | 3.05 | | phage anti repressor | **COG3561K,COG3645S** | **s** | Function unknown |
| **MW1975** | MW1975 | MW2 | 0.19 | 0.27 | glycoprotein endopeptidase | **COG1214O** | **O** | Posttranslational modification, protein turnover, chaperones |
| **MW2102** | MW2102 | MW2 | | 0.09 | ferrichrome ABC transporter (permease) | **COG0609P** | **P** | Inorganic ion transport and metabolism |
| **MW2217** | MW2217 | MW2 | | 0.16 | secretory antigen precursor SsaA homolog | **COG3942R** | **R** | General function prediction only |
| **MW2317** | MW2317 | MW2 | 0.10 | 0.09 | nitrate reductase delta chain | **COG2180C** | **C** | Energy production and conversion |
| **SA1728** | *nadE* | N315 | 0.08 | 0.08 | NAD synthetase, prefers NH3 over glutamine | **COG0171H** | **H** | Coenzyme transport and metabolism |
| **MW1853** | *nadE* | MW2 | 0.12 | 0.08 | NAD synthetase | **COG0171H** | **H** | Coenzyme transport and metabolism |
| **SA0304** | *nanA* | N315 | 3.30 | | Nacetylneuraminate lyase subunit | **COG0329EM** | **e** | Amino acid transport and metabolism |
| **SA2182** | *narI* | N315 | 0.33 | 0.33 | nitrate reductase gamma chain | **COG2181C** | **C** | Energy production and conversion |
| **SA2188** | *nasD* | N315 | | 0.33 | nitrite reductase | **COG1251C** | **C** | Energy production and conversion |
| **SA0686** | *nrdE* | N315 | 0.28 | 0.29 | ribonucleoside diphosphate reductase major subunit | **COG0209F** | **F** | Nucleotide transport and metabolism |
| **SA1285** | *nth* | N315 | 3.44 | | endonucleaselike protein | **COG0177L** | **L** | Replication, recombination and repair |
| **SA1109** | *nusA* | N315 | 0.05 | 0.20 | transcription terminationantitermination factor | **COG0195K** | **K** | Transcription |
| **SA0494** | *nusG* | N315 | 0.23 | 0.26 | transcription antitermination protein | **COG0250K** | **K** | Transcription |
| **SA0717** | *OatA* | N315 | | 0.23 | Oacetyltransferase | **COG0110R** | **R** | General function prediction only |
| **SA1470** | *obg* | N315 | 0.29 | | Spo0Bassociated GTPbinding protein | **COG0536R** | **R** | General function prediction only |
| **SA1245** | *odhA* | N315 | 0.02 | 0.31 | 2oxoglutarate dehydrogenase E1 | **COG0567C** | **C** | Energy production and conversion |
| **MW1303** | *odhA* | MW2 | 0.30 | | oxoglutarate dehydrogenase | **COG0567C** | **C** | Energy production and conversion |
| **SA1244** | *odhB* | N315 | 0.05 | 0.09 | dihydrolipoamide succinyltransferase | **COG0508C** | **C** | Energy production and conversion |
| **MW1267** | *opp2F* | MW2 | 0.06 | 0.09 | oligopeptide transporter putative ATPase domain | **COG4608E** | **E** | Amino acid transport and metabolism |
| **SA0845** | *oppB* | N315 | 0.16 | 0.14 | oligopeptide transport system permease protein | **COG0601EP** | **e** | Amino acid transport and metabolism |
| **SA0847** | *oppD* | N315 | 0.05 | 0.05 | oligopeptide transport system ATPbinding protein OppD homol | **COG0444EP** | **e** | Amino acid transport and metabolism |
| **SA0848** | *oppF* | N315 | 0.01 | 0.02 | oligopeptide transport system ATPbinding protein OppF homol | **COG4608E** | **E** | Amino acid transport and metabolism |
| **SA2234** | *opuCD* | N315 | | 3.10 | probable glycine betaine/carnitine/choline ABC transporter opu | **COG1174E** | **E** | Amino acid transport and metabolism |
| **SA1183** | *opuD* | N315 | 0.30 | 0.32 | glycine betaine transporter | **COG1292M** | **M** | Cell wall/membrane/envelope biogenesis |
| **SA2392** | *panB* | N315 | 0.14 | 0.21 | 3methyl2oxobutanoate hydroxymethyltransferase | **COG0413H** | **H** | Coenzyme transport and metabolism |
| **SA2391** | *panC* | N315 | 0.22 | 0.29 | pantoate betaalanine ligase | | | - |
| **SA1189** | *parC* | N315 | 0.21 | 0.31 | topoisomerase IV subunit A | **COG0188L** | **L** | Replication, recombination and repair |
| **SA1283** | *pbp2* | N315 | 0.15 | 0.17 | PBP2 | **COG0744M** | **M** | Cell wall/membrane/envelope biogenesis |
| **SA1381** | *pbp3* | N315 | 0.23 | 0.31 | penicillinbinding protein 3 | **COG0768M** | **M** | Cell wall/membrane/envelope biogenesis |
| **SA0374** | *pbuX* | N315 | 0.03 | 0.05 | xanthine permease | **COG2233F** | **F** | Nucleotide transport and metabolism |
| **SA2482** | *pcp* | N315 | 5.00 | | pyrrolidonecarboxylate peptidase | **COG2039O** | **O** | Posttranslational modification, protein turnover, chaperones |
| **SA1721** | *pcrA* | N315 | 0.13 | 0.15 | ATPdepentend DNA helicase | **COG0210L** | **L** | Replication, recombination and repair |
| **SA09431** | *pdhA* | N315 | 0.09 | 0.09 | pyruvate dehydrogenase E1 component alpha subunit | **COG1071C** | **C** | Energy production and conversion |
| **SA0944** | *pdhB* | N315 | 0.01 | 0.04 | pyruvate dehydrogenase E1 component beta subunit | **COG0022C** | **C** | Energy production and conversion |
| **SA0945** | *pdhC* | N315 | 0.01 | 0.03 | dihydrolipoamide Sacetyltransferase component of pyruvate de | **COG0508C** | **C** | Energy production and conversion |
| **SA0946** | *pdhD* | N315 | 0.04 | 0.12 | dihydrolipoamide dehydrogenase component of pyruvate dehy | **COG1249C** | **C** | Energy production and conversion |
| **SA1938** | *pdp* | N315 | 0.24 | 0.25 | pyrimidine nucleoside phosphorylase | **COG0213F** | **F** | Nucleotide transport and metabolism |
| **SA0698** | *pepT* | N315 | 3.46 | | aminotripeptidase | **COG2195E** | **E** | Amino acid transport and metabolism |
| **SA1521** | *pfk* | N315 | 0.23 | 0.32 | 6phosphofructokinase | **COG0205G** | **G** | Carbohydrate transport and metabolism |
| **SA0219** | *pflA* | N315 | 0.22 | | formate acetyltransferase activating enzyme | **COG1180O** | **O** | Posttranslational modification, protein turnover, chaperones |
| **SA0218** | *pflB* | N315 | 0.23 | 0.29 | formate acetyltransferase | **COG1882C** | **C** | Energy production and conversion |
| **SA0823** | *pgi* | N315 | 0.02 | 0.04 | glucose6phosphate isomerase A | **COG0166G** | **G** | Carbohydrate transport and metabolism |
| **SA0728** | *pgk* | N315 | 0.06 | 0.16 | phosphoglycerate kinase | | | - |
| **SA0730** | *pgm* | N315 | 0.08 | 0.22 | 2, 3diphosphoglycerateindependentphosphoglycer ate mutase | **COG0696G** | **G** | Carbohydrate transport and metabolism |
| **SA0985** | *pheS* | N315 | 0.13 | 0.19 | PhetRNA synthetase alpha chain | **COG0016J** | **J** | Translation, ribosomal structure and biogenesis |
| **SA0986** | *pheT* | N315 | 0.04 | 0.06 | PhetRNA synthetase beta chain | **COG0072J,COG0073R** | **r** | General function prediction only |
| **SA2420** | *phoB* | N315 | 3.58 | | alkaline phosphatase III precursor | | | - |
| **SA1400** | *phoH* | N315 | 0.18 | 0.21 | phosphate starvationinduced protein phoH homolog | **COG1702T** | **T** | Signal transduction mechanisms |
| **SA0131** | *pnp* | N315 | | 3.05 | purine nucleoside phosphorylase | **COG0813F** | **F** | Nucleotide transport and metabolism |
| **SA1117** | *pnpA* | N315 | 0.15 | 0.18 | polyribonucleotide nucleotidyltransferase | **COG1185J** | **J** | Translation, ribosomal structure and biogenesis |
| **SA1107** | *polC* | N315 | 0.25 | 0.33 | DNA polymerase III, alpha chain PolCtype | **COG2176L** | **L** | Replication, recombination and repair |
| **SA0950** | *potA* | N315 | 0.27 | | spermidine/putrescine ABC transporter, ATPbinding protein ho | **COG3842E** | **E** | Amino acid transport and metabolism |
| **SA0951** | *potB* | N315 | 0.20 | 0.22 | potB | **COG1176E** | **E** | Amino acid transport and metabolism |
| **SA0953** | *potD* | N315 | 0.20 | 0.29 | spermidine/putrescinebinding protein precursor homolog | **COG0687E** | **E** | Amino acid transport and metabolism |
| **SA1920** | *prfA* | N315 | 0.25 | | peptide chain release factor 1 | **COG0216J** | **J** | Translation, ribosomal structure and biogenesis |
| **SA0877** | *prfC* | N315 | 0.11 | 0.27 | peptide chain release factor 3 | **COG4108J** | **J** | Translation, ribosomal structure and biogenesis |
| **SA1055** | *priA* | N315 | 0.22 | 0.19 | PriA, primosomal protein | **COG1198L** | **L** | Replication, recombination and repair |
| **SA1106** | *proS* | N315 | 0.22 | 0.29 | prolinetRNA ligase | **COG0442J** | **J** | Translation, ribosomal structure and biogenesis |
| **SA0458** | *prs* | N315 | 0.32 | 0.26 | ribosephosphate pyrophosphokinase | **COG0462FE** | **e** | Amino acid transport and metabolism |
| **SA1218** | *pstB* | N315 | 3.12 | 4.22 | phosphate ABC transporter, ATPbinding protein | **COG1117P** | **P** | Inorganic ion transport and metabolism |
| **SA1547** | *ptaA* | N315 | 0.19 | 0.23 | PTS system, Nacetylglucosaminespecific IIABC component | **COG1263G** | **G** | Carbohydrate transport and metabolism |
| **SA2326** | *ptsG* | N315 | 0.13 | 0.17 | PTS system, glucosespecific IIABC component | **COG1263G** | **G** | Carbohydrate transport and metabolism |
| **SA0934** | *ptsH* | N315 | 0.09 | 0.13 | phophocarrier protein HPR | **COG1925G** | **G** | Carbohydrate transport and metabolism |
| **SA0935** | *ptsI* | N315 | 0.07 | 0.07 | phosphoenolpyruvateprotein phosphatase | **COG1080G** | **G** | Carbohydrate transport and metabolism |
| **SA1724** | *purB* | N315 | 0.21 | 0.21 | adenylosuccinate lyase | **COG0015F** | **F** | Nucleotide transport and metabolism |
| **SA0918** | *purC* | N315 | 0.04 | 0.04 | phosphoribosylaminoimidazolesuccinocarboxamide synthetase homolog | | | - |
| **SA0926** | *purD* | N315 | 0.01 | 0.01 | phosphoribosylamineglycine ligase PurD | **COG0151F** | **F** | Nucleotide transport and metabolism |
| **SA0922** | *purF* | N315 | 0.04 | 0.06 | phosphoribosylpyrophosphate amidotransferase PurF | **COG0034F** | **F** | Nucleotide transport and metabolism |
| **SA0925** | *purH* | N315 | 0.03 | 0.04 | bifunctional purine biosynthesis protein PurH | | | - |
| **SA0917** | *purK* | N315 | 0.00 | 0.02 | phosphoribosylaminoimidazole carboxylase carbon dioxidefixa | **COG0026F** | **F** | Nucleotide transport and metabolism |
| **SA0921** | *purL* | N315 | 0.01 | 0.02 | phosphoribosylformylglycinamidine synthetase PurL | **COG0046F** | **F** | Nucleotide transport and metabolism |
| **SA0923** | *purM* | N315 | 0.02 | 0.03 | phosphoribosylformylglycinamidine cycloligase PurM | **COG0150F** | **F** | Nucleotide transport and metabolism |
| **SA0924** | *purN* | N315 | 0.03 | 0.04 | phosphoribosylglycinamide formyltransferase | | | - |
| **SA0920** | *purQ* | N315 | 0.01 | 0.02 | phosphoribosylformylglycinamidine synthase I PurQ | **COG0047F** | **F** | Nucleotide transport and metabolism |
| **SA0454** | *purR* | N315 | 0.05 | 0.11 | pur operon repressor homologue | **COG0503F** | **F** | Nucleotide transport and metabolism |
| **SA0963** | *pycA* | N315 | 0.27 | 0.22 | pyruvate carboxylase | **COG1038C** | **C** | Energy production and conversion |
| **SA1520** | *pykA* | N315 | 0.01 | 0.07 | pyruvate kinase | | | - |
| **SA1045** | *pyrAA* | N315 | 0.03 | 0.20 | carbamoylphosphate synthase small chain | **COG0505EF** | **e** | Amino acid transport and metabolism |
| **SA1046** | *pyrAB* | N315 | 0.09 | 0.21 | carbamoylphosphate synthase large chain | **COG0458EF** | **e** | Amino acid transport and metabolism |
| **SA1043** | *pyrB* | N315 | 0.03 | 0.08 | aspartate transcarbamoylase chain A | **COG0540F** | **F** | Nucleotide transport and metabolism |
| **SA1044** | *pyrC* | N315 | 0.03 | 0.10 | dihydroorotase | **COG0044F** | **F** | Nucleotide transport and metabolism |
| **SA1048** | *pyrE* | N315 | 0.01 | 0.03 | orotate phosphoribosyltransferase | **COG0461F** | **F** | Nucleotide transport and metabolism |
| **SA1047** | *pyrF* | N315 | 0.05 | 0.07 | orotidine5phosphate decarboxylase | **COG0284F** | **F** | Nucleotide transport and metabolism |
| **SA1042** | *pyrP* | N315 | 0.08 | 0.24 | uracil permease | **COG2233F** | **F** | Nucleotide transport and metabolism |
| **SA0912** | *qoxB* | N315 | 0.02 | 0.06 | Quinol oxidase polypeptide I QoxB | **COG0843C** | **C** | Energy production and conversion |
| **SA0911** | *qoxC* | N315 | 0.06 | 0.09 | Quinol oxidase polypeptide III QoxC | **COG1845C** | **C** | Energy production and conversion |
| **SA1466** | *queA* | N315 | 0.05 | 0.24 | Sadenosylmethionine tRNA ribosyltransferase | | | - |
| **SA0484** | *radA* | N315 | 0.10 | 0.13 | radA | **COG1066O** | **O** | Posttranslational modification, protein turnover, chaperones |
| **SA0053** | *radC* | N315 | 3.28 | 3.19 | truncated DNA repair protein | **COG2003L** | **L** | Replication, recombination and repair |
| **SA1113** | *rbfA* | N315 | 0.33 | | ribosomebinding factor A | **COG0858J** | **J** | Translation, ribosomal structure and biogenesis |
| **SA0259** | *rbsD* | N315 | 0.27 | 0.22 | ribose permease | **COG1869G** | **G** | Carbohydrate transport and metabolism |
| **SA1128** | *recA* | N315 | 0.27 | 0.32 | RecA protein | **COG0468L** | **L** | Replication, recombination and repair |
| **SA0004** | *recF* | N315 | 0.20 | 0.18 | DNA repair and genetic recombination protein | **COG1195L** | **L** | Replication, recombination and repair |
| **MW1110** | *recG* | MW2 | 0.31 | | ATPdependent DNA helicase | **COG1200LK** | **l** | Replication, recombination and repair |
| **SA1350** | *recN* | N315 | 0.12 | 0.15 | DNA repair protein | **COG0497L** | **L** | Replication, recombination and repair |
| **SA0676** | *recQ* | N315 | 0.26 | 0.30 | probable DNA helicase | **COG0514L** | **L** | Replication, recombination and repair |
| **SA1282** | *recU* | N315 | 0.22 | | recombination protein U homolog | **COG3331R** | **R** | General function prediction only |
| **SA0028** | *repB* | N315 | 4.41 | 3.39 | truncated replication protein for pUB110 plasmid | **COG5655L** | **L** | Replication, recombination and repair |
| **SA1923** | *rho* | N315 | 0.23 | 0.14 | transcription termination factor Rho | **COG1158K** | **K** | Transcription |
| **SA1587** | *ribA* | N315 | 3.44 | | riboflavin biosynthesis protein | **COG0807H** | **H** | Coenzyme transport and metabolism |
| **SA1586** | *ribH* | N315 | 3.13 | 3.12 | 6,7dimethyl8ribityllumazine synthase | | | - |
| **SA1082** | *rimM* | N315 | 0.33 | | probable 16S rRNA processing protein | **COG0806J** | **J** | Translation, ribosomal structure and biogenesis |
| **SA0201** | *rlp* | N315 | 3.83 | 3.52 | RGDcontaining lipoprotein | **COG0747E** | **E** | Amino acid transport and metabolism |
| **SA1324** | *rluB* | N315 | 0.16 | 0.31 | ribosomal large subunit pseudouridine synthase B | **COG1187J** | **J** | Translation, ribosomal structure and biogenesis |
| **SA0735** | *rnr* | N315 | 0.17 | 0.23 | ribonuclease R | **COG0557K** | **K** | Transcription |
| **SA0818** | *rocD* | N315 | 0.27 | 0.16 | ornithine aminotransferase | **COG4992E** | **E** | Amino acid transport and metabolism |
| **SA2044** | *rplB* | N315 | 0.13 | | 50S Protein synthesis L2 | **COG0090J** | **J** | Translation, ribosomal structure and biogenesis |
| **SA2047** | *rplC* | N315 | 0.10 | 0.20 | 50S Protein synthesis L3 | | | - |
| **SA2046** | *rplD* | N315 | 0.20 | 0.20 | 50S Protein synthesis L4 | | | - |
| **SA2035** | *rplE* | N315 | 0.03 | 0.26 | 50S Protein synthesis L5 | **COG0094J** | **J** | Translation, ribosomal structure and biogenesis |
| **SA2033** | *rplF* | N315 | 0.04 | | 50S Protein synthesis L6 | **COG0097J** | **J** | Translation, ribosomal structure and biogenesis |
| **SA0014** | *rplI* | N315 | 0.18 | 0.16 | 50S Protein synthesis L9 | **COG0359J** | **J** | Translation, ribosomal structure and biogenesis |
| **SA0498** | *rplL* | N315 | 0.16 | | 50S Protein synthesis L7/L12 | **COG0222J** | **J** | Translation, ribosomal structure and biogenesis |
| **SA2017** | *rplM* | N315 | 0.14 | 0.22 | 50S Protein synthesis L13 | | | - |
| **SA2037** | *rplN* | N315 | 0.08 | 0.23 | 50S Protein synthesis L14 | | | - |
| **SA2029** | *rplO* | N315 | 0.04 | 0.32 | 50S Protein synthesis L15 | **COG0200J** | **J** | Translation, ribosomal structure and biogenesis |
| **SA2040** | *rplP* | N315 | 0.06 | 0.21 | 50S Protein synthesis L16 | **COG0197J** | **J** | Translation, ribosomal structure and biogenesis |
| **SA2022** | *rplQ* | N315 | 0.12 | | 50S Protein synthesis L17 | **COG0203J** | **J** | Translation, ribosomal structure and biogenesis |
| **SA2032** | *rplR* | N315 | 0.06 | 0.33 | 50S Protein synthesis L18 | **COG0256J** | **J** | Translation, ribosomal structure and biogenesis |
| **SA1473** | *rplU* | N315 | 0.16 | 0.24 | 50S Protein synthesis L21 | | | - |
| **SA2042** | *rplV* | N315 | 0.07 | 0.23 | 50S Protein synthesis L22 | | | - |
| **SA2045** | *rplW* | N315 | 0.15 | 0.23 | 50S Protein synthesis L23 | **COG0089J** | **J** | Translation, ribosomal structure and biogenesis |
| **SA2036** | *rplX* | N315 | 0.04 | 0.22 | 50S Protein synthesis L24 | **COG0198J** | **J** | Translation, ribosomal structure and biogenesis |
| **SA1471** | *rpmA* | N315 | 0.29 | | 50S Protein synthesis L27 | | | - |
| **SA2039** | *rpmC* | N315 | 0.03 | 0.26 | 50S Protein synthesis L29 | | | - |
| **SA2030** | *rpmD* | N315 | 0.05 | | 50S Protein synthesis L30 | **COG1841J** | **J** | Translation, ribosomal structure and biogenesis |
| **SA1922** | *rpmE* | N315 | 3.75 | | Protein synthesis L31 | **COG0254J** | **J** | Translation, ribosomal structure and biogenesis |
| **SAS042** | *rpmG* | N315 | 0.11 | 0.30 | 50S Protein synthesis L33 | **COG0267J** | **J** | Translation, ribosomal structure and biogenesis |
| **SAS078** | *rpmJ* | N315 | 0.01 | 0.17 | 50S Protein synthesis L36 | **COG0257J** | **J** | Translation, ribosomal structure and biogenesis |
| **SA2023** | *rpoA* | N315 | 0.10 | | DNAdirected RNA polymerase alpha chain | **COG0202K** | **K** | Transcription |
| **SA0500** | *rpoB* | N315 | 0.07 | 0.32 | RNA polymerase beta chain | **COG0085K** | **K** | Transcription |
| **SA0501** | *rpoC* | N315 | 0.16 | 0.27 | RNA polymerase betaprime chain | **COG0086K** | **K** | Transcription |
| **SA1099** | *rpsB* | N315 | 0.11 | 0.26 | 30S Protein synthesis S2 | | | - |

| | | | | | | | | |
|---|---|---|---|---|---|---|---|---|
| **SA2041** | *rpsC* | N315 | 0.03 | 0.21 | 30S Protein synthesis S3 | **COG0092J** | **J** | Translation, ribosomal structure and biogenesis |
| **SA2031** | *rpsE* | N315 | 0.14 | 0.30 | 30S Protein synthesis S5 | **COG0098J** | **J** | Translation, ribosomal structure and biogenesis |
| **SA0352** | *rpsF* | N315 | 0.24 | 0.28 | 30S Protein synthesis S6 | **COG0360J** | **J** | Translation, ribosomal structure and biogenesis |
| **SA0504** | *rpsG* | N315 | 0.04 | 0.23 | 30S Protein synthesis S7 | **COG0049J** | **J** | Translation, ribosomal structure and biogenesis |
| **SA2034** | *rpsH* | N315 | 0.03 | 0.25 | 30S Protein synthesis S8 | | | - |
| **SA2016** | *rpsI* | N315 | 0.26 | 0.16 | 30S Protein synthesis S9 | | | - |
| **SA2048** | *rpsJ* | N315 | 0.20 | 0.28 | 30S Protein synthesis S10 | **COG0051J** | **J** | Translation, ribosomal structure and biogenesis |
| **SA2024** | *rpsK* | N315 | 0.21 | 0.26 | 30S Protein synthesis S11 | **COG0100J** | **J** | Translation, ribosomal structure and biogenesis |
| **SA0503** | *rpsL* | N315 | 0.06 | 0.20 | 30S Protein synthesis S12 | | | - |
| **SA2025** | *rpsM* | N315 | 0.01 | 0.12 | 30S Protein synthesis S13 | **COG0099J** | **J** | Translation, ribosomal structure and biogenesis |
| **SAS079** | *rpsN* | N315 | 0.06 | 0.20 | 30S Protein synthesis S14 | **COG0199J** | **J** | Translation, ribosomal structure and biogenesis |
| **SA2038** | *rpsQ* | N315 | 0.03 | 0.19 | 30S Protein synthesis S17 | **COG0186J** | **J** | Translation, ribosomal structure and biogenesis |
| **SA2043** | *rpsS* | N315 | 0.08 | | 30S Protein synthesis S19 | **COG0185J** | **J** | Translation, ribosomal structure and biogenesis |
| **SA1414** | *rpsT* | N315 | 3.12 | | 30S Protein synthesis S20 | **COG0268J** | **J** | Translation, ribosomal structure and biogenesis |
| **SA1872** | *rsbU* | N315 | 0.05 | 0.25 | sigmaB regulation protein RsbU | **COG2208TK** | **t** | Signal transduction mechanisms |
| **SA1871** | *rsbV* | N315 | 0.15 | 0.19 | antisigmaB factor antagonist | **COG1366T** | **T** | Signal transduction mechanisms |
| **SA1870** | *rsbW* | N315 | 0.15 | 0.33 | antisigmaB factor | **COG2172T** | **T** | Signal transduction mechanisms |
| **SA1467** | *ruvB* | N315 | 0.09 | 0.22 | holliday junction DNA helicase | **COG2255L** | **L** | Replication, recombination and repair |
| **SA0010** | SA0010 | N315 | 0.19 | 0.17 | amino acid permease | **COG1296E** | **E** | Amino acid transport and metabolism |
| **SA0036** | SA0036 | N315 | | 3.05 | glycerophosphoryldiester phosphodiesterase homologue | **COG0584C** | **C** | Energy production and conversion |
| **SA0043** | SA0043 | N315 | | 3.61 | hypothetical protein | **COG0491R** | **R** | General function prediction only |
| **SA0062** | SA0062 | N315 | 3.17 | | transposase | **COG2801L** | **L** | Replication, recombination and repair |
| **SA0064** | SA0064 | N315 | | 19.84 | transposase | | | - |
| **SA0077** | SA0077 | N315 | 4.05 | 3.34 | serine/threonine protein kinase | **COG0515RTKL** | **r** | General function prediction only |
| **SA0082** | SA0082 | N315 | | 3.38 | hypothetical protein | **COG0607P,COG2210S,COG0425** | **s** | Function unknown |
| **SA0084** | SA0084 | N315 | 3.03 | 3.01 | homo sapiens CGI44 protein, PRO1975 protein | **COG0446R** | **R** | General function prediction only |
| **SA0089** | SA0089 | N315 | 0.18 | 0.11 | DNA helicase | **COG1112L** | **L** | Replication, recombination and repair |
| **SA0099** | SA0099 | N315 | | 3.30 | transmembrane efflux pump protein | | | - |
| **SA0112** | SA0112 | N315 | 3.06 | 3.47 | cysteine synthase | **COG0031E** | **E** | Amino acid transport and metabolism |
| **SA0113** | SA0113 | N315 | 24.10 | 27.78 | ornithine cyclodeaminase | **COG2423E** | **E** | Amino acid transport and metabolism |
| **SA0117** | SA0117 | N315 | 3.66 | 4.67 | rhizobactin siderophore biosynthesisprotein RhsF | | | - |
| **SA0119** | SA0119 | N315 | | 3.13 | diaminopimelate decarboxylase | **COG0019E** | **E** | Amino acid transport and metabolism |
| **SA0125** | SA0125 | N315 | 3.62 | | Eps(Exopolysaccharide)G | **COG0438M** | **M** | Cell wall/membrane/envelope biogenesis |
| **SA0126** | SA0126 | N315 | 4.35 | 3.56 | capsular polysaccharide synthesis protein 14H | | | - |
| **SA0132** | SA0132 | N315 | | 4.05 | tetracyclin resistance protein | | | - |
| **SA0136** | SA0136 | N315 | 0.25 | 0.29 | phosphonates transport permease | **COG3639P** | **P** | Inorganic ion transport and metabolism |
| **SA0137** | SA0137 | N315 | 0.25 | 0.18 | transport system protein | **COG3638P** | **P** | Inorganic ion transport and metabolism |
| **SA0165** | SA0165 | N315 | 3.44 | 3.03 | alphahelical coiledcoil protein SrpF | | | - |
| **SA0168** | SA0168 | N315 | 3.42 | 3.34 | probable permease of ABC transporter | **COG0600P** | **P** | Inorganic ion transport and metabolism |
| **SA0172** | SA0172 | N315 | 3.36 | | integral membrane protein LmrP | | | - |
| **SA0173** | SA0173 | N315 | 0.13 | 0.29 | surfactin synthetase | **COG3320Q,COG1020Q** | **Q** | Secondary metabolites biosynthesis, transport and catabolism |
| **SA0180** | SA0180 | N315 | | 0.30 | branchedchain amino acid transport system carrier protein | **COG1114E** | **E** | Amino acid transport and metabolism |
| **SA0181** | SA0181 | N315 | 0.31 | | isochorismatase | **COG1335Q** | **Q** | Secondary metabolites biosynthesis, transport and catabolism |
| **SA0193** | SA0193 | N315 | 7.09 | 6.54 | Enterococcus faecalis plasmid pPD1 bacI | **COG0577V** | **V** | Defense mechanisms |
| **SA0207** | SA0207 | N315 | 4.42 | | maltose/maltodextrinbinding protein | **COG2182G** | **G** | Carbohydrate transport and metabolism |
| **SA0211** | SA0211 | N315 | 6.33 | | NADHdependent dehydrogenase | **COG0673R** | **R** | General function prediction only |
| **SA0215** | SA0215 | N315 | 3.60 | | twocomponent response regulator | **COG4753T** | **T** | Signal transduction mechanisms |
| **SA0220** | SA0220 | N315 | 3.47 | 3.11 | glycerophosphodiester phosphodiesterase | **COG0584C,COG4781C** | **C** | Energy production and conversion |
| **SA0231** | SA0231 | N315 | 0.33 | | flavohemoprotein | **COG1017C,COG1018C** | **C** | Energy production and conversion |
| **SA0234** | SA0234 | N315 | 3.76 | 3.55 | inosineuridine preferring nucleoside hydrolase | **COG1957F** | **F** | Nucleotide transport and metabolism |
| **SA0241** | SA0241 | N315 | 0.16 | 0.15 | 4diphosphocytidyl2CmethylDerythritol synthase | **COG1211I** | **I** | Lipid transport and metabolism |
| **SA0242** | SA0242 | N315 | 0.04 | 0.04 | xylitol dehydrogenase | **COG1063ER** | **e** | Amino acid transport and metabolism |
| **SA0243** | SA0243 | N315 | 0.11 | | teichoic acid biosynthesis protein B | **COG1887M** | **M** | Cell wall/membrane/envelope biogenesis |
| **SA0246** | SA0246 | N315 | 0.08 | 0.18 | hypotheticl protein, similar to Dxylulose reductase | **COG1063ER** | **e** | Amino acid transport and metabolism |
| **SA0247** | SA0247 | N315 | | 0.09 | teichoic acid biosynthesis protein B | **COG1887M** | **M** | Cell wall/membrane/envelope biogenesis |
| **SA0248** | SA0248 | N315 | 0.13 | 0.17 | betaglycosyltransferase | **COG0463M** | **M** | Cell wall/membrane/envelope biogenesis |
| **SA0255** | SA0255 | N315 | 3.24 | | PTS betaglucosidespecific enzyme II, ABC component | | | - |
| **SA0263** | SA0263 | N315 | 4.35 | 3.55 | proton antiporter efflux pump | | | - |
| **SA0266** | SA0266 | N315 | | 0.27 | hypothetical protein | **COG1131V** | **V** | Defense mechanisms |
| **SA0276** | SA0276 | N315 | 0.28 | 0.23 | Cdiarrheal toxin | **COG1674D** | **D** | Cell cycle control, cell division, chromosome partitioning |
| **SA0299** | SA0299 | N315 | 3.53 | 3.60 | carbohydrate kinase, PfkB family | **COG0524G** | **G** | Carbohydrate transport and metabolism |
| **SA0303** | SA0303 | N315 | 3.08 | | sodiumcoupled permease | **COG0591ER** | **e** | Amino acid transport and metabolism |
| **SA0312** | SA0312 | N315 | 0.33 | | alkanal monooxygenase alpha chain | **COG2141C** | **C** | Energy production and conversion |
| **SA0313** | SA0313 | N315 | 0.29 | 0.20 | glycine cleavage system H protein | **COG0509E** | **E** | Amino acid transport and metabolism |
| **SA0316** | SA0316 | N315 | 0.33 | | lipoateprotein ligase | **COG0095H** | **H** | Coenzyme transport and metabolism |
| **SA0328** | SA0328 | N315 | 3.10 | | NADHdependent FMN reductase | **COG0431R** | **R** | General function prediction only |
| **SA0330** | SA0330 | N315 | 4.78 | 4.41 | ribosomalproteinserine Nacetyltransferase | **COG1670J** | **J** | Translation, ribosomal structure and biogenesis |
| **SA0337** | SA0337 | N315 | 3.91 | | transcriptional repressor | **COG1476K** | **K** | Transcription |
| **SA0341** | SA0341 | N315 | 3.82 | | low temperature requirement A protein | | | - |
| **SA0355** | SA0355 | N315 | 4.02 | | HP virulence plasmid pXO138 | | | - |
| **SA0361** | SA0361 | N315 | 4.22 | 5.26 | truncated phosphoglycerate mutase; Gpm3p | **COG0406G** | **G** | Carbohydrate transport and metabolism |
| **SA0417** | SA0417 | N315 | 3.04 | | sodiumdependent transporter | **COG0733R** | **R** | General function prediction only |
| **SA0422** | SA0422 | N315 | 3.69 | 3.64 | lactococcal lipoprotein | **COG1464P** | **P** | Inorganic ion transport and metabolism |
| **SA0453** | SA0453 | N315 | 0.13 | 0.18 | 4diphosphocytidyl2CmethylDerythritol kinase | **COG1947I** | **I** | Lipid transport and metabolism |
| **SA0462** | SA0462 | N315 | 0.01 | 0.13 | low temperature requirement B protein | **COG2244R** | **R** | General function prediction only |
| **SA0463** | SA0463 | N315 | | 0.32 | hypothetical protein | **COG3956R** | **R** | General function prediction only |
| **SA0465** | SA0465 | N315 | 0.18 | 0.26 | celldivision initiation protein | **COG2919D** | **D** | Cell cycle control, cell division, chromosome partitioning |
| **SA0466** | SA0466 | N315 | | 0.31 | polyribonucleotide nucleotidyltransferase | **COG1098J** | **J** | Translation, ribosomal structure and biogenesis |
| **SA0468** | SA0468 | N315 | 0.09 | 0.17 | hypoxanthineguanine phosphoribosyltransferase homologue | **COG0634F** | **F** | Nucleotide transport and metabolism |
| **SA0476** | SA0476 | N315 | 4.69 | 3.13 | transcription regulator GntR family | **COG1167KE** | **e** | Amino acid transport and metabolism |
| **SA0482** | SA0482 | N315 | 0.29 | | creatine kinase | **COG3869E** | **E** | Amino acid transport and metabolism |
| **SA0492** | SA0492 | N315 | 0.07 | | HP | **COG1595K** | **K** | Transcription |
| **SA0502** | SA0502 | N315 | 0.06 | 0.30 | Protein synthesis | **COG1358J** | **J** | Translation, ribosomal structure and biogenesis |
| **SA0528** | SA0528 | N315 | 0.07 | 0.06 | hexulose6phosphate synthase | **COG0269G** | **G** | Carbohydrate transport and metabolism |
| **SA0557** | SA0557 | N315 | | 0.24 | oxidoreductase, ion channel | **COG0667C** | **C** | Energy production and conversion |
| **SA0565** | SA0565 | N315 | 3.04 | | endonuclease III | **COG2231L** | **L** | Replication, recombination and repair |
| **SA0567** | SA0567 | N315 | 5.00 | | iron(III) ABC transporter permease protein | **COG0609P** | **P** | Inorganic ion transport and metabolism |
| **SA0569** | SA0569 | N315 | 0.16 | | 2hydroxy6oxo6phenylhexa2,4dienoic acid hydrolase | **COG0596R** | **R** | General function prediction only |
| **SA0572** | SA0572 | N315 | 3.31 | 3.31 | esterase/lipase | **COG0596R** | **R** | General function prediction only |
| **SA0580** | SA0580 | N315 | 0.18 | | Na+/H+ antiporter | **COG1006P** | **P** | Inorganic ion transport and metabolism |
| **SA0587** | SA0587 | N315 | 0.08 | 0.10 | lipoprotein, Streptococcal adhesin PsaA homologue | **COG0803P** | **P** | Inorganic ion transport and metabolism |
| **SA0589** | SA0589 | N315 | 0.15 | | ABC transporter ATPbinding protein | **COG1121P** | **P** | Inorganic ion transport and metabolism |
| **SA0590** | SA0590 | N315 | 4.50 | 3.47 | iron dependent repressor | **COG1321K** | **K** | Transcription |
| **SA0600** | SA0600 | N315 | | 0.33 | pyrimidine nucleoside transporter | | | - |
| **SA0605** | SA0605 | N315 | 0.24 | 0.27 | dihydroxyacetone kinase | **COG2376G** | **G** | Carbohydrate transport and metabolism |
| **SA0614** | SA0614 | N315 | | 0.22 | twocomponent response regulator | **COG0745TK** | **t** | Signal transduction mechanisms |
| **SA0622** | SA0622 | N315 | 5.32 | | AraC/XylS family transcriptional regulator | | | - |
| **SA0628** | SA0628 | N315 | 0.25 | 0.30 | sugar efflux transpoter | | | - |
| **SA0640** | SA0640 | N315 | 0.33 | 0.21 | ABC transporter required for expression of cytochrome bd | **COG4987CO** | **c** | Energy production and conversion |
| **SA0646** | SA0646 | N315 | 0.18 | 0.17 | deoxyribodipyrimidine photolyase | **COG0415L** | **L** | Replication, recombination and repair |
| **SA0670** | SA0670 | N315 | 0.10 | 0.29 | paraaminobenzoate synthase component I | **COG0115EH** | **e** | Amino acid transport and metabolism |
| **SA0672** | SA0672 | N315 | 0.09 | 0.12 | urea amidolyase | | | - |
| **SA0674** | SA0674 | N315 | 0.12 | 0.20 | anionbinding protein | **COG1368M** | **M** | Cell wall/membrane/envelope biogenesis |
| **SA0677** | SA0677 | N315 | 0.31 | | choline transport ATPbinding protein | **COG1125E** | **E** | Amino acid transport and metabolism |
| **SA0678** | SA0678 | N315 | 0.22 | 0.25 | choline transporter | **COG1174E,COG1732M** | **E** | Amino acid transport and metabolism |
| **SA0679** | SA0679 | N315 | 0.25 | 0.17 | histidinolphosphate aminotransferase | **COG0079E** | **E** | Amino acid transport and metabolism |
| **SA0682** | SA0682 | N315 | 0.25 | 0.14 | ditripepride ABC transporter | **COG3104E** | **E** | Amino acid transport and metabolism |
| **SA0683** | SA0683 | N315 | | 0.24 | hypothetical protein | **COG0780R** | **R** | General function prediction only |
| **SA0688** | SA0688 | N315 | 0.26 | | ferrichrome ABC transporter permease | **COG4606P** | **P** | Inorganic ion transport and metabolism |
| **SA0690** | SA0690 | N315 | 0.28 | 0.24 | ferrichrome ABC transporter ATPbinding protein | **COG4604P** | **P** | Inorganic ion transport and metabolism |
| **SA0693** | SA0693 | N315 | 0.33 | | UDPNacetylenolpyruvoylglucosamine reductase | **COG0812M** | **M** | Cell wall/membrane/envelope biogenesis |
| **SA0705** | SA0705 | N315 | 3.80 | | comF operon protein 1 | **COG4098L** | **L** | Replication, recombination and repair |
| **SA0724** | SA0724 | N315 | 3.29 | 3.11 | celldivision inhibitor | **COG1090R** | **R** | General function prediction only |
| **SA0734** | SA0734 | N315 | 0.31 | | carboxyesterase precursor homologue | **COG1647R** | **R** | General function prediction only |
| **SA0746** | SA0746 | N315 | | 3.64 | staphylococcal nuclease | **COG1525L** | **L** | Replication, recombination and repair |
| **SA0759** | SA0759 | N315 | 3.39 | | arsenate reductase | **COG1393P** | **P** | Inorganic ion transport and metabolism |
| **SA0776** | SA0776 | N315 | 0.26 | | aminotransferase NifS homologue | **COG0520E** | **E** | Amino acid transport and metabolism |
| **SA0777** | SA0777 | N315 | 0.10 | 0.19 | nitrogen fixation protein NifU | **COG0822C** | **C** | Energy production and conversion |
| **SA0790** | SA0790 | N315 | 0.12 | 0.17 | Nacetylglucosamine catabolism homologue | **COG0647G** | **G** | Carbohydrate transport and metabolism |
| **SA0791** | SA0791 | N315 | 0.25 | 0.20 | glycerate dehydrogenase | **COG1052CHR** | **h** | Coenzyme transport and metabolism |
| **SA0827** | SA0827 | N315 | 0.06 | 0.10 | ATPdependent nuclease subunit B | **COG3857L** | **L** | Replication, recombination and repair |
| **SA0828** | SA0828 | N315 | 0.19 | 0.26 | ATPdependent nuclease subunit A | **COG1074L** | **L** | Replication, recombination and repair |
| **SA0829** | SA0829 | N315 | 0.28 | 0.26 | 5oxo1,2,5tricarboxilic3penten acid decarboxylase | **COG0179Q** | **Q** | Secondary metabolites biosynthesis, transport and catabolism |
| **SA0846** | SA0846 | N315 | 0.01 | 0.01 | oligopeptide transport system permease protein OppC | **COG1173EP** | **e** | Amino acid transport and metabolism |
| **SA0849** | SA0849 | N315 | 0.02 | 0.02 | peptide binding protein OppA | **COG4166E** | **E** | Amino acid transport and metabolism |
| **SA0851** | SA0851 | N315 | 5.26 | 4.72 | oligopeptide ABC transporter ATPbinding protein homologue | **COG0444EP** | **e** | Amino acid transport and metabolism |
| **SA0857** | SA0857 | N315 | 0.20 | 0.31 | negative regulator of genetic competence MecA | | | - |
| **SA0859** | SA0859 | N315 | 0.28 | 0.22 | thimet oligopeptidase homologue | **COG1164E** | **E** | Amino acid transport and metabolism |
| **SA0863** | SA0863 | N315 | | 0.33 | hypothetical protein | | | - |
| **SA0864** | SA0864 | N315 | 0.17 | 0.24 | GTP pyrophosphokinase | **COG2357S** | **S** | Function unknown |
| **SA0880** | SA0880 | N315 | 0.05 | 0.04 | Na+transporting ATP synthase | **COG0168P** | **P** | Inorganic ion transport and metabolism |
| **SA0881** | SA0881 | N315 | 3.76 | 3.57 | nucleotidase | **COG0737F** | **F** | Nucleotide transport and metabolism |
| **SA0882** | SA0882 | N315 | 3.77 | 3.08 | competence transcription factor | | | - |
| **SA0895** | SA0895 | N315 | 3.40 | 4.07 | menaquinonespecific isochorismate synthase | **COG1169HQ** | **h** | Coenzyme transport and metabolism |
| **SA0910** | SA0910 | N315 | 0.05 | 0.13 | quinol oxidase polypeptide IV QoxD | **COG3125C** | **C** | Energy production and conversion |
| **SA0913** | SA0913 | N315 | 0.05 | 0.10 | quinol oxidase polypeptide II QoxA | **COG1622C** | **C** | Energy production and conversion |
| **SA0916** | SA0916 | N315 | 0.10 | 0.10 | phosphoribosylaminoimidazole carboxylase PurE | **COG0041F** | **F** | Nucleotide transport and metabolism |
| **SA0947** | SA0947 | N315 | | 0.28 | hypothetical protein | | | - |
| **SA0956** | SA0956 | N315 | 0.28 | 0.32 | Mn2+transport protein | **COG1914P** | **P** | Inorganic ion transport and metabolism |
| **SA0969** | SA0969 | N315 | | 3.31 | glycerophosphoryl diester phosphodiesterase | **COG0584C** | **C** | Energy production and conversion |
| **SA0990** | SA0990 | N315 | 0.25 | | DNAdependent DNA polymerase beta chain | **COG1796L,COG1387ER** | **e** | Amino acid transport and metabolism |
| **SA1009** | SA1009 | N315 | 3.73 | | exotoxin 1 | | | - |
| **SA1011** | SA1011 | N315 | 5.00 | 4.69 | exotoxin 3 | | | - |
| **SA1058** | SA1058 | N315 | 0.32 | | polypeptide deformylase | | | - |
| **SA1059** | SA1059 | N315 | 0.23 | | methionyltRNA formyltransferase | **COG0223J** | **J** | Translation, ribosomal structure and biogenesis |
| **SA1063** | SA1063 | N315 | 0.31 | | protein kinase | **COG2815S,COG0515RTKL** | **S** | Function unknown |
| **SA1078** | SA1078 | N315 | 0.08 | 0.12 | signal recognition particle | **COG0552U** | **U** | Intracellular trafficking, secretion, and vesicular transport |
| **SA1100** | SA1100 | N315 | 0.26 | | elongation factor TS | | | - |
| **SA1111** | SA1111 | N315 | 0.07 | 0.10 | Protein synthesis L7AE family | **COG1358J** | **J** | Translation, ribosomal structure and biogenesis |

| | | | | | | | | |
|---|---|---|---|---|---|---|---|---|
| SA1122 | SA1122 | N315 | 0.26 | | precessing proteinase | COG0612R | R | General function prediction only |
| SA1123 | SA1123 | N315 | 0.04 | 0.10 | 3oxoacyl acylcarrier protein reductase homolog ymfI | COG1028IQR | r | General function prediction only |
| SA1131 | SA1131 | N315 | 0.07 | 0.25 | 2oxoacid ferredoxin oxidoreductase, alpha subunit | COG1014C,COG0674C | C | Energy production and conversion |
| SA1132 | SA1132 | N315 | 0.12 | 0.18 | 2oxoacid ferredoxin oxidoreductase, beta subunit | COG1013C | C | Energy production and conversion |
| SA1143 | SA1143 | N315 | 4.15 | | lysophospholipase | COG2267I | I | Lipid transport and metabolism |
| SA1169 | SA1169 | N315 | 0.24 | 0.21 | gammaaminobutyrate permease | COG0833E | E | Amino acid transport and metabolism |
| SA1172 | SA1172 | N315 | 0.16 | 0.27 | GMP reductase | COG0516F | F | Nucleotide transport and metabolism |
| SA1180 | SA1180 | N315 | 5.00 | 5.10 | exonuclease SbcD | COG0420L | L | Replication, recombination and repair |
| SA1216 | SA1216 | N315 | | 0.24 | oligoendopeptidase | COG1164E | E | Amino acid transport and metabolism |
| SA1220 | SA1220 | N315 | | 3.03 | phosphate ABC transporter | COG0573P | P | Inorganic ion transport and metabolism |
| SA1231 | SA1231 | N315 | | 0.10 | alanine racemase | COG0787M | M | Cell wall/membrane/envelope biogenesis |
| SA1237 | SA1237 | N315 | 0.16 | 0.15 | 5bromo4chloroindolyl phosphate hydrolysis protein xpaC | | | - |
| SA1238 | SA1238 | N315 | 0.23 | 0.20 | tellurite resistance protein | COG3853P | P | Inorganic ion transport and metabolism |
| SA1241 | SA1241 | N315 | 0.25 | 0.29 | nitricoxide reductase | COG0714R | R | General function prediction only |
| SA1252 | SA1252 | N315 | | 0.28 | hypothetical protein | | | - |
| SA1255 | SA1255 | N315 | 0.09 | 0.25 | PTS system, glucosespecific enzyme II, A component | | | - |
| SA1257 | SA1257 | N315 | 0.11 | 0.10 | peptide methionine sulfoxide reductase | | | - |
| SA1270 | SA1270 | N315 | 3.70 | 3.64 | amino acid pearmease | COG0531E | E | Amino acid transport and metabolism |
| SA1273 | SA1273 | N315 | 0.15 | 0.15 | 5'3' exonuclease | COG0258L | L | Replication, recombination and repair |
| SA1280 | SA1280 | N315 | | 0.32 | conserved hyothetical protein | | | - |
| SA1289 | SA1289 | N315 | 0.32 | 0.33 | bifunctional biotin ligase/biotin operon repressor | COG0340H,COG1654K | H | Coenzyme transport and metabolism |
| SA1290 | SA1290 | N315 | 0.09 | 0.33 | poly(A) polymerase | COG0617J | J | Translation, ribosomal structure and biogenesis |
| SA1291 | SA1291 | N315 | | 0.30 | lipopolysaccharide biosynthesisrelated pr homolog | COG0438M | M | Cell wall/membrane/envelope biogenesis |
| SA1307 | SA1307 | N315 | 0.13 | 0.31 | GTP binding protein | COG1160R | R | General function prediction only |
| SA1329 | SA1329 | N315 | 0.22 | 0.26 | ferric uptake regulator homolog | COG0735P | P | Inorganic ion transport and metabolism |
| SA1336 | SA1336 | N315 | 0.14 | 0.16 | glucose6phosphate 1dehydrogenase | | | - |
| SA1345 | SA1345 | N315 | | 0.24 | hypothetical protein | | | - |
| SA1349 | SA1349 | N315 | 0.18 | 0.21 | dihydrolipoamide dehydrogenase | COG1249C | C | Energy production and conversion |
| SA1353 | SA1353 | N315 | 0.26 | 0.31 | exodeoxyribonuclease, small subunit | COG1722L | L | Replication, recombination and repair |
| SA1354 | SA1354 | N315 | 0.17 | 0.33 | exodeoxyribonuclease large subunit | COG1570L | L | Replication, recombination and repair |
| SA1355 | SA1355 | N315 | 0.08 | 0.14 | transcription termination factor | COG0781K | K | Transcription |
| SA1365 | SA1365 | N315 | 0.06 | 0.08 | glycine dehydrogenase (decarboxylating) subunit 2 homolog | COG1003E | E | Amino acid transport and metabolism |
| SA1366 | SA1366 | N315 | 0.10 | 0.12 | glycine dehydrogenase subunit 1 | COG0403E | E | Amino acid transport and metabolism |
| SA1367 | SA1367 | N315 | 0.09 | 0.12 | aminomethyltransferase | COG0404E | E | Amino acid transport and metabolism |
| SA1374 | SA1374 | N315 | 3.08 | | late competence protein comGA | COG2804NU | n | Cell motility |
| SA1384 | SA1384 | N315 | 0.12 | | ABC transporter | COG1108P | P | Inorganic ion transport and metabolism |
| SA1385 | SA1385 | N315 | | 0.30 | ABC transporter ATPbinding protein | COG1121P | P | Inorganic ion transport and metabolism |
| SA1386 | SA1386 | N315 | 0.04 | 0.20 | endonuclease IV | COG0648L | L | Replication, recombination and repair |
| SA1387 | SA1387 | N315 | 0.15 | | ATPdependent RNA helicase | COG0513LKJ | l | Replication, recombination and repair |
| SA1398 | SA1398 | N315 | 0.09 | 0.09 | diacylglycerol kinase | | | - |
| SA1435 | SA1435 | N315 | 4.48 | 3.58 | acetylCoA carboxylase (biotin carboxyl carrier subunit), accB h | COG0511I | I | Lipid transport and metabolism |
| SA1441 | SA1441 | N315 | 0.04 | 0.16 | protease | | | - |
| SA1449 | SA1449 | N315 | 3.40 | | (5methylaminomethyl2thiouridylate)methyl transferase | COG0482J | J | Translation, ribosomal structure and biogenesis |
| SA1462 | SA1462 | N315 | 0.23 | 0.33 | singlestrand DNAspecific exonuclease | COG0608L | L | Replication, recombination and repair |
| SA1469 | SA1469 | N315 | 0.09 | 0.28 | chorismate mutase | COG4492R | R | General function prediction only |
| SA1512 | SA1512 | N315 | 0.11 | 0.13 | formamidopyrimidineDNA glycosylase | COG0266L | L | Replication, recombination and repair |
| SA1523 | SA1523 | N315 | 0.11 | 0.11 | acetylCoA carboxylase transferase beta subunit | COG0777I | I | Lipid transport and metabolism |
| SA1524 | SA1524 | N315 | 0.24 | 0.32 | malate dehydrogenase homolog | COG0281C | C | Energy production and conversion |
| SA1529 | SA1529 | N315 | | 3.57 | hypothetical protein | COG2220R | R | General function prediction only |
| SA1537 | SA1537 | N315 | 0.01 | 0.12 | thiamine biosynthesis protein ThiI | COG0301H | H | Coenzyme transport and metabolism |
| SA1538 | SA1538 | N315 | 0.24 | 0.23 | ironsulfur cofactor synthesis protein nifZ | COG1104E | E | Amino acid transport and metabolism |
| SA1540 | SA1540 | N315 | | 3.14 | hypothetical protein | COG1956T | T | Signal transduction mechanisms |
| SA1559 | SA1559 | N315 | 0.19 | 0.20 | smooth muscle caldesmon | | | - |
| SA1560 | SA1560 | N315 | 0.13 | 0.18 | general stress protein homolog | COG4768R | R | General function prediction only |
| SA1571 | SA1571 | N315 | 0.26 | 0.24 | Dalanine aminotransferase | COG0115EH | e | Amino acid transport and metabolism |
| SA1572 | SA1572 | N315 | 0.03 | 0.03 | XaaHis dipeptidase homolog | COG0624E | E | Amino acid transport and metabolism |
| SA1574 | SA1574 | N315 | 0.14 | 0.11 | 16S pseudouridylate synthase | COG1187J | J | Translation, ribosomal structure and biogenesis |
| SA1575 | SA1575 | N315 | 0.28 | | spore cortex protein homolog | COG2244R | R | General function prediction only |
| SA1584 | SA1584 | N315 | 4.20 | 3.39 | lysophospholipase homolog | COG2267I | I | Lipid transport and metabolism |
| SA1592 | SA1592 | N315 | 0.25 | 0.16 | aesenical pump membrane protein homolog | COG1055P | P | Inorganic ion transport and metabolism |
| SA1617 | SA1617 | N315 | 0.16 | 0.15 | latent nuclear antigen [Kaposi's sarcomaassociated herpesvirus] | | | - |
| SA1633 | SA1633 | N315 | 16.08 | 18.76 | probable betalactamase | | | - |
| SA1667 | SA1667 | N315 | 0.11 | 0.23 | twocomponent sensor histidine kinase | COG2203T,COG4585T | T | Signal transduction mechanisms |
| SA1678 | SA1678 | N315 | 3.50 | 3.03 | transcription regulator Fur family homolog | COG0735P | P | Inorganic ion transport and metabolism |
| SA1688 | SA1688 | N315 | 0.17 | 0.33 | teichoic acid translocation ATPbinding protein tagH | COG1134GM | g | Carbohydrate transport and metabolism |
| SA1708 | SA1708 | N315 | 0.24 | 0.20 | UDPNacetylmuramyl tripeptide synthetase homolog | COG0769M | M | Cell wall/membrane/envelope biogenesis |
| SA1713 | SA1713 | N315 | 0.22 | 0.19 | RNA methyltransferase homolog | COG2265J | J | Translation, ribosomal structure and biogenesis |
| SA1715 | SA1715 | N315 | 0.17 | 0.16 | glutamyltRNAGln amidotransferase subunit B | COG0064J | J | Translation, ribosomal structure and biogenesis |
| SA1716 | SA1716 | N315 | 0.07 | 0.08 | glutamyltRNAGln amidotransferase subunit A | | | - |
| SA1717 | SA1717 | N315 | | 0.28 | glutamyltRNAGln amidotransferase subunit C | COG0721J | J | Translation, ribosomal structure and biogenesis |
| SA1719 | SA1719 | N315 | | 0.30 | hypothetical protein | | | - |
| SA1725 | SA1725 | N315 | 0.27 | 0.21 | Staphopain, Cysteine Proteinase | | | - |
| SA1729 | SA1729 | N315 | 0.01 | 0.04 | nicotinate phosphoribosyltransferase homolog | COG1488H | H | Coenzyme transport and metabolism |
| SA1730 | SA1730 | N315 | | 0.28 | nitricoxide synthase homolog | COG4362PE | e | Amino acid transport and metabolism |
| SA1735 | SA1735 | N315 | 0.12 | 0.14 | manganesedependent inorganic pyrophosphatase | COG1227C | C | Energy production and conversion |
| SA1747 | SA1747 | N315 | 0.31 | | ABC transporter, ATPbinding protein | COG1131V | V | Defense mechanisms |
| SA1748 | SA1748 | N315 | 0.12 | | transcription regulator, GntR family | COG1725K | K | Transcription |
| SA1749 | SA1749 | N315 | 3.22 | | aspartate transaminase protein | COG0436E | E | Amino acid transport and metabolism |
| SA1759 | SA1759 | N315 | 4.50 | | lytic enzyme | | | - |
| SA1760 | SA1760 | N315 | | 3.82 | holin homolog | | | - |
| SA1775 | SA1775 | N315 | 3.04 | 3.06 | scaffolding protein | COG0740OU | u | Intracellular trafficking, secretion, and vesicular transport |
| SA1806 | SA1806 | N315 | 4.03 | 3.80 | probable ATPdependent helicase | COG0847L | L | Replication, recombination and repair |
| SA1812 | SA1812 | N315 | 0.14 | 0.22 | synergohymenotropic toxin precursor Staphylococcus intermedius | | | - |
| SA1813 | SA1813 | N315 | 0.27 | 0.33 | leukocidin chain lukM precursor | | | - |
| SA1814 | SA1814 | N315 | 3.57 | | succinyldiaminopimelate desuccinylase | COG0624E | E | Amino acid transport and metabolism |
| SA1820 | SA1820 | N315 | | 3.01 | bacteriophage terminase small subunit | COG3728L | L | Replication, recombination and repair |
| SA1839 | SA1839 | N315 | 0.13 | 0.24 | SdrH | | | - |
| SA1854 | SA1854 | N315 | 0.22 | | Osialoglycoprotein endopeptidase | COG0533O | O | Posttranslational modification, protein turnover, chaperones |
| SA1855 | SA1855 | N315 | 0.15 | 0.20 | ribosomalproteinalanine Nacetyltransfer | COG0456R | R | General function prediction only |
| SA1885 | SA1885 | N315 | | 0.32 | ATPdependent RNA helicase | COG0513LKJ | l | Replication, recombination and repair |
| SA1888 | SA1888 | N315 | 4.52 | | rod shape determining protein RodA | | | - |
| SA1893 | SA1893 | N315 | 3.14 | 3.52 | lipoprotein precursor | COG0706U | U | Intracellular trafficking, secretion, and vesicular transport |
| SA1919 | SA1919 | N315 | 0.05 | 0.10 | protoporphyrinogen oxidase (hemK) | COG2890J | J | Translation, ribosomal structure and biogenesis |
| SA1939 | SA1939 | N315 | 0.15 | 0.25 | deoxyribosephosphate aldolase | COG0274F | F | Nucleotide transport and metabolism |
| SA1958 | SA1958 | N315 | | 3.12 | transposase for IS232 | COG1119P | P | Inorganic ion transport and metabolism |
| SA1972 | SA1972 | N315 | 3.14 | 3.28 | multidrug transporter | | | - |
| SA1975 | SA1975 | N315 | | 0.24 | conserved hypotehtical protein | COG1988R | R | General function prediction only |
| SA1978 | SA1978 | N315 | 0.33 | 0.31 | ferrichrome ABC transporter (permease) | COG0609P | P | Inorganic ion transport and metabolism |
| SA1979 | SA1979 | N315 | 0.30 | 0.26 | HP, similar toferrichrome ABC transporter (binding prote) | COG4594P | P | Inorganic ion transport and metabolism |
| SA1989 | SA1989 | N315 | 0.22 | 0.21 | quinone oxidoreductase | COG2130R | R | General function prediction only |
| SA2002 | SA2002 | N315 | 3.16 | 4.33 | transcription regulator MerR family | COG0789K | K | Transcription |
| SA2006 | SA2006 | N315 | 7.75 | 11.20 | MHC class II analog | | | - |
| SA2010 | SA2010 | N315 | 4.83 | | RNAdirected DNA polymerase from retron EC86 | COG3344L | L | Replication, recombination and repair |
| SA2056 | SA2056 | N315 | 0.12 | 0.13 | acriflavin resistance protein | COG0841V | V | Defense mechanisms |
| SA2077 | SA2077 | N315 | 0.05 | 0.06 | biotin biosynthesis protein | COG1268R | R | General function prediction only |
| SA2081 | SA2081 | N315 | 3.02 | 4.18 | urea transporter | COG4413E | E | Amino acid transport and metabolism |
| SA2102 | SA2102 | N315 | 0.28 | 0.31 | formate dehydrogenase homolog | COG3383R | R | General function prediction only |
| SA2106 | SA2106 | N315 | 3.48 | 4.33 | protein of pXO246 | | | - |
| SA2108 | SA2108 | N315 | 0.28 | | transcription regulator, RpiR family | COG1737K | K | Transcription |
| SA2112 | SA2112 | N315 | | 3.64 | sodiumdependent transporter | COG0385R | R | General function prediction only |
| SA2125 | SA2125 | N315 | 0.13 | 0.14 | formiminoglutamase | COG0010E | E | Amino acid transport and metabolism |
| SA2127 | SA2127 | N315 | 3.13 | 4.10 | ribose 5phosphate isomerase (rpi) | COG0120G | G | Carbohydrate transport and metabolism |
| SA2129 | SA2129 | N315 | 0.06 | 0.04 | aldose 1epimerase | COG2017G | G | Carbohydrate transport and metabolism |
| SA2137 | SA2137 | N315 | 0.08 | 0.31 | divalent cation transport | COG0598P | P | Inorganic ion transport and metabolism |
| SA2140 | SA2140 | N315 | | 0.25 | esterase | COG0657I | I | Lipid transport and metabolism |
| SA2142 | SA2142 | N315 | 0.15 | 0.16 | multidrug resistance protein | | | - |
| SA2149 | SA2149 | N315 | | 4.39 | ABC transporter, ATPbinding protein | COG1136V | V | Defense mechanisms |
| SA2158 | SA2158 | N315 | 0.11 | 0.10 | TpgX protein | | | - |
| SA2164 | SA2164 | N315 | 3.86 | 3.88 | phage infection protein precursor | COG1511S | S | Function unknown |
| SA2165 | SA2165 | N315 | 0.15 | 0.18 | transcriptional regulator tetRfamily | COG1309K | K | Transcription |
| SA2169 | SA2169 | N315 | 0.15 | 0.27 | transcription regulatory protein | | | - |
| SA2174 | SA2174 | N315 | 3.34 | 4.29 | transcriptional regulator | COG1846K | K | Transcription |
| SA2179 | SA2179 | N315 | 0.20 | 0.22 | response regulators of twocomponent regulatory | COG2197TK | t | Signal transduction mechanisms |
| SA2180 | SA2180 | N315 | 0.01 | 0.07 | two component sensor histidine kinase | COG4585T,COG0642T | T | Signal transduction mechanisms |
| SA2200 | SA2200 | N315 | 0.31 | | ABC transporter, ATP binding subunit | COG1126E | E | Amino acid transport and metabolism |
| SA2201 | SA2201 | N315 | 0.20 | 0.24 | ABC transporter, permease protein | COG0765E | E | Amino acid transport and metabolism |
| SA2212 | SA2212 | N315 | 0.05 | 0.28 | 8amino7oxononanoate synthase | COG0156H | H | Coenzyme transport and metabolism |
| SA2217 | SA2217 | N315 | 3.13 | 3.15 | lipoprotein inner membrane ABCtransporter | COG1132V | V | Defense mechanisms |
| SA2228 | SA2228 | N315 | | 3.13 | NA(+)/H(+) exchanger | COG0025P | P | Inorganic ion transport and metabolism |
| SA2233 | SA2233 | N315 | 0.05 | 0.08 | integral membrane efflux protein | | | - |
| SA2285 | SA2285 | N315 | 3.18 | 3.13 | accumulationassociated protein | | | - |
| SA2296 | SA2296 | N315 | 3.32 | | transcriptional regulator, MerR family | COG0789K | K | Transcription |
| SA2311 | SA2311 | N315 | 3.36 | | NAD(P)Hflavin oxidoreductase | COG0778C | C | Energy production and conversion |
| SA2318 | SA2318 | N315 | 3.65 | 3.02 | Lserine dehydratase | COG1760E | E | Amino acid transport and metabolism |
| SA2319 | SA2319 | N315 | 4.10 | 3.40 | betasubunit of Lserine dehydratas | COG1760E | E | Amino acid transport and metabolism |
| SA2330 | SA2330 | N315 | 4.31 | 3.61 | transcription regulator | COG0583K | K | Transcription |
| SA2342 | SA2342 | N315 | 4.81 | | Oacetyltransferase | COG0110R | R | General function prediction only |
| SA2353 | SA2353 | N315 | 3.41 | | secretory antigen precursor SsaA | COG3942R | R | General function prediction only |
| SA2354 | SA2354 | N315 | 0.25 | 0.33 | acyltransferase | COG1835I | I | Lipid transport and metabolism |
| SA2358 | SA2358 | N315 | 4.26 | 3.57 | transcriptional regulator (TetR/AcrR family) | COG1309K | K | Transcription |
| SA2367 | SA2367 | N315 | 3.00 | | conserevd HP | COG0596R | R | General function prediction only |
| SA2368 | SA2368 | N315 | 4.61 | 3.30 | cobalamin synthesis related protein CobW | COG0523R | R | General function prediction only |
| SA2369 | SA2369 | N315 | 3.26 | | ferrous iron transporter protein B | COG0370P | P | Inorganic ion transport and metabolism |
| SA2412 | SA2412 | N315 | 4.18 | | uroporphyrinIII Cmethyltransferase | COG1648H | H | Coenzyme transport and metabolism |
| SA2414 | SA2414 | N315 | 3.83 | 3.82 | glutathione peroxidase | COG0386O | O | Posttranslational modification, protein turnover, chaperones |
| SA2429 | SA2429 | N315 | 3.95 | | arginine repressor | COG1438K | K | Transcription |
| SA2434 | SA2434 | N315 | 3.09 | | fructose phosphotransferase system enzyme fruA homolog | COG1762GT,COG1299G | g | Carbohydrate transport and metabolism |
| SA2447 | SA2447 | N315 | 0.21 | 0.26 | streptococcal hemagglutinin protein | | | - |
| SA2487 | SA2487 | N315 | 3.15 | 7.30 | rarD protein | COG2962R | R | General function prediction only |

| | | | | | | | | |
|---|---|---|---|---|---|---|---|---|
| **SA2498** | SA2498 | N315 | 0.10 | 0.15 | DNAbinding protein Spo0Jlike homolog | **COG1475K** | **K** | Transcription |
| **SA0661** | *saeR* | N315 | 0.04 | 0.24 | response regulator | **COG0745TK** | **t** | Signal transduction mechanisms |
| **SA0660** | *saeS* | N315 | 0.04 | 0.12 | histidine protein kinase | **COG0642T** | **T** | Signal transduction mechanisms |
| **SA0108** | *sarS* | N315 | 4.08 | 4.20 | sarS | **COG1846K** | **K** | Transcription |
| **SAS020** | SAS020 | N315 | 4.46 | 4.61 | phosphoglycerate mutase | **COG0406G** | **G** | Carbohydrate transport and metabolism |
| **SAV0394** | SAV0394 | Mu50 | 6.29 | 4.22 | hHP | | | - |
| **SAV0913** | SAV0913 | Mu50 | 6.29 | | amidase | **COG5632M** | **M** | Cell wall/membrane/envelope biogenesis |
| **SAV1996** | SAV1996 | Mu50 | | 3.50 | hypothetical protein | | | - |
| **SA2206** | *sbi* | N315 | 0.17 | 0.33 | IgGbinding protein SBI | | | - |
| **SA0249** | *scdA* | N315 | 4.03 | | cell division and morphogenesisrelated protein | **COG2846D** | **D** | Cell cycle control, cell division, chromosome partitioning |
| **SA0995** | *sdhA* | N315 | 0.04 | 0.12 | succinate dehydrogenase flavoprotein subunit | **COG1053C** | **C** | Energy production and conversion |
| **SA0996** | *sdhB* | N315 | 0.13 | 0.16 | succinate dehydrogenase ironsulfur protein subunit | **COG0479C** | **C** | Energy production and conversion |
| **SA0994** | *sdhC* | N315 | 0.19 | 0.24 | succinate dehydrogenase cytochrome b558 | **COG2009C** | **C** | Energy production and conversion |
| **SA0521** | *sdrE* | N315 | 0.20 | 0.29 | SerAsp rich fibrinogenbinding, bone sialoproteinbinding protein | | | - |
| **SA1817** | *sec3* | N315 | 3.41 | | enterotoxin typeC3 | | | - |
| **MW0759** | *sec4* | MW2 | 3.60 | 4.76 | enterotoxin C precursor protein | | | - |
| **SA0708** | *secA* | N315 | 0.17 | | preprotein translocase subunit | **COG0653U** | **U** | Intracellular trafficking, secretion, and vesicular transport |
| **SA0493** | *secE* | N315 | | 0.19 | preprotein translocase subunit | **COG0690U** | **U** | Intracellular trafficking, secretion, and vesicular transport |
| **SA1463** | *secF* | N315 | 0.11 | 0.10 | proteinexport membrane protein SecDF | **COG0341U,COG0342U** | **U** | Intracellular trafficking, secretion, and vesicular transport |
| **SA0733** | *secG* | N315 | 0.23 | | probable proteinexport membrane protein | **COG1314U** | **U** | Intracellular trafficking, secretion, and vesicular transport |
| **SA2028** | *secY* | N315 | 0.07 | 0.28 | preprotein translocase SecY subunit | **COG0201U** | **U** | Intracellular trafficking, secretion, and vesicular transport |
| **SA1642** | *seg* | N315 | 4.90 | 3.70 | extracellular enterotoxin type G precursor | | | - |
| **SA1816** | *sel* | N315 | 3.46 | 3.09 | extracellular enterotoxin L | | | - |
| **SA1648** | *seo* | N315 | 3.94 | 6.33 | enterotoxin SeO | | | - |
| **SA1545** | *serA* | N315 | 0.25 | 0.32 | D3phosphoglycerate dehydrogenase | **COG0111HE** | **e** | Amino acid transport and metabolism |
| **SA0009** | *serS* | N315 | 0.03 | 0.02 | seryltRNA synthetase | **COG0172J** | **J** | Translation, ribosomal structure and biogenesis |
| **SA0389** | *set13* | N315 | 3.52 | 3.45 | exotoxin 13 | | | - |
| **SA0390** | *set14* | N315 | 3.77 | 3.73 | exotoxin 14 | | | - |
| **MW0385** | *set19* | MW2 | 3.97 | | exotoxin homolog [Genomic island nu Sa alpha2] | | | - |
| **MW0394** | *set26* | MW2 | | 4.95 | exotoxin homolog [Genomic island nu Sa alpha2] | | | - |
| **SA1551** | *sgtA* | N315 | 7.46 | 7.75 | probable transglycosylase | **COG0744M** | **M** | Cell wall/membrane/envelope biogenesis |
| **SA1869** | *sigB* | N315 | 0.11 | 0.15 | sigma factor B | **COG1191K** | **K** | Transcription |
| **SA0111** | *sirA* | N315 | 3.53 | | lipoprotein | **COG0614P** | **P** | Inorganic ion transport and metabolism |
| **SA1101** | *smbA* | N315 | 0.23 | 0.17 | uridylate kinase | **COG0528F** | **F** | Nucleotide transport and metabolism |
| **MW1117** | *smc* | MW2 | 0.30 | | chromosome segregation SMC protein | **COG1196D** | **D** | Cell cycle control, cell division, chromosome partitioning |
| **SA1382** | *sodA* | N315 | 3.86 | 3.14 | superoxide dismutase SodA | **COG0605P** | **P** | Inorganic ion transport and metabolism |
| **SA0107** | *spa* | N315 | 0.15 | 0.10 | Immunoglobulin G binding protein A precursor | | | - |
| **SA1631** | *splA* | N315 | 5.78 | 6.25 | serine protease SplA | **COG0265O** | **O** | Posttranslational modification, protein turnover, chaperones |
| **SA1629** | *splC* | N315 | 3.82 | 4.67 | serine protease SplC | | | - |
| **SA1628** | *splD* | N315 | 4.29 | 4.08 | serine protease SplD | **COG0265O** | **O** | Posttranslational modification, protein turnover, chaperones |
| **SA0456** | *spoVG* | N315 | 5.95 | | stage V sporulation protein G homologue | **COG2088M** | **M** | Cell wall/membrane/envelope biogenesis |
| **SA1323** | *srrA* | N315 | 0.10 | 0.23 | staphylococcal respiratory response protein SrrA | **COG0745TK** | **t** | Signal transduction mechanisms |
| **SA1322** | *srrB* | N315 | 0.10 | 0.14 | staphylococcal respiratory response protein SrrB | **COG0642T** | **T** | Signal transduction mechanisms |
| **SA2093** | *ssaA* | N315 | 0.26 | 0.24 | secretory antigen precursor SsaA homolog | **COG3942R** | **R** | General function prediction only |
| **SA0353** | *ssb* | N315 | 0.08 | 0.16 | singlestrand DNAbinding protein of phage phi PVL | **COG0629L** | **L** | Replication, recombination and repair |
| **SA0744** | *ssp* | N315 | 5.65 | 3.97 | extracellular ECM and plasma binding protein | | | - |
| **SA0901** | *sspA* | N315 | 0.14 | 0.11 | serine protease; V8 protease; glutamyl endopeptidase | | | - |
| **SA0899** | *sspC* | N315 | 3.22 | | cysteine protease | | | - |
| **SA1089** | *sucD* | N315 | 0.18 | 0.13 | succinylCoA synthetase | **COG0074C** | **C** | Energy production and conversion |
| **SA0595** | *tagB* | N315 | | 0.15 | teichoic acid biosynthesis protein B | **COG1887M** | **M** | Cell wall/membrane/envelope biogenesis |
| **SA0596** | *tagX* | N315 | 0.15 | 0.10 | teichoic acid biosynthesis protein X | | | - |
| **SA2146** | *tcaA* | N315 | 0.24 | 0.25 | TcaA protein | **COG4640S** | **S** | Function unknown |
| **SA2501** | *thdF* | N315 | 0.14 | | possible thiophene and furan oxidation protein | **COG0486R** | **R** | General function prediction only |
| **SA1165** | *thrC* | N315 | 0.08 | 0.22 | threonine synthase | **COG0498E** | **E** | Amino acid transport and metabolism |
| **SA1506** | *thrS* | N315 | 0.33 | 0.31 | threonyltRNA synthetase 1 | **COG0441J** | **J** | Translation, ribosomal structure and biogenesis |
| **SA1260** | *thyA* | N315 | 0.32 | | thymidylate synthase | | | - |
| **SA1499** | *tig* | N315 | 0.23 | | trigger factor | **COG0544O** | **O** | Posttranslational modification, protein turnover, chaperones |
| **SA1177** | *tkt* | N315 | 0.17 | 0.16 | transketolase | **COG0021G** | **G** | Carbohydrate transport and metabolism |
| **SA1038** | *tnp* | N315 | 8.62 | 4.33 | truncated transposase | **COG3666L** | **L** | Replication, recombination and repair |
| **SA2289** | *tnp* | N315 | | 5.95 | transposase | **COG3464L** | **L** | Replication, recombination and repair |
| **SA2386** | *tnpC* | N315 | 4.26 | 8.13 | transposition regulatory protein tnpC | | | - |
| **SA2051** | *topB* | N315 | 0.32 | 0.28 | DNA topoisomerase III topB | **COG0550L,COG0551L** | **L** | Replication, recombination and repair |
| **SA0729** | *tpi* | N315 | 0.02 | 0.10 | triosephosphate isomerase | **COG0149G** | **G** | Carbohydrate transport and metabolism |
| **SA0432** | *treP* | N315 | 3.60 | | PTS enzyme II, phosphoenolpyruvatedependent, trehalosespe | **COG1263G** | **G** | Carbohydrate transport and metabolism |
| **SA1204** | *trpB* | N315 | 3.42 | 3.15 | tryptophan synthase beta chain | **COG0133E** | **E** | Amino acid transport and metabolism |
| **SA1203** | *trpF* | N315 | 3.86 | 3.23 | phosphoriborylanthranilate isomerase | **COG0135E** | **E** | Amino acid transport and metabolism |
| **SA2018** | *truA* | N315 | 0.13 | 0.21 | tRNA pseudouridine synthase A | **COG0101J** | **J** | Translation, ribosomal structure and biogenesis |
| **MW1154** | *truB* | MW2 | 0.32 | | tRNA pseudouridine 5S synthase | **COG0130J** | **J** | Translation, ribosomal structure and biogenesis |
| **SA1956** | *truncatedSA* | N315 | 0.31 | 0.22 | lytic regulatory protein truncated with Tn554 | | | - |
| **SA2227** | *truncatedSA* | N315 | 3.40 | | truncatedSA | **COG1113E** | **E** | Amino acid transport and metabolism |
| **SA0719** | *trxB* | N315 | 0.04 | 0.23 | thioredoxine reductase | **COG0492O** | **O** | Posttranslational modification, protein turnover, chaperones |
| **SA1819** | *tst* | N315 | 3.80 | 4.10 | toxic shock syndrome toxin1 | | | - |
| **SA0506** | *tufA* | N315 | 0.02 | 0.10 | translational elongation factor TU | **COG0050J** | **J** | Translation, ribosomal structure and biogenesis |
| **SA1439** | *udk* | N315 | 0.11 | 0.27 | uridine kinase | **COG0572F** | **F** | Nucleotide transport and metabolism |
| **SA0214** | *uhpT* | N315 | 4.05 | 4.29 | hexose phosphate transport protein | **COG2271G** | **G** | Carbohydrate transport and metabolism |
| **SA1914** | *upp* | N315 | 0.02 | 0.06 | uracil phosphoribosyl transferase | **COG0035F** | **F** | Nucleotide transport and metabolism |
| **SA2083** | *ureB* | N315 | 4.17 | 4.10 | urease beta subunit | | | - |
| **SA2088** | *ureD* | N315 | 7.09 | 4.95 | urease accessory protein | | | - |
| **SA0714** | *uvrA* | N315 | 0.12 | 0.19 | exinuclease ABC subunit A | **COG0178L** | **L** | Replication, recombination and repair |
| **SA0713** | *uvrB* | N315 | 0.18 | 0.27 | exinuclease ABC subunit B | **COG0556L** | **L** | Replication, recombination and repair |
| **SA1488** | *valS* | N315 | 0.18 | 0.26 | valinetRNA ligase | **COG0525J** | **J** | Translation, ribosomal structure and biogenesis |
| **SA2492** | *vraD* | N315 | 4.37 | 4.10 | ABC transporter vraD | **COG1136V** | **V** | Defense mechanisms |
| **SA2493** | *vraE* | N315 | 4.13 | | ABC transporter (permease) | **COG0577V** | **V** | Defense mechanisms |
| **SA0617** | *vraG* | N315 | 0.07 | 0.13 | ABC transporter permease | | | - |
| **SA1701** | *vraS* | N315 | | 0.19 | twocomponent sensor histidine kinase | **COG4585T** | **T** | Signal transduction mechanisms |
| **SA1328** | *xerD* | N315 | 4.07 | 0.18 | sitespecific recombinase | **COG4974L** | **L** | Replication, recombination and repair |
| **SA0373** | *xprT* | N315 | 0.11 | 0.11 | xanthine phosphoribosyltransferase | **COG0503F** | **F** | Nucleotide transport and metabolism |
| **SA0041** | *xylR* | N315 | 3.75 | 4.72 | xylose repressor homologue | **COG1940KG** | **g** | Carbohydrate transport and metabolism |
| **SA1645** | *yent1* | N315 | | 3.18 | enterotoxin Yent1 | | | - |
