# Supplementary material for: A global view of Staphylococcus aureus whole genome expression upon internalization in human epithelial cells
Source: BMC Genomics. 2007 Jun 14;8:171. doi: 10.1186/1471-2164-8-171 (PMC1924023; doi:10.1186/1471-2164-8-171)
Supplement: Additional file 3 — Genes differentially expressed grouped by function. Values represent mean of fold change from 3 or 4 independent biological replicates. * Some genes are classified in two different COG categories and appear on separate lines in the table. [file 1471-2164-8-171-S3.pdf]

ORF #	Common	Organism	2h fold change	6h fold change	Protein name	GO	Class	Annotation
**SA0162**	*aldA*	N315	3.70		aldehyde dehydrogenase homologue	COG1012	C	Energy production and conversion
**SA1907**	*atpA*	N315	0.08	0.25	ATP synthase alpha chain	COG0056	C	Energy production and conversion
**SA1911**	*atpB*	N315	0.01	0.03	ATP synthase A chain		C	Energy production and conversion
**SA1904**	*atpC*	N315	0.19	0.24	FoF1ATP synthase epsilon subunit		C	Energy production and conversion
**SA1905**	*atpD*	N315	0.02	0.09	ATP synthase beta chain	COG0055	C	Energy production and conversion
**SA1910**	*atpE*	N315	0.01	0.10	ATP synthase C chain	COG0636	C	Energy production and conversion
**SA1909**	*atpF*	N315	0.01	0.12	ATP synthase B chain	COG0711	C	Energy production and conversion
**SA1906**	*atpG*	N315	0.05	0.15	ATP synthase gamma chain	COG0224	C	Energy production and conversion
**SA1908**	*atpH*	N315	0.08	0.20	ATP synthase delta chain	COG0712	C	Energy production and conversion
**SA1347**	*bfmBAB*	N315	0.23	0.28	branched-chain alphaketo acid dehydrogenase E1	COG0022	C	Energy production and conversion
**SA1346**	*bmfBB*	N315	0.31		branched-chain alphaketo acid dehydrogenase E2	COG0508	C	Energy production and conversion
**SA1184**	*citB*	N315	0.01	0.01	aconitate hydratase	COG1048	C	Energy production and conversion
**SA1517**	*citC*	N315	0.16	0.19	isocitrate dehyrogenase	COG0538	C	Energy production and conversion
**SA1518**	*citZ*	N315	0.18	0.17	citrate synthase II	COG0372	C	Energy production and conversion
**SA0820**	*glpQ*	N315	3.16		glycerophosphoryl diester phosphodiesterase	COG0584	C	Energy production and conversion
**SA1306**	*gpsA*	N315	0.05	0.08	glycerol-3-phosphate dehydrogenase	COG0240	C	Energy production and conversion
**SA0232**	*lctE*	N315	0.29	0.20	L-lactate dehydrogenase	COG0039	C	Energy production and conversion
**SA0813**	*mnhA*	N315	0.32		Na+/H+ antiporter subunit	COG2111,COG1009	C	Energy production and conversion
**SA0810**	*mnhD*	N315	0.05	0.07	Na+/H+antiporter subunit	COG0651	C	Energy production and conversion
**MW0820**	*MW0820*	MW2	0.20		NADH dehydrogenase	COG1252	C	Energy production and conversion
**MW1173**	*MW1173*	MW2		0.25	2-oxoacid ferredoxin oxidoreductase, beta subunit	COG1013	C	Energy production and conversion
**MW2317**	*MW2317*	MW2	0.10	0.09	nitrate reductase delta chain	COG2180	C	Energy production and conversion
**SA2182**	*narI*	N315	0.33	0.33	nitrate reductase gamma chain	COG2181	C	Energy production and conversion
**SA2188**	*nasD*	N315		0.33	nitrite reductase	COG1251	C	Energy production and conversion
**MW1303**	*odhA*	MW2	0.30		oxoglutarate dehydrogenase	COG0567	C	Energy production and conversion
**SA1245**	*odhA*	N315	0.02	0.31	2-oxoglutarate dehydrogenase E1	COG0567	C	Energy production and conversion
**SA1244**	*odhB*	N315	0.05	0.09	dihydrolipoamide succinyltransferase	COG0508	C	Energy production and conversion
**SA09431**	*pdhA*	N315	0.09	0.09	pyruvate dehydrogenase E1 component alpha subunit	COG1071	C	Energy production and conversion
**SA0944**	*pdhB*	N315	0.01	0.04	pyruvate dehydrogenase E1 component beta subunit	COG0022	C	Energy production and conversion
**SA0945**	*pdhC*	N315	0.01	0.03	dihydrolipoamide S Ac transferase of pyruvate dehydrogenase complex	COG0508	C	Energy production and conversion
**SA0946**	*pdhD*	N315	0.04	0.12	dihydrolipoamide dehydrogenase of pyruvate dehydrogenase E3	COG1249	C	Energy production and conversion
**SA0218**	*pflB*	N315	0.23	0.29	formate acetyltransferase	COG1882	C	Energy production and conversion
**SA0963**	*pycA*	N315	0.27	0.22	pyruvate carboxylase	COG1038	C	Energy production and conversion
**SA0912**	*qoxB*	N315	0.02	0.06	Quinol oxidase polypeptide I QoxB	COG0843	C	Energy production and conversion
**SA0911**	*qoxC*	N315	0.06	0.09	Quinol oxidase polypeptide III QoxC	COG1845	C	Energy production and conversion
**SA0036**	*SA0036*	N315		3.05	glycerophosphoryldiester phosphodiesterase homologue	COG0584	C	Energy production and conversion
**SA0220**	*SA0220*	N315	3.47	3.11	glycerophosphodiester phosphodiesterase	COG0584,COG4781	C	Energy production and conversion
**SA0231**	*SA0231*	N315	0.33		flavo-hemoprotein	COG1017,COG1018	C	Energy production and conversion
**SA0312**	*SA0312*	N315	0.33		alkanal monooxygenase alpha chain	COG2141	C	Energy production and conversion
**SA0557**	*SA0557*	N315		0.24	oxidoreductase, ion channel	COG0667	C	Energy production and conversion
**SA0640**	*SA0640*	N315	0.33	0.21	ABC transporter required for expression of cytochrome bd	COG4987COG	C	Energy production and conversion
**SA0777**	*SA0777*	N315	0.10	0.19	nitrogen fixation protein NifU	COG0822	C	Energy production and conversion
**SA0910**	*SA0910*	N315	0.05	0.13	quinol oxidase polypeptide IV QoxD	COG3125	C	Energy production and conversion
**SA0913**	*SA0913*	N315	0.05	0.10	quinol oxidase polypeptide II QoxA	COG1622	C	Energy production and conversion
**SA0969**	*SA0969*	N315		3.31	glycerophosphoryl diester phosphodiesterase	COG0584	C	Energy production and conversion
**SA1131**	*SA1131*	N315	0.07	0.25	2-oxoacid ferredoxin oxidoreductase, alpha subunit	COG1014,COG0674	C	Energy production and conversion
**SA1132**	*SA1132*	N315	0.12	0.18	2-oxoacid ferredoxin oxidoreductase, beta subunit	COG1013	C	Energy production and conversion
**SA1349**	*SA1349*	N315	0.18	0.21	dihydrolipoamide dehydrogenase	COG1249	C	Energy production and conversion
**SA1524**	*SA1524*	N315	0.24	0.32	malate dehydrogenase homolog	COG0281	C	Energy production and conversion
**SA1735**	*SA1735*	N315	0.12	0.14	manganesedependent inorganic pyrophosphatase	COG1227	C	Energy production and conversion
**SA2311**	*SA2311*	N315	3.36		NAD(P)Hflavin oxidoreductase	COG0778	C	Energy production and conversion
**SA0995**	*sdhA*	N315	0.04	0.12	succinate dehydrogenase flavoprotein subunit	COG1053	C	Energy production and conversion
**SA0996**	*sdhB*	N315	0.13	0.16	succinate dehydrogenase ironsulfur protein subunit	COG0479	C	Energy production and conversion
**SA0994**	*sdhC*	N315	0.19	0.24	succinate dehydrogenase cytochrome b558	COG2009	C	Energy production and conversion
**SA1089**	*sucD*	N315	0.18	0.13	succinylCoA synthetase	COG0074	C	Energy production and conversion
**SA0846**	*SA0846*	N315	0.01	0.01	oligopeptide transport system permease protein OppC	COG1173	E	Amino acid transport and metabolism
**SA0848**	*oppF*	N315	0.01	0.02	oligopeptide transport system ATPbinding protein OppF homologue	COG4608	E	Amino acid transport and metabolism
**SA0849**	*SA0849*	N315	0.02	0.02	peptide binding protein OppA	COG4166	E	Amino acid transport and metabolism
**SA1424**	*aroE*	N315	0.03	0.04	shikimate dehydrogenease	COG0169	E	Amino acid transport and metabolism
**SA1045**	*pyrAA*	N315	0.03	0.20	carbamoylphosphate synthase small chain	COG0505	E	Amino acid transport and metabolism
**SA1915**	*glyA*	N315	0.03	0.06	serine hydroxymethyl transferase	COG0112	E	Amino acid transport and metabolism
**SA1572**	*SA1572*	N315	0.03	0.03	XaaHis dipeptidase homolog	COG0624	E	Amino acid transport and metabolism
**SA0242**	*SA0242*	N315	0.04	0.04	xylitol dehydrogenase	COG1063	E	Amino acid transport and metabolism
**SA0847**	*oppD*	N315	0.05	0.05	oligopeptide transport system ATPbinding protein OppD homologue	COG0444	E	Amino acid transport and metabolism
**SA1365**	*SA1365*	N315	0.06	0.08	glycine dehydrogenase (decarboxylating) subunit 2 homolog	COG1003	E	Amino acid transport and metabolism
**MW1267**	*opp2F*	MW2	0.06	0.09	oligopeptide transporter putative ATPase domain	COG4608	E	Amino acid transport and metabolism
**SA1232**	*lysA*	N315	0.07	0.12	diaminopimelate decarboxylase	COG0019	E	Amino acid transport and metabolism
**SA1227**	*dapA*	N315	0.08	0.14	dihydrodipicolinate synthase	COG0329	E	Amino acid transport and metabolism
**SA1297**	*aroA*	N315	0.08	0.17	3 phosphoshikimate 1-carboxyvinyl-transferase	COG0128	E	Amino acid transport and metabolism
**SA1165**	*thrC*	N315	0.08	0.22	threonine synthase	COG0498	E	Amino acid transport and metabolism
**SA0246**	*SA0246*	N315	0.08	0.18	hypotheticl protein, similar to D-xylulose reductase	COG1063	E	Amino acid transport and metabolism
**SA1859**	*ilvB*	N315	0.08	0.08	acetolactate synthase large subunit	COG0028	E	Amino acid transport and metabolism
**SA1046**	*pyrAB*	N315	0.09	0.21	carbamoylphosphate synthase large chain	COG0458	E	Amino acid transport and metabolism
**SA1225**	*lysC*	N315	0.09	0.11	aspartokinase II	COG0527	E	Amino acid transport and metabolism
**SA1367**	*SA1367*	N315	0.09	0.12	aminomethyltransferase	COG0404	E	Amino acid transport and metabolism
**SA1366**	*SA1366*	N315	0.10	0.12	glycine dehydrogenase subunit 1	COG0403	E	Amino acid transport and metabolism
**SA0670**	*SA0670*	N315	0.10	0.29	paraaminobenzoate synthase component I	COG0115	E	Amino acid transport and metabolism
**SA2125**	*SA2125*	N315	0.13	0.14	formiminoglutamase	COG0010	E	Amino acid transport and metabolism
**SA1226**	*asd*	N315	0.13	0.23	aspartate semialdehyde dehydrogenase	COG0136	E	Amino acid transport and metabolism
**SA0845**	*oppB*	N315	0.16	0.14	oligopeptide transport system permease protein	COG0601	E	Amino acid transport and metabolism
**SA1519**	*aapA*	N315	0.17	0.17	D serine/Dalanine/glycine transporter	COG1113	E	Amino acid transport and metabolism
**SA1695**	*ampS*	N315	0.17	0.18	aminopeptidase ampS	COG2309	E	Amino acid transport and metabolism
**SA1190**	*alsT*	N315	0.18		amino acid carrier protein	COG1115	E	Amino acid transport and metabolism
**SA0010**	*SA0010*	N315	0.19	0.17	amino acid permease	COG1296	E	Amino acid transport and metabolism
**SA0471**	*cysK*	N315	0.19	0.12	cysteine synthase (oacetylserine sulfhydrylase) homologue	COG0031	E	Amino acid transport and metabolism
**SA0953**	*potD*	N315	0.20	0.29	spermidine/putrescinebinding protein precursor homolog	COG0687	E	Amino acid transport and metabolism
**SA2201**	*SA2201*	N315	0.20	0.24	ABC transporter, permease protein	COG0765	E	Amino acid transport and metabolism
**SA0951**	*potB*	N315	0.20	0.22	potB	COG1176	E	Amino acid transport and metabolism
**SA0678**	*SA0678*	N315	0.22	0.25	choline transporter	COG1174,COG1732	E	Amino acid transport and metabolism
**MW0539**	*MW0539*	MW2	0.23	0.26	cationic amino acid transporter	COG0531	E	Amino acid transport and metabolism
**SA2405**	*betA*	N315	0.24	0.30	choline dehydrogenase	COG2303	E	Amino acid transport and metabolism
**SA1538**	*SA1538*	N315	0.24	0.23	ironsulfur cofactor synthesis protein nifZ	COG1104	E	Amino acid transport and metabolism
**SA1169**	*SA1169*	N315	0.24	0.21	gammaaminobutyrate permease	COG0833	E	Amino acid transport and metabolism
**SA0990**	*SA0990*	N315	0.25		DNAdependent DNA polymerase beta chain	COG1796L,COG138	E	Amino acid transport and metabolism
**SA0682**	*SA0682*	N315	0.25	0.14	ditripepride ABC transporter	COG3104	E	Amino acid transport and metabolism
**SA0679**	*SA0679*	N315	0.25	0.17	histidinol-phosphate aminotransferase	COG0079	E	Amino acid transport and metabolism
**SA1545**	*serA*	N315	0.25	0.32	D3phosphoglycerate dehydrogenase	COG0111	E	Amino acid transport and metabolism
**SA1571**	*SA1571*	N315	0.26	0.24	Dalanine aminotransferase	COG0115	E	Amino acid transport and metabolism

ORF #	Common	Organism	2h fold change	6h fold change	Protein name	GO	Class	Annotation
**SA0776**	*SA0776*	N315	0.26		aminotransferase NifS homologue	COG0520	**E**	Amino acid transport and metabolism
**SA1298**	*aroB*	N315	0.27		3 dehydroquinate synthase	COG0337	**E**	Amino acid transport and metabolism
**SA0818**	*rocD*	N315	0.27	0.16	ornithine aminotransferase	COG4992	**E**	Amino acid transport and metabolism
**SA0950**	*potA*	N315	0.27		spermidine/putrescine ABC transporter, ATPbinding protein homolog	COG3842	**E**	Amino acid transport and metabolism
**SA0859**	*SA0859*	N315	0.28	0.22	thimet oligopeptidase homologue	COG1164	**E**	Amino acid transport and metabolism
**SA1164**	*dhoM*	N315	0.28		homoserine dehydrogenase	COG0460	**E**	Amino acid transport and metabolism
**SA0482**	*SA0482*	N315	0.29		creatine kinase	COG3869	**E**	Amino acid transport and metabolism
**SA0313**	*SA0313*	N315	0.29	0.20	glycine cleavage system H protein	COG0509	**E**	Amino acid transport and metabolism
**SA0822**	*argG*	N315	0.30		argininosuccinate synthase	COG0137	**E**	Amino acid transport and metabolism
**MW1284**	*dapB*	MW2	0.31	0.29	dihydrodipicolinate reductase	COG0289	**E**	Amino acid transport and metabolism
**SA0677**	*SA0677*	N315	0.31		choline transport ATPbinding protein	COG1125	**E**	Amino acid transport and metabolism
**SA2200**	*SA2200*	N315	0.31		ABC transporter, ATP binding subunit	COG1126	**E**	Amino acid transport and metabolism
**SA0487**	*cysE*	N315	0.32		Serine acetyltransferase homologue	COG1045	**E**	Amino acid transport and metabolism
**SA0458**	*prs*	N315	0.32	0.26	ribosephosphate pyrophosphokinase	COG0462	**E**	Amino acid transport and metabolism
**MW1625**	*lysP*	MW2	0.32		lysine-specific permease	COG0833	**E**	Amino acid transport and metabolism
**SA2081**	*SA2081*	N315	3.02	4.18	urea transporter	COG4413	**E**	Amino acid transport and metabolism
**SA0112**	*SA0112*	N315	3.06	3.47	cysteine synthase	COG0031	**E**	Amino acid transport and metabolism
**SA1531**	*ald*	N315	3.07		alanine dehydrogenase	COG0686	**E**	Amino acid transport and metabolism
**SA0303**	*SA0303*	N315	3.08		sodiumcoupled permease	COG0591	**E**	Amino acid transport and metabolism
**SA2293**	*gntP*	N315	3.13		gluconate permease	COG2610	**E**	Amino acid transport and metabolism
**SA1749**	*SA1749*	N315	3.22		aspartate transaminase protein	COG0436	**E**	Amino acid transport and metabolism
**SA0304**	*nanA*	N315	3.30		N acetylneuraminate lyase subunit	COG0329	**E**	Amino acid transport and metabolism
**MW1680**	*MW1680*	MW2	3.37	4.67	chorismate mutase homolog	COG2876,COG1605	**E**	Amino acid transport and metabolism
**SA0418**	*cysM*	N315	3.39	3.69	cysteine synthase homologue	COG0031	**E**	Amino acid transport and metabolism
**SA2227**	*truncatedSA*	N315	3.40		truncatedSA	COG1113	**E**	Amino acid transport and metabolism
**SA1204**	*trpB*	N315	3.42	3.15	tryptophan synthase beta chain	COG0133	**E**	Amino acid transport and metabolism
**SA0698**	*pepT*	N315	3.46		aminotripeptidase	COG2195	**E**	Amino acid transport and metabolism
**SA1814**	*SA1814*	N315	3.57		succinyldiaminopimelate desuccinylase	COG0624	**E**	Amino acid transport and metabolism
**SA2318**	*SA2318*	N315	3.65	3.02	Lserine dehydratase	COG1760	**E**	Amino acid transport and metabolism
**SA1270**	*SA1270*	N315	3.70	3.64	amino acid pearmease	COG0531	**E**	Amino acid transport and metabolism
**SA0201**	*rlp*	N315	3.83	3.52	RGDcontaining lipoprotein	COG0747	**E**	Amino acid transport and metabolism
**SA1203**	*trpF*	N315	3.86	3.23	phosphoriborylanthranilate isomerase	COG0135	**E**	Amino acid transport and metabolism
**SA2430**	*aur*	N315	3.91		zinc metallo-proteinase aureolysin	COG3227	**E**	Amino acid transport and metabolism
**SA2319**	*SA2319*	N315	4.10	3.40	betasubunit of Lserine dehydratas	COG1760	**E**	Amino acid transport and metabolism
**SA0476**	*SA0476*	N315	4.69	3.13	transcription regulator GntR family	COG1167	**E**	Amino acid transport and metabolism
**SA0851**	*SA0851*	N315	5.26	4.72	oligopeptide ABC transporter ATPbinding protein homologue	COG0444	**E**	Amino acid transport and metabolism
**SA0113**	*SA0113*	N315	24.10	27.78	ornithine cyclodeaminase	COG2423	**E**	Amino acid transport and metabolism
**SA0119**	*SA0119*	N315		3.13	diaminopimelate decarboxylase	COG0019	**E**	Amino acid transport and metabolism
**SA0180**	*SA0180*	N315		0.30	branched-chain amino acid transport system carrier protein	COG1114	**E**	Amino acid transport and metabolism
**SA0819**	*gudB*	N315		0.33	NADspecific glutamate dehydrogenase	COG0334	**E**	Amino acid transport and metabolism
**SA1216**	*SA1216*	N315		0.24	oligoendopeptidase	COG1164	**E**	Amino acid transport and metabolism
**SA1730**	*SA1730*	N315		0.28	nitricoxide synthase homolog	COG4362	**E**	Amino acid transport and metabolism
**SA2124**	*fosB*	N315		3.45	fosfomycin resistance protein fofB	COG0346	**E**	Amino acid transport and metabolism
**SA2234**	*opuCD*	N315		3.10	probable glycine betaine/carnitine/choline ABC transporter opuCD	COG1174	**E**	Amino acid transport and metabolism
**SA0917**	*purK*	N315	0.00	0.02	P-ribosylaminoimidazole carboxylase carbon dioxidefixation chain	COG0026	**F**	Nucleotide transport and metabolism
**SA0920**	*purQ*	N315	0.01	0.02	phosphoribosylformylglycinamidine synthase I PurQ	COG0047	**F**	Nucleotide transport and metabolism
**SA2027**	*adk*	N315	0.01	0.15	adenylate kinase	COG0563	**F**	Nucleotide transport and metabolism
**SA0926**	*purD*	N315	0.01	0.01	phosphoribosylamineglycine ligase PurD	COG0151	**F**	Nucleotide transport and metabolism
**SA0921**	*purL*	N315	0.01	0.02	phosphoribosylformylglycinamidine synthetase	COG0046	**F**	Nucleotide transport and metabolism
**SA1048**	*pyrE*	N315	0.01	0.03	orotate phosphoribosyltransferase	COG0461	**F**	Nucleotide transport and metabolism
**SA0923**	*purM*	N315	0.02	0.03	phosphoribosylformylglycinamidine cycloligase PurM	COG0150	**F**	Nucleotide transport and metabolism
**SA1914**	*upp*	N315	0.02	0.06	uracil phosphoribosyl transferase	COG0035	**F**	Nucleotide transport and metabolism
**SA1044**	*pyrC*	N315	0.03	0.10	dihydroorotase	COG0044	**F**	Nucleotide transport and metabolism
**SA0374**	*pbuX*	N315	0.03	0.05	xanthine permease	COG2233	**F**	Nucleotide transport and metabolism
**SA1043**	*pyrB*	N315	0.03	0.08	aspartate transcarbamoylase chain A	COG0540	**F**	Nucleotide transport and metabolism
**SA0922**	*purF*	N315	0.04	0.06	phosphoribosylpyrophosphate amidotransferase PurF	COG0034	**F**	Nucleotide transport and metabolism
**SA0454**	*purR*	N315	0.05	0.11	pur operon repressor homologue	COG0503	**F**	Nucleotide transport and metabolism
**SA1047**	*pyrF*	N315	0.05	0.07	orotidine5phosphate decarboxylase	COG0284	**F**	Nucleotide transport and metabolism
**SA1042**	*pyrP*	N315	0.08	0.24	uracil permease	COG2233	**F**	Nucleotide transport and metabolism
**SA0468**	*SA0468*	N315	0.09	0.17	hypoxanthineguanine phosphoribosyltransferase homologue	COG0634	**F**	Nucleotide transport and metabolism
**SA0916**	*SA0916*	N315	0.10	0.10	phosphoribosylaminoimidazole carboxylase PurE	COG0041	**F**	Nucleotide transport and metabolism
**SA1929**	*ctrA*	N315	0.10	0.11	CTP synthase	COG0504	**F**	Nucleotide transport and metabolism
**SA1439**	*udk*	N315	0.11	0.27	uridine kinase	COG0572	**F**	Nucleotide transport and metabolism
**SA0376**	*guaA*	N315	0.11	0.12	GMP synthase	COG0518,COG0519	**F**	Nucleotide transport and metabolism
**SA0373**	*xprT*	N315	0.11	0.11	xanthine phosphoribosyltransferase	COG0503	**F**	Nucleotide transport and metabolism
**SA1553**	*fhs*	N315	0.14	0.19	formyltetrahydrofolate synthetase	COG2759	**F**	Nucleotide transport and metabolism
**SA1397**	*cdd*	N315	0.15	0.19	cytidine deaminase	COG0295	**F**	Nucleotide transport and metabolism
**SA1939**	*SA1939*	N315	0.15	0.25	deoxyribosephosphate aldolase	COG0274	**F**	Nucleotide transport and metabolism
**SA1172**	*SA1172*	N315	0.16	0.27	GMP reductase	COG0516	**F**	Nucleotide transport and metabolism
**SA1724**	*purB*	N315	0.21	0.21	adenylosuccinate lyase	COG0015	**F**	Nucleotide transport and metabolism
**SA1101**	*smbA*	N315	0.23	0.17	uridylate kinase	COG0528	**F**	Nucleotide transport and metabolism
**SA1938**	*pdp*	N315	0.24	0.25	pyrimidine nucleoside phosphorylase	COG0213	**F**	Nucleotide transport and metabolism
**SA0686**	*nrdE*	N315	0.28	0.29	ribonuceloside diphosphate reductase major subunit	COG0209	**F**	Nucleotide transport and metabolism
**SA0234**	*SA0234*	N315	3.76	3.55	inosineuridine preferring nucleoside hydrolase	COG1957	**F**	Nucleotide transport and metabolism
**SA0881**	*SA0881*	N315	3.76	3.57	nucleotidase	COG0737	**F**	Nucleotide transport and metabolism
**MW1034**	*MW1034*	MW2		0.13	hypothetical protein	COG0127	**F**	Nucleotide transport and metabolism
**SA0131**	*pnp*	N315		3.05	purine nucleoside phosphorylase	COG0813	**F**	Nucleotide transport and metabolism
**SA0729**	*tpi*	N315	0.02	0.10	triosephosphate isomerase	COG0149	**G**	Carbohydrate transport and metabolism
**SA0823**	*pgi*	N315	0.02	0.04	glucose6phosphate isomerase A	COG0166	**G**	Carbohydrate transport and metabolism
**SA0731**	*eno*	N315	0.04	0.13	enolase	COG0148	**G**	Carbohydrate transport and metabolism
**SA0905**	*atl*	N315	0.04	0.20	autolysin	COG4193,COG5632	**G**	Carbohydrate transport and metabolism
**SA2129**	*SA2129*	N315	0.06	0.04	aldose 1epimerase	COG2017	**G**	Carbohydrate transport and metabolism
**SA0935**	*ptsI*	N315	0.07	0.07	phosphoenolpyruvateprotein phosphatase	COG1080	**G**	Carbohydrate transport and metabolism
**SA0528**	*SA0528*	N315	0.07	0.06	hexulose-6-phosphate synthase	COG0269	**G**	Carbohydrate transport and metabolism
**SA0730**	*pgm*	N315	0.08	0.22	2, 3diphosphoglycerateindependentphosphoglycer ate mutase	COG0696	**G**	Carbohydrate transport and metabolism
**SA0934**	*ptsH*	N315	0.09	0.13	phophocarrier protein HPR	COG1925	**G**	Carbohydrate transport and metabolism
**SA1342**	*gnd*	N315	0.09	0.09	phosphogluconate dehydrogenase	COG0362	**G**	Carbohydrate transport and metabolism
**SA0727**	*gap*	N315	0.10	0.27	glyceraldehyde3phosphate dehydrogenase	COG0057	**G**	Carbohydrate transport and metabolism
**SA0790**	*SA0790*	N315	0.12	0.17	Nacetylglucosamine catabolism homologue	COG0647	**G**	Carbohydrate transport and metabolism
**SA2326**	*ptsG*	N315	0.13	0.17	PTS system, glucosespecific IIABC component	COG1263	**G**	Carbohydrate transport and metabolism
**SA1927**	*fbaA*	N315	0.14	0.15	fructosebisphosphate aldolase	COG0191	**G**	Carbohydrate transport and metabolism
**SA0183**	*glcA*	N315	0.15	0.14	PTS enzyme II (EC 2.7.1.69), glucosespecific, factor IIA homologue	COG1263	**G**	Carbohydrate transport and metabolism
**SA1177**	*tkt*	N315	0.17	0.16	transketolase	COG0021	**G**	Carbohydrate transport and metabolism
**SA1688**	*SA1688*	N315	0.17	0.33	teichoic acid translocation ATPbinding protein tagH	COG1134	**G**	Carbohydrate transport and metabolism
**SA0157**	*capN*	N315	0.18	0.32	capsular polysaccharide synthesis enzyme Cap5N	COG0451	**G**	Carbohydrate transport and metabolism
**SA1377**	*glcK*	N315	0.19	0.16	glucokinase	COG1940	**G**	Carbohydrate transport and metabolism
**SA1547**	*ptaA*	N315	0.19	0.23	PTS system, Nacetylglucosaminespecific IIABC component	COG1263	**G**	Carbohydrate transport and metabolism
**SA1521**	*pfk*	N315	0.23	0.32	6phosphofructokinase	COG0205	**G**	Carbohydrate transport and metabolism
**SA0149**	*capF*	N315	0.23	0.25	capsular polysaccharide synthesis enzyme Cap5F	COG1898,COG0451	**G**	Carbohydrate transport and metabolism

ORF #	Common	Organism	2h fold change	6h fold change	Protein name	GO	Class	Annotation
**SA0605**	*SA0605*	N315	0.24	0.27	dihydroxyacetone kinase	COG2376	**G**	Carbohydrate transport and metabolism
**SA0259**	*rbsD*	N315	0.27	0.22	ribose permease	COG1869	**G**	Carbohydrate transport and metabolism
**SA1065**	*cfxE*	N315	0.28	0.31	ribulose5phosphate 3epimerase homolog	COG0036	**G**	Carbohydrate transport and metabolism
**SA1965**	*glmM(femD)*	N315	0.32	0.28	phospho glucosamine mutase	COG1109	**G**	Carbohydrate transport and metabolism
**SA0134**	*drm*	N315	0.32		phosphopentomutase	COG1015	**G**	Carbohydrate transport and metabolism
**SA2434**	*SA2434*	N315	3.09		fructose phosphotransferase system enzyme fruA homolog	COG1762,COG1299	**G**	Carbohydrate transport and metabolism
**SA2127**	*SA2127*	N315	3.13	4.10	ribose 5phosphate isomerase (rpi)	COG0120	**G**	Carbohydrate transport and metabolism
**SA1995**	*lacC*	N315	3.22	3.14	tagatose-6-phosphate kinase	COG1105	**G**	Carbohydrate transport and metabolism
**SA2480**	*drp35*	N315	3.25	3.47	Drp35	COG3386	**G**	Carbohydrate transport and metabolism
**SA1994**	*lacD*	N315	3.30	3.82	tagatose 1,6-diphosphate aldolase	COG3684	**G**	Carbohydrate transport and metabolism
**SA1997**	*lacA*	N315	3.37		galactose-6-phosphate isomerase LacA subunit	COG0698	**G**	Carbohydrate transport and metabolism
**SA0655**	*fruA*	N315	3.47	3.65	fructose specific permease	COG1762,COG1299	**G**	Carbohydrate transport and metabolism
**SA2294**	*gntK*	N315	3.51		gluconokinase	COG1070	**G**	Carbohydrate transport and metabolism
**SA0299**	*SA0299*	N315	3.53	3.60	carbohydrate kinase, PfkB family	COG0524	**G**	Carbohydrate transport and metabolism
**SA0432**	*treP*	N315	3.60		PTS enzyme II, phosphoenolpyruvatedependent, trehalosespecific	COG1263	**G**	Carbohydrate transport and metabolism
**SA0041**	*xylR*	N315	3.75	4.72	xylose repressor homologue	COG1940	**G**	Carbohydrate transport and metabolism
**SA2462**	*icaC*	N315	3.91	5.18	intercellular adhesion protein C	COG3936	**G**	Carbohydrate transport and metabolism
**SA0214**	*uhpT*	N315	4.05	4.29	hexose phosphate transport protein	COG2271	**G**	Carbohydrate transport and metabolism
**SA0361**	*SA0361*	N315	4.22	5.26	truncated phosphoglycerate mutase; Gpm3p	COG0406	**G**	Carbohydrate transport and metabolism
**SA0207**	*SA0207*	N315	4.42		maltose/maltodextrinbinding protein	COG2182	**G**	Carbohydrate transport and metabolism
**SAS020**	*SAS020*	N315	4.46	4.61	phosphoglycerate mutase	COG0406	**G**	Carbohydrate transport and metabolism
**SA0325**	*glpT*	N315	4.74	4.10	glycerol3phosphate transporter	COG2271	**G**	Carbohydrate transport and metabolism
**SA1996**	*lacB*	N315	62.50	65.36	galactose6phosphate isomerase LacB subunit	COG0698	**G**	Carbohydrate transport and metabolism
**MW0113**	*drm*	MW2		0.18	phosphopentomutase	COG1015	**G**	Carbohydrate transport and metabolism
**MW0381**	*MW0381*	MW2		4.63	hypothetical protein	COG0702	**G**	Carbohydrate transport and metabolism
**MW0778**	*MW0778*	MW2		3.75	phosphoglycerate mutase	COG0406	**G**	Carbohydrate transport and metabolism
**SA1729**	*SA1729*	N315	0.01	0.04	nicotinate phosphoribosyltransferase homolog	COG1488	**H**	Coenzyme transport and metabolism
**SA1537**	*SA1537*	N315	0.01	0.12	thiamine biosynthesis protein ThiI	COG0301	**H**	Coenzyme transport and metabolism
**SA1491**	*hemL*	N315	0.03	0.03	glutamate1semialdehyde 2,1aminomutase	COG0001	**H**	Coenzyme transport and metabolism
**SA1259**	*dfrA*	N315	0.03	0.20	dihydrofolate reductase	COG0262	**H**	Coenzyme transport and metabolism
**SA2212**	*SA2212*	N315	0.05	0.28	8-amino 7-oxononanoate synthase	COG0156	**H**	Coenzyme transport and metabolism
**SA0915**	*folD*	N315	0.05	0.08	FolD bifunctional protein	COG0190	**H**	Coenzyme transport and metabolism
**SA2067**	*mobB*	N315	0.06	0.14	probable molybdopteringuanine dinucleotide biosynthesis mobB	COG1763	**H**	Coenzyme transport and metabolism
**SA2213**	*bioB*	N315	0.08	0.14	biotin synthase	COG0502	**H**	Coenzyme transport and metabolism
**SA1728**	*nadE*	N315	0.08	0.08	NAD synthetase, prefers NH3 over glutamine	COG0171	**H**	Coenzyme transport and metabolism
**MW1853**	*nadE*	MW2	0.12	0.08	NAD synthetase	COG0171	**H**	Coenzyme transport and metabolism
**SA1487**	*folC*	N315	0.12	0.33	folylpolyglutamate synthase	COG0285	**H**	Coenzyme transport and metabolism
**SA1652**	*hemE*	N315	0.14		uroporphyrinogen decarboxylase	COG0407	**H**	Coenzyme transport and metabolism
**SA2392**	*panB*	N315	0.14	0.21	3methyl2oxobutanoate hydroxymethyltransferase	COG0413	**H**	Coenzyme transport and metabolism
**SA1650**	*hemY*	N315	0.15	0.23	protoporphyrinogen oxidase	COG1232	**H**	Coenzyme transport and metabolism
**SA1496**	*hemA*	N315	0.15	0.28	glutamyltRNA reductase	COG0373	**H**	Coenzyme transport and metabolism
**SA2064**	*mobA*	N315	0.15	0.24	molybdopteringuanine dinucleotide biosynthesis mobA	COG0746	**H**	Coenzyme transport and metabolism
**SA2071**	*moeB*	N315	0.16	0.24	molybdopterin biosynthesis protein moeB	COG0476	**H**	Coenzyme transport and metabolism
**SA2068**	*moeA*	N315	0.16	0.28	molybdopterin biosynthesis protein moeA	COG0303	**H**	Coenzyme transport and metabolism
**SA0896**	*menD*	N315	0.23	0.26	menaquinone biosynthesis protein	COG1165	**H**	Coenzyme transport and metabolism
**SA1493**	*hemD*	N315	0.24		uroporphyrinogen III synthase	COG1587	**H**	Coenzyme transport and metabolism
**SA0791**	*SA0791*	N315	0.25	0.20	glycerate dehydrogenase	COG1052	**H**	Coenzyme transport and metabolism
**SA0602**	*fhuA*	N315	0.28		ferrichrome transport ATPbinding protein	COG1120	**H**	Coenzyme transport and metabolism
**SA2065**	*moaD*	N315	0.31	0.28	probable molybdopterin synthase small subunit	COG1977	**H**	Coenzyme transport and metabolism
**SA1302**	*gerCC*	N315	0.32	0.22	heptaprenyl diphosphate syntase component II	COG0142	**H**	Coenzyme transport and metabolism
**SA1289**	*SA1289*	N315	0.32	0.33	bifunctional biotin ligase/biotin operon repressor	COG0340,COG1654	**H**	Coenzyme transport and metabolism
**SA2063**	*moaA*	N315	0.32		molybdenum cofactor biosynthesis protein A	COG2896	**H**	Coenzyme transport and metabolism
**SA0316**	*SA0316*	N315	0.33		lipoateprotein ligase	COG0095	**H**	Coenzyme transport and metabolism
**SA0895**	*SA0895*	N315	3.40	4.07	menaquinonespecific isochorismate synthase	COG1169Q	**H**	Coenzyme transport and metabolism
**SA1587**	*ribA*	N315	3.44		riboflavin biosynthesis protein	COG0807	**H**	Coenzyme transport and metabolism
**SA0472**	*folP*	N315	3.52		dihydropteroate synthase chain A synthetase	COG0294	**H**	Coenzyme transport and metabolism
**SA2412**	*SA2412*	N315	4.18		uroporphyrinIII Cmethyltransferase	COG1648	**H**	Coenzyme transport and metabolism
**MW0812**	*MW0812*	MW2		0.26	glycerate dehydrogenase	COG1052	**H**	Coenzyme transport and metabolism
**SA2025**	*rpsM*	N315	0.01	0.12	30S Protein synthesis S13	COG0099	**J**	Translation, ribosomal structure and biogenesis
**SAS078**	*rpmJ*	N315	0.01	0.17	50S Protein synthesis L36	COG0257	**J**	Translation, ribosomal structure and biogenesis
**SA2026**	*infA*	N315	0.01	0.16	translation initiation factor IF1	COG0361	**J**	Translation, ribosomal structure and biogenesis
**SA1112**	*infB*	N315	0.02	0.14	translation initiation factor IF2	COG0532	**J**	Translation, ribosomal structure and biogenesis
**SA2039**	*rpmC*	N315	0.03	0.26	50S Protein synthesis L29		**J**	Translation, ribosomal structure and biogenesis
**SA2035**	*rplE*	N315	0.03	0.26	50S Protein synthesis L5	COG0094	**J**	Translation, ribosomal structure and biogenesis
**SA2038**	*rpsQ*	N315	0.03	0.19	30S Protein synthesis S17	COG0186	**J**	Translation, ribosomal structure and biogenesis
**SA2041**	*rpsC*	N315	0.03	0.21	30S Protein synthesis S3	COG0092	**J**	Translation, ribosomal structure and biogenesis
**SA2034**	*rpsH*	N315	0.03	0.25	30S Protein synthesis S8		**J**	Translation, ribosomal structure and biogenesis
**SA1094**	*gid*	N315	0.04	0.07	glucoseinhibited division protein gid	COG1206	**J**	Translation, ribosomal structure and biogenesis
**SA2029**	*rplO*	N315	0.04	0.32	50S Protein synthesis L15	COG0200	**J**	Translation, ribosomal structure and biogenesis
**SA2036**	*rplX*	N315	0.04	0.22	50S Protein synthesis L24	COG0198	**J**	Translation, ribosomal structure and biogenesis
**SA0504**	*rpsG*	N315	0.04	0.23	30S Protein synthesis S7	COG0049	**J**	Translation, ribosomal structure and biogenesis
**SA2033**	*rplF*	N315	0.04		50S Protein synthesis L6	COG0097	**J**	Translation, ribosomal structure and biogenesis
**SA2030**	*rpmD*	N315	0.05		50S Protein synthesis L30	COG1841	**J**	Translation, ribosomal structure and biogenesis
**SA1579**	*leuS*	N315	0.05	0.15	leucyltRNA synthetase	COG0495	**J**	Translation, ribosomal structure and biogenesis
**SA0475**	*lysS*	N315	0.05	0.13	lysyl-tRNA synthetase	COG1190	**J**	Translation, ribosomal structure and biogenesis
**SAS079**	*rpsN*	N315	0.06	0.20	30S Protein synthesis S14	COG0199	**J**	Translation, ribosomal structure and biogenesis
**SA2032**	*rplR*	N315	0.06	0.33	50S Protein synthesis L18	COG0256	**J**	Translation, ribosomal structure and biogenesis
**SA2040**	*rplP*	N315	0.06	0.21	50S Protein synthesis L16	COG0197	**J**	Translation, ribosomal structure and biogenesis
**SA0503**	*rpsL*	N315	0.06	0.20	30S Protein synthesis S12		**J**	Translation, ribosomal structure and biogenesis
**SA0505**	*fus*	N315	0.07	0.25	translational elongation factor G	COG0480	**J**	Translation, ribosomal structure and biogenesis
**SA2042**	*rplV*	N315	0.07	0.23	50S Protein synthesis L22		**J**	Translation, ribosomal structure and biogenesis
**SA2037**	*rplN*	N315	0.08	0.23	50S Protein synthesis L14		**J**	Translation, ribosomal structure and biogenesis
**SA2043**	*rpsS*	N315	0.08		30S Protein synthesis S19	COG0185	**J**	Translation, ribosomal structure and biogenesis
**SA0488**	*cysS*	N315	0.09	0.13	cysteinyltRNA synthetase	COG0215	**J**	Translation, ribosomal structure and biogenesis
**SA1456**	*aspS*	N315	0.10	0.06	aspartyltRNA synthetase	COG0173	**J**	Translation, ribosomal structure and biogenesis
**SA2047**	*rplC*	N315	0.10	0.20	50S Protein synthesis L3		**J**	Translation, ribosomal structure and biogenesis
**SA0877**	*prfC*	N315	0.11	0.27	peptide chain release factor 3	COG4108	**J**	Translation, ribosomal structure and biogenesis
**SA1099**	*rpsB*	N315	0.11	0.26	30S Protein synthesis S2		**J**	Translation, ribosomal structure and biogenesis
**SAS042**	*rpmG*	N315	0.11	0.30	50S Protein synthesis L33	COG0267	**J**	Translation, ribosomal structure and biogenesis
**SA2022**	*rplQ*	N315	0.12		50S Protein synthesis L17	COG0203	**J**	Translation, ribosomal structure and biogenesis
**SA0985**	*pheS*	N315	0.13	0.19	PhetRNA synthetase alpha chain	COG0016	**J**	Translation, ribosomal structure and biogenesis
**SA2044**	*rplB*	N315	0.13		50S Protein synthesis L2	COG0090	**J**	Translation, ribosomal structure and biogenesis
**SA2017**	*rplM*	N315	0.14	0.22	50S Protein synthesis L13		**J**	Translation, ribosomal structure and biogenesis
**SA2031**	*rpsE*	N315	0.14	0.30	30S Protein synthesis S5	COG0098	**J**	Translation, ribosomal structure and biogenesis
**SA1117**	*pnpA*	N315	0.15	0.18	polyribonucleotide nucleotidyltransferase	COG1185	**J**	Translation, ribosomal structure and biogenesis
**SA2045**	*rplW*	N315	0.15	0.23	50S Protein synthesis L23	COG0089	**J**	Translation, ribosomal structure and biogenesis
**SA1473**	*rplU*	N315	0.16	0.24	50S Protein synthesis L21		**J**	Translation, ribosomal structure and biogenesis
**SA0498**	*rplL*	N315	0.16		50S Protein synthesis L7/L12	COG0222	**J**	Translation, ribosomal structure and biogenesis
**SA1324**	*rluB*	N315	0.16	0.31	ribosomal large subunit pseudouridine synthase B	COG1187	**J**	Translation, ribosomal structure and biogenesis

ORF #	Common	Organism	2h fold change	6h fold change	Protein name	GO	Class	Annotation
**SA0014**	*rplI*	N315	0.18	0.16	50S Protein synthesis L9	COG0359	J	Translation, ribosomal structure and biogenesis
**SA0486**	*gltX*	N315	0.18	0.18	glutamyltRNA synthetase	COG0008	J	Translation, ribosomal structure and biogenesis
**SA0564**	*argS*	N315	0.19	0.17	arginyltRNA synthetase	COG0018	J	Translation, ribosomal structure and biogenesis
**SA2046**	*rplD*	N315	0.20	0.20	50S Protein synthesis L4		J	Translation, ribosomal structure and biogenesis
**SA2048**	*rpsJ*	N315	0.20	0.28	30S Protein synthesis S10	COG0051	J	Translation, ribosomal structure and biogenesis
**SA2024**	*rpsK*	N315	0.21	0.26	30S Protein synthesis S11	COG0100	J	Translation, ribosomal structure and biogenesis
**SA1106**	*proS*	N315	0.22	0.29	prolinetRNA ligase	COG0442	J	Translation, ribosomal structure and biogenesis
**SA0352**	*rpsF*	N315	0.24	0.28	30S Protein synthesis S6	COG0360	J	Translation, ribosomal structure and biogenesis
**SA1920**	*prfA*	N315	0.25		peptide chain release factor 1	COG0216	J	Translation, ribosomal structure and biogenesis
**MW1076**	*ileS*	MW2	0.26	0.18	Ile tRNA synthetase	COG0060	J	Translation, ribosomal structure and biogenesis
**SA2016**	*rpsI*	N315	0.26	0.16	30S Protein synthesis S9		J	Translation, ribosomal structure and biogenesis
**SA1471**	*rpmA*	N315	0.29		50S Protein synthesis L27		J	Translation, ribosomal structure and biogenesis
**SA1504**	*infC*	N315	0.33		translation initiation factor IF3	COG0290	J	Translation, ribosomal structure and biogenesis
**SA1082**	*rimM*	N315	0.33		probable 16S rRNA processing protein	COG0806	J	Translation, ribosomal structure and biogenesis
**SA1113**	*rbfA*	N315	0.33		ribosomebinding factor A	COG0858	J	Translation, ribosomal structure and biogenesis
**SA1414**	*rpsT*	N315	3.12		30S Protein synthesis S20	COG0268	J	Translation, ribosomal structure and biogenesis
**SA1922**	*rpmE*	N315	3.75		50S Protein synthesis L31	COG0254	J	Translation, ribosomal structure and biogenesis
**SA1704**	*map*	N315	3.98	3.72	methionyl aminopeptidase map	COG0024	J	Translation, ribosomal structure and biogenesis
**MW0318**	*MW0318*	MW2		3.30	ribosomalproteinserine Nacetyltransferase	COG1670	J	Translation, ribosomal structure and biogenesis
**MW1696**	*MW1696*	MW2		0.11	16S pseudouridylate synthase	COG1187	J	Translation, ribosomal structure and biogenesis
**SA1557**	*ccpA*	N315	0.29	0.29	catabolite control protein A	COG1609	K	Transcription
**SA0726**	*gapR*	N315	0.31		glycolytic operon regulator	COG2390	K	Transcription
**SA1139**	*glpP*	N315	0.15	0.12	glycerol uptake operon antiterminator regulatory protein	COG1954	K	Transcription
**SA1925**	*HP*	N315	0.23	0.22	Hypothetical protein	COG1733	K	Transcription
**SA1868**	*HP*	N315	0.28	0.21	Hypothetical protein	COG2183	K	Transcription
**SA1109**	*nusA*	N315	0.05	0.20	transcription terminationantitermination factor	COG0195	K	Transcription
**SA0494**	*nusG*	N315	0.23	0.26	transcription antitermination protein	COG0250	K	Transcription
**SA1923**	*rho*	N315	0.23	0.14	transcription termination factor Rho	COG1158	K	Transcription
**SA0735**	*rnr*	N315	0.17	0.23	ribonuclease R	COG0557	K	Transcription
**SA2023**	*rpoA*	N315	0.10		DNA directed RNA polymerase alpha chain	COG0202	K	Transcription
**SA0500**	*rpoB*	N315	0.07	0.32	RNA polymerase beta chain	COG0085	K	Transcription
**SA0501**	*rpoC*	N315	0.16	0.27	RNA polymerase betaprime chain	COG0086	K	Transcription
**SA0337**	*SA0337*	N315	3.91		transcriptional repressor	COG1476	K	Transcription
**SA0492**	*SA0492*	N315	0.07		HP	COG1595	K	Transcription
**SA0590**	*SA0590*	N315	4.50	3.47	iron dependent repressor	COG1321	K	Transcription
**SA1355**	*SA1355*	N315	0.08	0.14	transcription termination factor	COG0781	K	Transcription
**SA1748**	*SA1748*	N315	0.12		transcription regulator, GntR family	COG1725	K	Transcription
**SA2002**	*SA2002*	N315	3.16	4.33	transcription regulator MerR family	COG0789	K	Transcription
**SA2108**	*SA2108*	N315	0.28		transcription regulator, RpiR family	COG1737	K	Transcription
**SA2165**	*SA2165*	N315	0.15	0.18	transcriptional regulator tetR family	COG1309	K	Transcription
**SA2174**	*SA2174*	N315	3.34	4.29	transcriptional regulator	COG1846	K	Transcription
**SA2296**	*SA2296*	N315	3.32		transcriptional regulator, MerR family	COG0789	K	Transcription
**SA2330**	*SA2330*	N315	4.31	3.61	transcription regulator	COG0583	K	Transcription
**SA2358**	*SA2358*	N315	4.26	3.57	transcriptional regulator (TetR/AcrR family)	COG1309	K	Transcription
**SA2429**	*SA2429*	N315	3.95		arginine repressor	COG1438	K	Transcription
**SA2498**	*SA2498*	N315	0.10	0.15	DNAbinding protein Spo0Jlike homolog	COG1475	K	Transcription
**SA0108**	*sarS*	N315	4.08	4.20	sarS	COG1846	K	Transcription
**SA1869**	*sigB*	N315	0.11	0.15	sigma factor B	COG1191	K	Transcription
**SA1874**	*alr*	N315	0.21	0.23	alanine racemase	COG0787	M	Cell wall/membrane/envelope biogenesis
**MW0136**	*cap8M*	MW2	0.07	0.23	capsular polysaccharide synthesis enzyme Cap8M	COG2148	M	Cell wall/membrane/envelope biogenesis
**SA0150**	*capG*	N315	0.15	0.24	capsular polysaccharide synthesis enzyme Cap5G	COG0381	M	Cell wall/membrane/envelope biogenesis
**SA0152**	*capI*	N315	3.16		capsular polysaccharide synthesis enzyme Cap5I	COG0438	M	Cell wall/membrane/envelope biogenesis
**SA1887**	*ddlA*	N315	0.33		D-alanine D-alanine ligase	COG1181	M	Cell wall/membrane/envelope biogenesis
**SA1027**	*div1b*	N315	0.05	0.11	cell division protein, FtsQ homolog	COG1589	M	Cell wall/membrane/envelope biogenesis
**SA0794**	*dltB*	N315	0.04	0.10	DltB membrane protein	COG1696	M	Cell wall/membrane/envelope biogenesis
**SA0796**	*dltD*	N315	0.16	0.18	poly Dalanine transfer protein	COG3966	M	Cell wall/membrane/envelope biogenesis
**SA2499**	*gidB*	N315	0.12	0.32	glucose inhibited division protein B	COG0357	M	Cell wall/membrane/envelope biogenesis
**SA2459**	*icaA*	N315	3.92	3.64	intercellular adhesion protein A	COG1215	M	Cell wall/membrane/envelope biogenesis
**SA2461**	*icaB*	N315	3.75		intercellular adhesion protein B		M	Cell wall/membrane/envelope biogenesis
**SA1413**	*lepA*	N315	0.26	0.27	GTPbinding protein	COG0481	M	Cell wall/membrane/envelope biogenesis
**SA0265**	*lytM*	N315	14.88		peptidoglycan hydrolase	COG0739	M	Cell wall/membrane/envelope biogenesis
**SA0038**	*mecA*	N315	3.32	3.50	penicillin binding protein 2'	COG0768	M	Cell wall/membrane/envelope biogenesis
**SA1614**	*menC*	N315		0.23	osuccinylbenzoic acid synthetase	COG4948	M	Cell wall/membrane/envelope biogenesis
**SA1913**	*mnaA*	N315	0.01	0.07	UDPGlcNAc 2epimerase	COG0381	M	Cell wall/membrane/envelope biogenesis
**SA1025**	*mraY*	N315	0.22	0.26	phosphoNmuramic acidpentapeptide translocase	COG0472	M	Cell wall/membrane/envelope biogenesis
**SA1902**	*murA*	N315	0.04	0.23	UDPNacetylglucosamine 1carboxyvinyl transferase 1	COG0766	M	Cell wall/membrane/envelope biogenesis
**SA1561**	*murC*	N315	0.10	0.19	UDPNAacerylmuramatealanine ligase	COG0773	M	Cell wall/membrane/envelope biogenesis
**SA1026**	*murD*	N315	0.04	0.08	UDPNacetylmuramoylalanineDglutamate ligase	COG0771	M	Cell wall/membrane/envelope biogenesis
**SA1886**	*murF*	N315	0.07	0.08	UDP-N-Ac mur-D-glu-diaminopimelate-Dala-ala ligase	COG0770	M	Cell wall/membrane/envelope biogenesis
**SA1251**	*murG*	N315	0.28	0.31	Undecaprenyl PPMurNAc-UDPGlcNAc GlcNAc transferase	COG0707	M	Cell wall/membrane/envelope biogenesis
**SA0997**	*murI*	N315	0.09	0.11	glutamate racemase	COG0796	M	Cell wall/membrane/envelope biogenesis
**SA1926**	*murZ*	N315	0.14	0.17	UDPNacetylglucosamine 1carboxylvinyl transferase 2	COG0766	M	Cell wall/membrane/envelope biogenesis
**SA1183**	*opuD*	N315	0.30	0.32	glycine betaine transporter	COG1292	M	Cell wall/membrane/envelope biogenesis
**SA1283**	*pbp2*	N315	0.15	0.17	PBP2	COG0744	M	Cell wall/membrane/envelope biogenesis
**SA1381**	*pbp3*	N315	0.23	0.31	penicillinbinding protein 3	COG0768	M	Cell wall/membrane/envelope biogenesis
**SA0125**	*SA0125*	N315	3.62		Eps (Exopolysaccharide) G	COG0438	M	Cell wall/membrane/envelope biogenesis
**SA0243**	*SA0243*	N315	0.11		teichoic acid biosynthesis protein B	COG1887	M	Cell wall/membrane/envelope biogenesis
**SA0247**	*SA0247*	N315		0.09	teichoic acid biosynthesis protein B	COG1887	M	Cell wall/membrane/envelope biogenesis
**SA0248**	*SA0248*	N315	0.13	0.17	betaglycosyltransferase	COG0463	M	Cell wall/membrane/envelope biogenesis
**SA0674**	*SA0674*	N315	0.12	0.20	anionbinding protein	COG1368	M	Cell wall/membrane/envelope biogenesis
**SA0693**	*SA0693*	N315	0.33		UDPN acetylenol pyruvoyl glucosamine reductase	COG0812	M	Cell wall/membrane/envelope biogenesis
**SA1231**	*SA1231*	N315		0.10	alanine racemase	COG0787	M	Cell wall/membrane/envelope biogenesis
**SA1291**	*SA1291*	N315		0.30	lipopolysaccharide biosynthesis related pr homolog	COG0438	M	Cell wall/membrane/envelope biogenesis
**SA1708**	*SA1708*	N315	0.24	0.20	UDPNacetylmuramyl tripeptide synthetase homolog	COG0769	M	Cell wall/membrane/envelope biogenesis
**SAV0913**	*SAV0913*	Mu50	6.29		amidase	COG5632	M	Cell wall/membrane/envelope biogenesis
**SA1551**	*sgtA*	N315	7.46	7.75	probable transglycosylase	COG0744	M	Cell wall/membrane/envelope biogenesis
**SA0456**	*spoVG*	N315	5.95		stage V sporulation protein G homologue	COG2088	M	Cell wall/membrane/envelope biogenesis
**SA0595**	*tagB*	N315		0.15	teichoic acid biosynthesis protein B	COG1887	M	Cell wall/membrane/envelope biogenesis
**SA0880**	*SA0880*	N315	0.05	0.04	Na+transporting ATP synthase	COG0168	P	Inorganic ion transport and metabolism
**SA0587**	*SA0587*	N315	0.08	0.10	lipoprotein, Streptococcal adhesin PsaA homologue	COG0803	P	Inorganic ion transport and metabolism
**SA2137**	*SA2137*	N315	0.08	0.31	divalent cation transport	COG0598	P	Inorganic ion transport and metabolism
**SA1384**	*SA1384*	N315	0.12		ABC transporter	COG1108	P	Inorganic ion transport and metabolism
**SA0589**	*SA0589*	N315	0.15		ABC transporter ATPbinding protein	COG1121	P	Inorganic ion transport and metabolism
**SA0603**	*fhuB*	N315	0.16	0.24	ferrichrome transport permease	COG0609	P	Inorganic ion transport and metabolism
**SA0580**	*SA0580*	N315	0.18		Na+/H+ antiporter	COG1006	P	Inorganic ion transport and metabolism
**SA2072**	*modC*	N315	0.20		molybdenum transport ATPbinding protein ModC	COG4148	P	Inorganic ion transport and metabolism
**SA1329**	*SA1329*	N315	0.22	0.26	ferric uptake regulator homolog	COG0735	P	Inorganic ion transport and metabolism
**SA1238**	*SA1238*	N315	0.23	0.20	tellurite resistance protein	COG3853	P	Inorganic ion transport and metabolism
**SA1592**	*SA1592*	N315	0.25	0.16	aesenical pump membrane protein homolog	COG1055	P	Inorganic ion transport and metabolism
**SA0136**	*SA0136*	N315	0.25	0.29	phosphonates transport permease	COG3639	P	Inorganic ion transport and metabolism
**SA1170**	*katA*	N315	0.25	0.32	Catalase	COG0753	P	Inorganic ion transport and metabolism

ORF #	Common	Organism	2h fold change	6h fold change	Protein name	GO	Class	Annotation
**SA0137**	*SA0137*	N315	0.25	0.18	transport system protein	COG3638	**P**	Inorganic ion transport and metabolism
**SA0688**	*SA0688*	N315	0.26		ferrichrome ABC transporter permease	COG4606	**P**	Inorganic ion transport and metabolism
**SA0956**	*SA0956*	N315	0.28	0.32	Mn2+transport protein	COG1914	**P**	Inorganic ion transport and metabolism
**SA0690**	*SA0690*	N315	0.28	0.24	ferrichrome ABC transporter ATPbinding protein	COG4604	**P**	Inorganic ion transport and metabolism
**SA1979**	*SA1979*	N315	0.30	0.26	HP, similar toferrichrome ABC transporter (binding protein)	COG4594	**P**	Inorganic ion transport and metabolism
**MW1714**	*MW1714*	MW2	0.30		arsenical pump membrane protein homolog	COG1055	**P**	Inorganic ion transport and metabolism
**SA2073**	*modB*	N315	0.31		probable molybdenum transport permease	COG4149	**P**	Inorganic ion transport and metabolism
**SA1978**	*SA1978*	N315	0.33	0.31	ferrichrome ABC transporter (permease)	COG0609	**P**	Inorganic ion transport and metabolism
**SA1218**	*pstB*	N315	3.12	4.22	phosphate ABC transporter, ATPbinding protein	COG1117	**P**	Inorganic ion transport and metabolism
**SA2369**	*SA2369*	N315	3.26		ferrous iron transporter protein B	COG0370	**P**	Inorganic ion transport and metabolism
**SA0981**	*isdF*	N315	3.34		ferrichrome ABC transporter	COG0609	**P**	Inorganic ion transport and metabolism
**SA0759**	*SA0759*	N315	3.39		arsenate reductase	COG1393	**P**	Inorganic ion transport and metabolism
**SA1941**	*dps*	N315	3.40	3.66	general stress protein 20U	COG0783	**P**	Inorganic ion transport and metabolism
**SA0168**	*SA0168*	N315	3.42	3.34	probable permease of ABC transporter	COG0600	**P**	Inorganic ion transport and metabolism
**SA1678**	*SA1678*	N315	3.50	3.03	transcription regulator Fur family homolog	COG0735	**P**	Inorganic ion transport and metabolism
**SA0111**	*sirA*	N315	3.53		lipoprotein	COG0614	**P**	Inorganic ion transport and metabolism
**SA0422**	*SA0422*	N315	3.69	3.64	lactococcal lipoprotein	COG1464	**P**	Inorganic ion transport and metabolism
**SA1382**	*sodA*	N315	3.86	3.14	superoxide dismutase SodA	COG0605	**P**	Inorganic ion transport and metabolism
**SA0567**	*SA0567*	N315	5.00		iron(III) ABC transporter permease protein	COG0609	**P**	Inorganic ion transport and metabolism
**MW0200**	*MW0200*	MW2	45.05	25.06	periplasmicironbinding protein BitC	COG1840	**P**	Inorganic ion transport and metabolism
**MW0115**	*MW0115*	MW2		0.28	phosphonates transport permease	COG3639	**P**	Inorganic ion transport and metabolism
**MW2102**	*MW2102*	MW2		0.09	ferrichrome ABC transporter (permease)	COG0609	**P**	Inorganic ion transport and metabolism
**SA0583**	*mprF*	N315		0.22	Na+/H+ antiporter	COG2212	**P**	Inorganic ion transport and metabolism
**SA0808**	*mnhF*	N315		0.21	Na+/H+ antiporter subunit	COG2212	**P**	Inorganic ion transport and metabolism
**SA1220**	*SA1220*	N315		3.03	phosphate ABC transporter	COG0573	**P**	Inorganic ion transport and metabolism
**SA1385**	*SA1385*	N315		0.30	ABC transporter ATPbinding protein	COG1121	**P**	Inorganic ion transport and metabolism
**SA1948**	*czrB*	N315		0.33	cation efflux system membrane protein homolog	COG1230	**P**	Inorganic ion transport and metabolism
**SA1958**	*SA1958*	N315		3.12	transposase for IS232	COG1119	**P**	Inorganic ion transport and metabolism
**SA2228**	*SA2228*	N315		3.13	NA(+)/H(+) exchanger	COG0025	**P**	Inorganic ion transport and metabolism
**SA1844**	*agrA*	N315	0.10	0.12	AgrA	COG3279	**T**	Signal transduction mechanisms
**SA1843**	*agrC*	N315	0.01	0.02	AgrC	COG2972	**T**	Signal transduction mechanisms
**SA1246**	*arlS*	N315	0.33		putative protein histidine kinase ArlS	COG0642	**T**	Signal transduction mechanisms
**SA0715**	*hprK*	N315	0.16	0.16	HPr kinase/phosphatase	COG1493	**T**	Signal transduction mechanisms
**SA1882**	*kdpD*	N315	0.05	0.21	sensor protein KdpD	COG2205	**T**	Signal transduction mechanisms
**SA0067**	*kdpD*	N315	5.81	3.91	kdp operon sensor protein	COG2205	**T**	Signal transduction mechanisms
**SA1400**	*phoH*	N315	0.18	0.21	phosphate starvationinduced protein phoH homolog	COG1702	**T**	Signal transduction mechanisms
**SA1872**	*rsbU*	N315	0.05	0.25	sigmaB regulation protein RsbU	COG2208	**T**	Signal transduction mechanisms
**SA1871**	*rsbV*	N315	0.15	0.19	anti sigmaB factor antagonist	COG1366	**T**	Signal transduction mechanisms
**SA1870**	*rsbW*	N315	0.15	0.33	anti sigmaB factor	COG2172	**T**	Signal transduction mechanisms
**SA0215**	*SA0215*	N315	3.60		two component response regulator	COG4753	**T**	Signal transduction mechanisms
**SA0614**	*SA0614*	N315		0.22	two component response regulator	COG0745	**T**	Signal transduction mechanisms
**SA1540**	*SA1540*	N315		3.14	hypothetical protein	COG1956	**T**	Signal transduction mechanisms
**SA1667**	*SA1667*	N315	0.11	0.23	two component sensor histidine kinase	COG2203,COG4585	**T**	Signal transduction mechanisms
**SA2179**	*SA2179*	N315	0.20	0.22	response regulators of two component regulatory	COG2197	**T**	Signal transduction mechanisms
**SA0661**	*saeR*	N315	0.04	0.24	response regulator	COG0745	**T**	Signal transduction mechanisms
**SA0660**	*saeS*	N315	0.04	0.12	histidine protein kinase	COG0642	**T**	Signal transduction mechanisms
**SA1323**	*srrA*	N315	0.10	0.23	staphylococcal respiratory response protein	COG0745	**T**	Signal transduction mechanisms
**SA1322**	*srrB*	N315	0.10	0.14	staphylococcal respiratory response protein	COG0642	**T**	Signal transduction mechanisms
**SA2180**	*SA2180*	N315	0.01	0.07	two component sensor histidine kinase	COG4585,COG0642	**T**	Signal transduction mechanisms
**SA1701**	*vraS*	N315		0.19	two component sensor histidine kinase	COG4585	**T**	Signal transduction mechanisms

* Functional categories are colored following COG code, C (Energy production and conversion) appears in grey, J (Translation, ribosomal structure and biogenesis) in pink, K (Transcription) in orange, M (Cell wall/membrane/envelope biogenesis) in yellow, T (Signal transduction mechanisms) in light bleu. For clarity, all genes involved in transports and metabolism appear in green.
